# Nutrient stress diverts RRN3 from rRNA transcription to alternative polyadenylation of autophagy mRNAs in ovarian cancer

**DOI:** 10.1038/s41419-025-08142-6

**Published:** 2025-11-21

**Authors:** Jianying Lv, Shuo Wang, Tianxiang Liu, Yi Liu, Yuting Bai, Wei-Ao Qu, Jixuan Ding, Zhiqiang Chen, Yanhua Liu, Yanan Chen, Jia Li, Jian Li, Wei Ding, Yongjun Piao, Rong Xiang, Beilei Zeng, Longlong Wang, Yi Shi

**Affiliations:** 1 The School of Medicine, Nankai University, Tianjin, China; 2 Department of Urology, Tianjin Union Medical Center, The First affiliated Hospital of Nankai University, Tianjin, China; 3 Department of Gynecological Oncology, Tianjin Central Hospital of Obstetrics and Gynecology, Tianjin, China; 4 Department of Oncology, Affiliated Hospital of North Sichuan Medical College, Nanchong, Sichuan China

**Keywords:** Autophagy, Transcription, Cancer metabolism

## Abstract

Stress-induced alternative processing of mRNA is emerging as an essential mechanism to drive almost every hallmark of cancer. Through a genome-wide screening based on an abnormal transcriptional readthrough event favoring the malignant progression of ovarian carcinoma (OC), we identified novel mRNA processing regulators including RRN3, an essential factor for the transcriptional initiation of rRNA. The long-read RNA sequencing and PAR-CLIP analyses revealed that RRN3 was involved in the usage of alternative polyadenylation (APA) sites, resulting in the altered stability of autophagy-related mRNAs. More interestingly, we discovered that nutrient-deprivation-induced phosphorylation of RRN3 at serine 199 was sufficient to divert RRN3 out of the nucleolus to the nuclear plasma, where RRN3 regulated the APA of autophagy mRNAs, such as OPTN, to enhance their stability and eventually promoted autophagy. Further in vivo experiments showed that nutrient-stress-triggered switch of RRN3 from rRNA transcription to APA regulation was essential for the growth and dissemination of OC in mice.

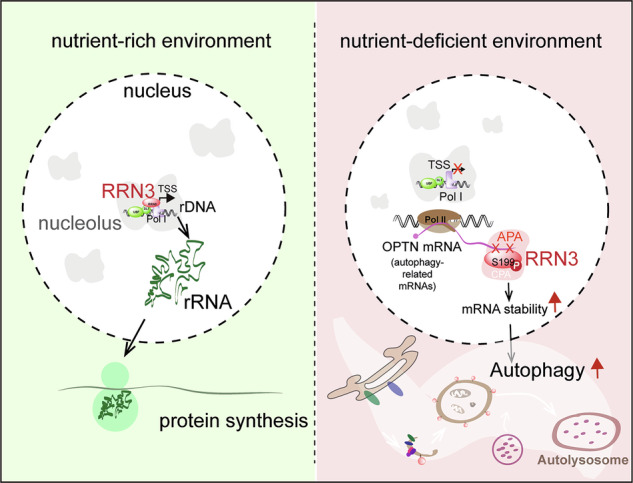

## Introduction

RRN3, also known as TIFIA, is a crucial transcription initiation factor of RNA polymerase I (Pol I) complex for the synthesis of precursor ribosomal RNA (pre-rRNA) transcripts. Approximately 80% of the energy is dedicated to ribosome synthesis, in which rRNA transcription constitutes 60% of total transcription, in proliferating tumor cells to meet their robust protein synthesis demand [1–3]. Notably, the presence of abnormal structure of nucleolus, where rRNA is transcribed, has been identified as a hallmark of cancer cells [4]. RRN3 is tightly regulated by phosphorylation at multiple sites to adjust the rRNA synthesis process to maintain the cellular energy homeostasis under environmental stresses [5–7]. Oxidative stress-activated JNK2 is able to phosphorylate RRN3 at the threonine 200 residue (T200) to cause its translocation from the nucleolus to the extranuclear region [7]. And the treatment with rapamycin, a mTOR (mechanistic target of rapamycin) inhibitor often used to mimic the nutrient stress, has been reported to increase the phosphorylation of RRN3 at serine 199 residue (S199), resulting in its dissociation from the Pol I complex and re-localization in the cells with unknown biological roles [5, 8].

Alternative RNA processing, including alternative splicing (AS), alternative polyadenylation (APA) and transcription initiation, is a key mechanism that generates multiple transcript variants and hence protein isoforms from a single gene, which expands the coding capacity of genomes during the evolution of multicellular organisms and drives phenotypic diversity for the environmental adaption for not only normal cells, but also cancer cells [9]. Cancer cells have distinctive splicing and polyadenylation features, contributing to almost every hallmark of cancer [10–13]. Most mammalian genes include multiple APA sites, which allows a single gene to generate numerous mRNA transcripts harboring 3’ UTR of different length, leading to altered intracellular localization, mRNA stability, and translation efficiency [14, 15]. APA-mediated shortening of the 3’UTR can disrupt the regulation of oncogenes and tumor suppressors. Conversely, the generation of longer 3′ UTRs through APA may also play a role in cancer phenotypes [16]. The regulation of APA involves several interconnected processes, including transcription, pre-mRNA cleavage and post-transcriptional processes. However, the detailed mechanisms of APA regulation remain elusive, which have been shown to be valuable targets for cancer treatment [17].

Autophagy is an evolutionarily conserved cellular process to maintain cellular homeostasis by degrading damaged or ‘unnecessary’ cytoplasmic components under many different sorts of stress [18, 19], and dysregulated autophagy is involved in many pathological processes, including cancer [20–23]. Increasing lines of evidence has revealed that alternative RNA processing is deeply involved in the regulation of autophagy [24]. Mutations in the splicing machinery component U2AF1 have been linked to abnormal distal alternative polyadenylation (APA) site selection of the autophagy-related factor ATG7, leading to defective autophagy and promoting the malignant transformation of mouse pro-B cells [25]. The aberrant AS of ULK1 (unc-51 like autophagy activating kinase 1), a key initiation factor capable of being phosphorylated by mTOR and AMPK at distinct residues for autophagy induction, has been linked to autosomal dominant retinitis pigmentosa caused by the deficiency of splicing factor PRPF8 [24]. And many core components of the autophagic machinery exist in multiple alternative RNA isoforms that modulate autophagy differently. For example, various isoforms of ATG proteins generated by AS or APA, e.g., ATG14, ATG12, ATG16L1, ATG10, exhibit diverse effects on the autophagic process [24]. OPTN (optineurin), a multifunctional cargo receptor playing various roles in autophagosome nucleation, maturation, lysosomal quality control, and degradation, is also regulated by AS [26]. The splice variant of OPTN, known as d157mOPTN, is unable to promote autophagosome formation during starvation-induced autophagy, thus impairing the process. However, how alternative RNA processing is regulated to maintain cellular homeostasis through autophagy under environmental stress remains largely unknown.

To systematically identify key RNA processing regulators involved in the progression of ovarian carcinoma (OC), we performed a genome-wide gene knockout screening based on a previously identified OC-promoting transcriptional readthrough (TRT) event caused by dysregulated transcriptional termination of tumor suppressor gene COMMD3 which leads to the fusion of COMMD3 with downstream oncogene BMI1 [27]. We identified new mRNA processing regulators involved in Pol I and Pol III-related transcription factors and stress responses, among which RRN3 ranked as the first candidate TRT-promoting factor. And our further studies revealed a novel elegant autophagy-regulating mechanism by evoking the moonlight function of RRN3 at the alternative mRNA processing layer, which switched off the energy-consuming rRNA synthesis and promoted autophagy under nutrient stress.

## Results

### Genome-wide gene knockout screen in OC reveals RRN3 as a regulator of the termination of mRNA transcription

**Fig. 1:**
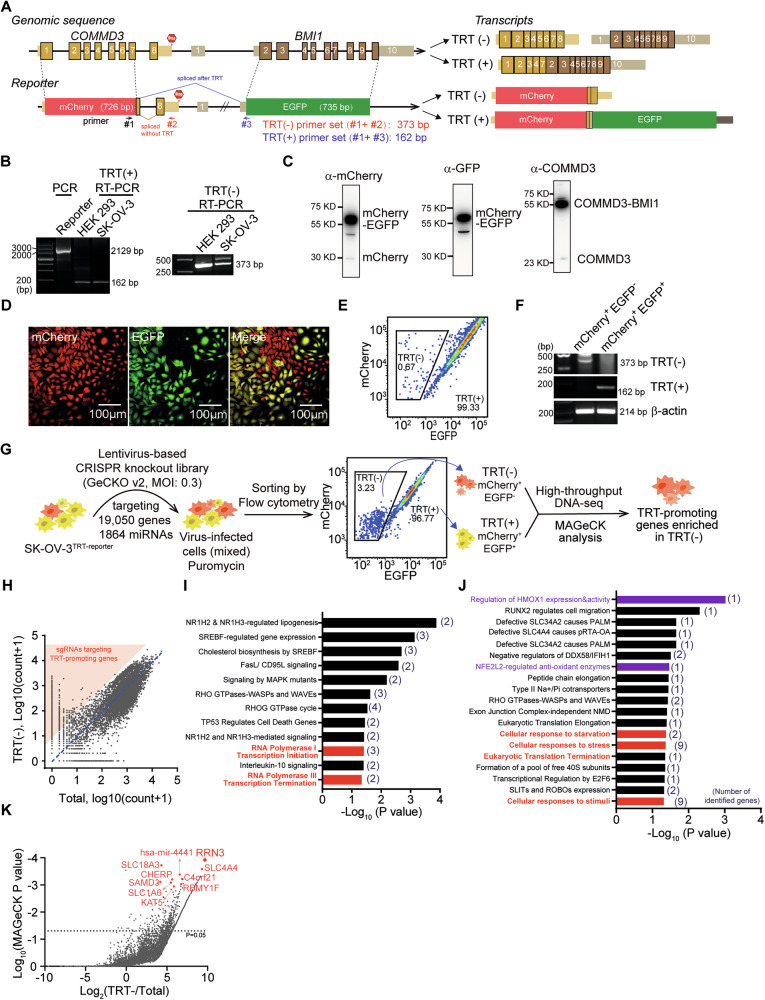
Genome-wide gene knockout screening to identify regulators of transcription termination. **A** The schematic diagram of the dual-fluorescence transcriptional-readthrough (TRT) reporter system. The exons are indicated by boxes with numbers, where the coding areas are highlighted in thicker boxes. The gene structures and naturally occurring read-through transcription between the neighboring COMMD3 and BMI1 genes (upper panel) is used as a template for the design of TRT reporter (bottom panel), where the coding sequences of COMMD3 and BMI1 are replaced by mCherry- and EGFP-encoding sequences respectively. The primer sets for identification of TRT are shown by arrows. **B** RT-PCR analysis of the efficiency of TRT reporter transfected in HEK 293T and SK-OV-3 cells. **C** Western blot analysis of the fusion protein products generated by TRT reporter and the TRT of the endogenous COMMD3 and BMI1 genes in SK-OV-3 cells. **D** Fluorescent images of the SK-OV-3 cells stably transfected with the TRT reporter (SK-OV-3-TRT), in which the TRT-positive cells are yellow (mCherry^+^EGFP^+^) and TRT-negative cells are red (mCherry^+^EGFP^-^). **E**, **F** Flow cytometry-based sorting and quantification of TRT^+^ and TRT^-^ SK-OV-3 cell populations (**E**), which were further confirmed by RT-PCR analysis (**F**). **G** CRISPR/Cas9 screening pipeline. SK-OV-3-TRT cells were infected with the pooled lentiviral human GeCKO (v2.0) sgRNA library followed by puromycin selection. TRT^-^ or TRT^+^ cell populations were sorted using flow cytometry and subjected to sgRNA identification by high throughput DNA sequencing. **H** The distribution of sgRNAs identified in TRT^-^ cells and the total cells. **I**, **J** The pathway enrichment analysis of the top 200 genes (**I**) and the top 100 genes (**J**) using REACTOME. **K** The MAGeCK algorithm was used to analyze sgRNA enrichment and the top 10 genes ranked by p value were highlighted in red.

To systematically identify key genes regulating the transcriptional termination of mRNA in OC, we firstly designed a dual-fluorescent reporter system based on a previously identified oncogenic transcriptional readthrough (TRT) event that led to the fusion of the transcripts of tumor suppressor gene COMMD3 and the downstream oncogene BMI1. As shown in Fig. 1A, we replaced the coding sequences of COMMD3 and BMI1 with those of the red fluorescent protein mCherry and enhanced green fluorescent protein EGFP, respectively, while kept the intergenic region and neighboring exons and introns given the existence of possible cis-elements in the region and the coordination between splicing and polyadenylation. We stably transfected the reporter into HEK 293 T cells and human OC cells SK-OV-3 and successfully detected the transcripts with or without TRT by RT-PCR (Fig. 1B) and their protein products (Fig. 1C), showing that the high TRT efficiency of the reporter in SK-OV-3 cells (SK-OV-3^TRT^) was comparable to the endogenous COMMD3 and COMMD3-BMI1. Further fluorescent microscopy and flow cytometry analysis revealed that TRT rate in SK-OV-3 cells was around 99.33% (Fig. 1D, E). The TRT-negative(mCherry^+^EGFP^-^) and TRT-positive(mCherry^+^EGFP^+^) cells of SK-OV-3^TRT^ cells were sorted by flow cytometry and were verified by RT-PCR (Fig. 1F).

We next transfected the lentivirus-based CRISPR/Cas9 library (GeCKO-v2.0, made by Feng Zhang’s laboratory) against 19,050 protein-encoding genes (6 sgRNAs per gene) and 1864 miRNA (4 sgRNAs per miRNA) [28], into SK-OV-3^TRT^ at a ratio (with the multiplicity of infection (MOI) ~ 0.3) to generate single-gene-deficient cell population, among which the cells with enhanced or inhibited transcriptional termination between mCherry and EGFP(i.e., mCherry^+^EGFP^-^ and mCherry^+^EGFP^+,^ respectively) could be sorted by flow cytometry for further deep DNA sequencing analysis to identify the key regulators of transcriptional termination (Fig. 1G and H). The enrichment of sgRNAs and target genes were analyzed by the MAGeCK algorithm (Supplementary File 1) and the functional classification of the top 200 genes and the top 100 genes were performed by using the REACTOME pathway analysis tool (Fig. 1I and J, Supplementary File 2), we identified genes involved in the regulation of RNA polymerase I transcription initiation (i.e., RRN3, RBBP4, POLR2F), RNA polymerase III transcription termination (i.e., NFIC and POLR2F) and cellular responses to stresses (i.e., PSMD10, KLHDC3, KAT5, RBBP4, UBE2D2, DCTN3, RRAGD, RPL37A, HMOX1). Specifically, among the top 10 genes, we obtained direct regulators of transcription involving RRN3, CHERP and KAT5 (Fig. 1K).

### RRN3 regulates the termination of RNA polymerase II transcription

**Fig. 2:**
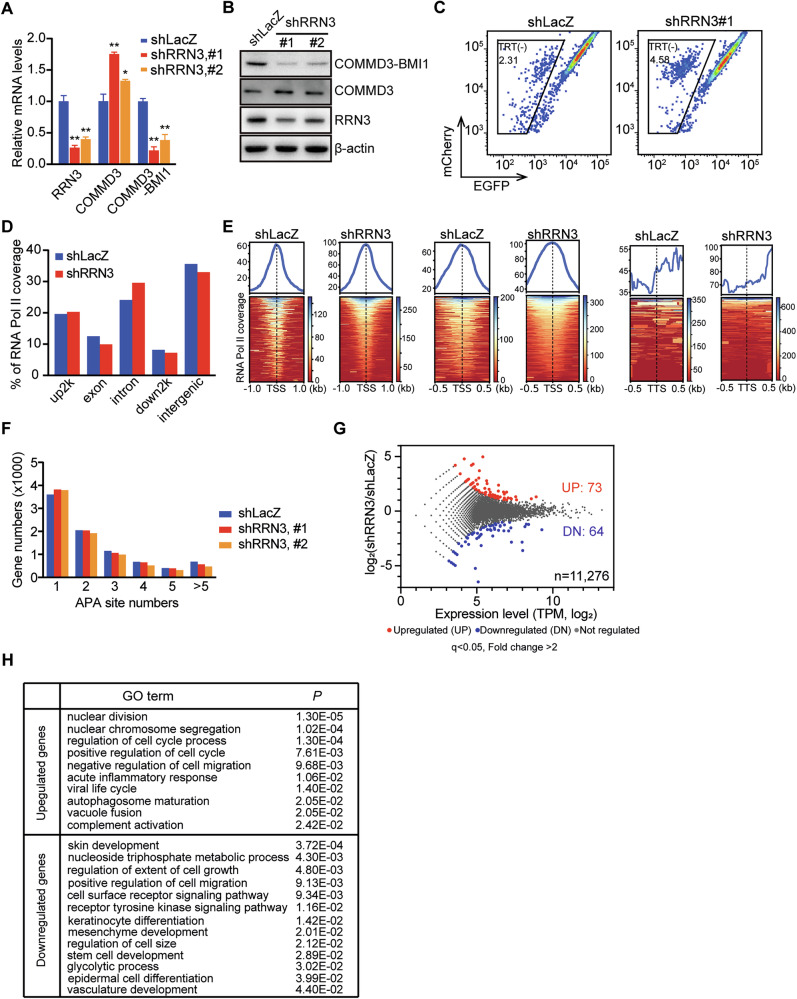
RRN3 regulates the termination of RNA polymerase II (Pol II) transcription. RT-qPCR (**A**) and western blot (**B**) analyses show that silencing RRN3 enhances the transcription termination of COMMD3, leading to increased COMMD3 expression and decreased COMMD3-BMI1 fusion product in SK-OV-3 cells (means ± SD, **p < 0.01, *p < 0.05, by Student’s t-test). **C** Flow cytometry analysis of RRN3-affected TRT in SK-OV-3-TRT reporter cells. **D** Metagene analysis of ChIP-seq-identified Pol II coverage change caused by silencing RRN3 in SK-OV-3 cells. **E** Metagene analysis of Pol II coverage near the transcription start sites (TSS) and termination sites (TTS). (**F**) Bar graph showing the numbers of genes with indicated alternative polyadenylation (APA) sites identified by long-read RNA sequencing in SK-OV-3 cells. Scatter plot showing gene expression level change versus expression level upon RRN3 silencing in SK-OV-3 cells (**G**) and gene ontology (GO) terms related to upregulated and downregulated genes in RRN3-silenced cells (**H**).

Although the top transcription regulator RRN3 has been well established as an essential transcription initiation factor of RNA polymerase I in the nucleolus, increasing evidences show its translocation out of the nucleolus under the environmental cues including nutrient deficiency, oxidative stress, etc. [5, 7, 8], suggesting the existence of potential novel roles of RRN3 in the cellular stress responses. We therefore investigated if RRN3 was able to influence the termination of RNA polymerase II (Pol II) transcription in the nuclear plasma. In SK-OV-3 cells, knocking down RRN3 caused increased level of COMMD3 transcripts and significantly decreased fusion transcripts of COMMD3-BMI1 (Fig. 2A), resulting in increased production of COMMD3 protein and decreased fusion protein of COMMD3-BMI1 (Fig. 2B). Knocking down RRN3 in SK-OV-3^TRT^ reporter cells also showed increased rate of transcriptional termination which generates mCherry^+^ EGFP^-^ cell population (Fig. 2C), suggesting the potential role of RRN3 in the regulation of the termination of Pol II transcription. We next performed the genome-wide Pol II occupancy by chromatin immunoprecipitation followed by deep DNA sequencing (ChIP-Seq) to investigate the influence of RRN3 on Pol II transcription process (Supplementary Fig. 1A and B, Supplementary File 3). Metagene analysis showed that knocking down RRN3 caused slightly increased Pol II coverage in the upstream (within 2 kilobase pairs) and intron regions of protein-coding genes, while reduced coverage in the exon, downstream (~2 kilobase pairs) and intergenic regions (Fig. 2D). Specifically, knocking down RRN3 altered the Pol II coverage mostly at the transcriptional termination sites (TTS) when compared with that at the transcriptional start site (TSS), suggesting the potential roles of RRN3 in the regulation of Pol II transcription termination (Fig. 2E).

To further explore the influence of RRN3 on Pol II-transcribed transcripts, we performed full-length transcript sequencing using PacBio ISO-Seq platform to more accurately analysis transcript variants generated by alternative splicing or alternative polyadenylation (APA) (Supplementary Fig. 2A, Supplementary File 4). In general, we didn’t observe the significant alteration of the alternative splicing events (Supplementary Fig. 2B) and transcript fusion of non-neighboring genes (Supplementary Fig. 2C). However, knocking down RRN3 caused increased number of genes with only one APA sites, while less genes with multiple APA sites (Fig. 2F). In addition, knocking down RRN3 significantly changed the mRNA level of 137 genes, with 73 genes being up-regulated and 64 genes being down-regulated (Fig. 2G), which were involved in a variety of biological processes such as cell cycle, cell growth and autophagy (Fig. 2H, Supplementary Fig. 2D).

### RRN3 interacts with autophagy-related mRNAs and affects autophagy of OC cells

**Fig. 3:**
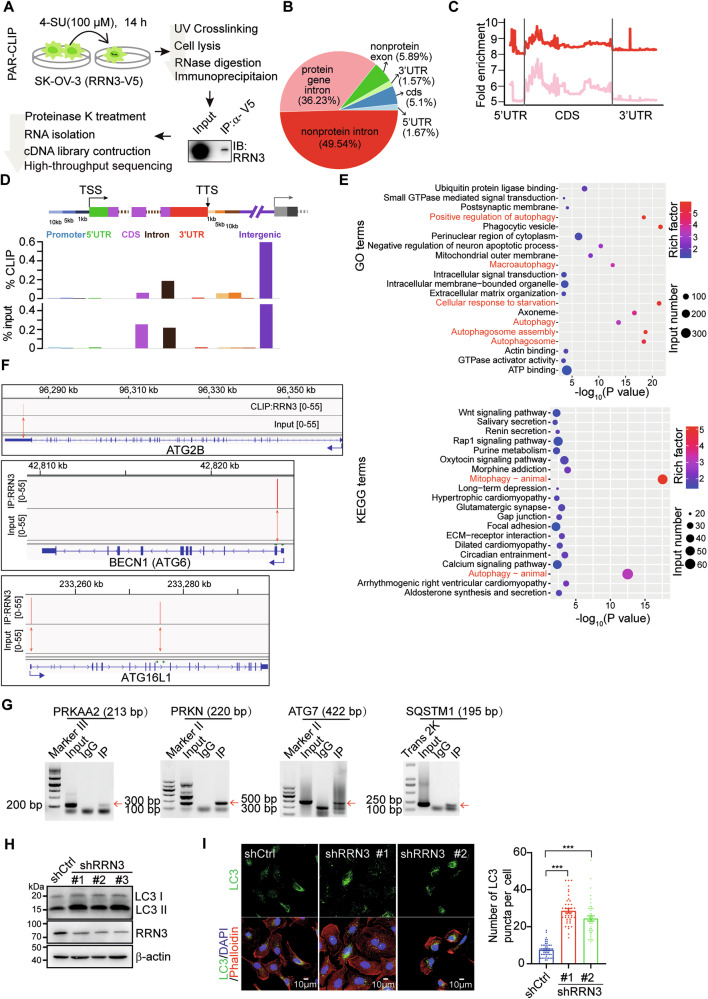
RRN3 interacts with autophagy-related mRNAs and affects autophagy. **A** The schematic diagram of PAR-CLIP method for the identification of RRN3-bound RNAs. **B** The statistics of RRN3-bound RNA species and the binding locations. **C** Metagene analysis of RRN3-binding sites along the different regions of mRNAs. **D** Bar graph showing the distribution of the RRN3-binding sites in different genomic structures. **E** Gene ontology- and KEGG pathway-enrichment analyses of RRN3-bound mRNAs. **F** Examples of RRN3-binding sites on autophagy-related mRNAs identified by PAR-CLIP. **G** The verification of the RRN3 binding on autophagy-related mRNAs by RT-PCR analyses of PAR-CLIP samples. **H**, **I** Analyses of autophagy levels of SK-OV-3 cells stably transfected with shRRN3 or control shLacZ and pre-treated with 10 μM CQ for 4 h by western blot (**H**) or IF (**I**). The autophagy level was quantified by counting the LC3 puncta numbers in each cell. Data are shown as means ± SEM (***p < 0.001 by unpaired Student’s t-test).

To identify RRN3 directly regulated mRNAs, we performed photoactivatable-ribonucleoside-enhanced cross-linking and immunoprecipitation (PAR-CLIP) to capture mRNAs bound by RRN3-engaged RNA-processing machinery (Fig. 3A). High-throughput sequencing identified a broad range of genes whose transcripts were potentially bound by RRN3 (Supplementary Fig. 3A and B, Supplementary File 5), among which 55.43% were non-coding RNA and 43.57% were protein-coding mRNAs (Fig. 3B). Metagene analysis also showed obvious RRN3-binding sites peaked at the 3’ UTR region of protein-coding genes (Fig. 3C). In addition, obvious RRN3-binding sites were also found at the downstream region of TTS sites (Fig. 3D), supporting the possible involvement of RRN3 in the regulation of transcriptional termination. Gene ontology and signaling pathway analyses suggested that these RRN3-regulated genes were mainly involved in the cellular responses to starvation and the whole process of autophagy pathway (Fig. 3E and Supplementary Fig. 3C). Across these autophagy-related genes, RRN3 binding sites dispersed from 5’ UTR to 3’ UTR regions and some autophagy-related genes were validated. (Fig. 3F, G, Supplementary Fig. 3D, E). In cultured SK-OV-3 cells, knocking down RRN3 significantly enhanced the autophagy under normal condition (Fig. 3H, I, Supplementary Fig. 3F) and rapamycin-induced autophagy (Supplementary Fig. 3G).

### Nutrient deficiency and the mimicking rapamycin treatment divert RRN3 out of nucleolus by phosphorylation at S199

**Fig. 4:**
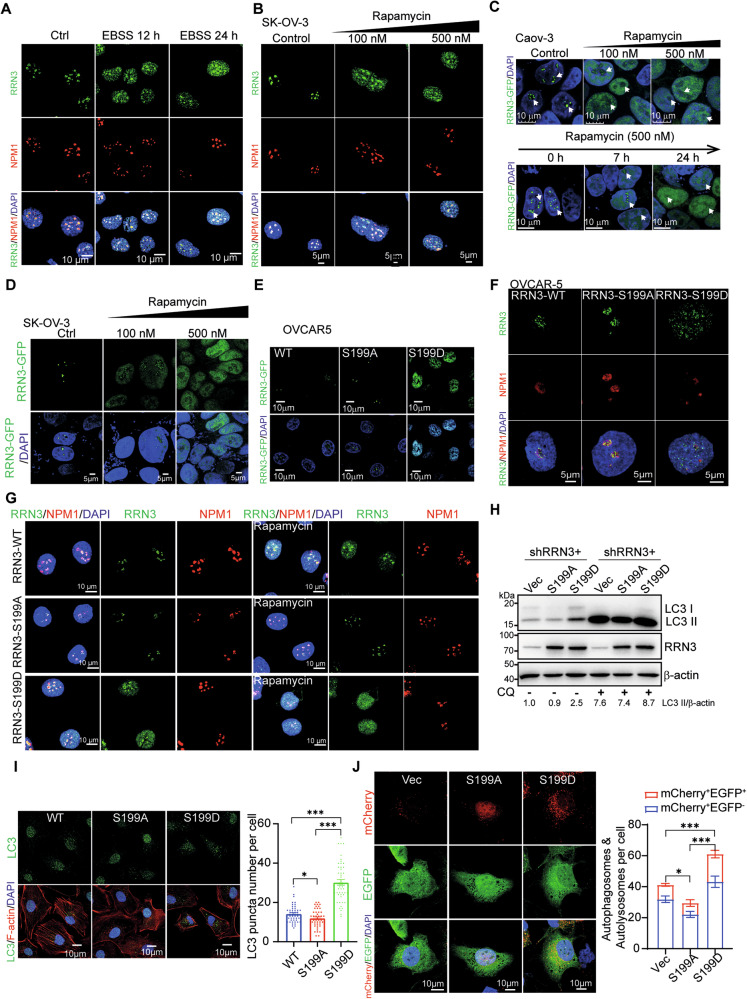
Nutrient stress diverts RRN3 out of nucleolus by phosphorylation at S199 residue. Representative immunofluorescent images of the subcellular localization of endogenous RRN3 in SK-OV-3 cells cultured in complete medium (control, Ctrl) or nutrient-depleted EBSS buffer for indicated periods (**A**) or treated with Rapamycin or DMSO (Ctrl) for 24 h (**B**). The nuclei and nucleolus were stained by DAPI and NPM1 antibody, respectively. GFP-fused RRN3 (RRN3-GFP) were transfected into Caov-3 (**C**) or SK-OV-3 (**D**) cells to trace the subcellular localization of RRN3 upon the treatment of Rapamycin at indicated dosages and time courses. **E** Representative images of OVCAR-5 cells expressing GFP-fused wildtype (WT) or S199-mutated (S199A and S199D). Representative images of IF staining to trace the V5-tagged WT or S199-mutated RRN3 in OVCAR-5 cells (**F**) and in SK-OV-3 cells treated with Rapamycin or DMSO (**G**). The autophagy levels of SK-OV-3 cells, whose endogenous RRN3 was substituted with S199-mutated RRN3, an empty vector or wild-type (WT) RRN3, were treated with or without 10 μM CQ for 4 h before further analyses by western blot (**H**) or by IF staining of LC3 (**I**, left panel). The numbers of LC3 puncta in each cell were counted (means ± SEM, ***p < 0.001, *p < 0.05 by unpaired Student’s t-test) (**I**, right bar graph). **J** SK-OV-3 cells harboring mCherry-EGFP-LC3 reporter were stably transfected with indicated vectors and examined by fluorescent microscope (left panel). The bar graph (right panel) shows the quantification of autophagosomes (mCherry^+^ EGFP^+^) and autolysosomes ^(^mCherry^+^ EGFP^−^) in each cell ^(^means ^± ^SEM, ***p < 0.001, *p < 0.05 by unpaired Student’s t-test).

Due to its high energy-consuming characteristic, ribosomal RNA (rRNA) synthesis is a tightly regulated process to support cell survival under the environmental stress including nutrient deficiency, in which the post-translational modification of the initiation factor RRN3 is a key target [5] (Supplementary Fig. 4A). And several studies have shown that mTOR inhibitor rapamycin, which induces cellular responses to starvation including autophagy, is able to cause the phosphorylation of the serine 199 residue (S199) of RRN3, which is conserved from yeast to human (Supplementary Fig. 4B), and hence the translocation of RRN3 out of the nucleolus [8]. Kinase prediction based on the substrate motif suggested that AMPK, a key kinase regulating cellular responses to starvation, was among the top-ranked kinase for S199 (Supplementary Fig. 4C). To validate this prediction, a specific antibody against S199-phosphorylated RRN3 (p-RRN3-S199) was customized and its specificity was confirmed by immunofluorescence and immunoprecipitation assays in SK-OV-3 cells (Supplementary Fig. 4D–G). Treatment with AMPK activator AICAR induced a marked increase in phosphorylation at the RRN3 S199 site, confirming that this residue is a phosphorylation target downstream of AMPK signaling (Supplementary Fig. 4H). And the S199 (corresponding to S186 in yeast RRN3) locates in the interface of RRN3 and RPA43, the major component of Pol I that recruits RRN3 (Supplementary Fig. 5A, B), suggesting the modification on S199 has the potential to cause the release of RRN3 from Pol I. In amino acids-deficient medium, we observed increasing amount of RRN3 proteins diffused out of the nucleolar into the nuclear plasma within 24 hours by immunofluorescent staining of endogenous RRN3 in OC cells (Fig. 4A, Supplementary Fig. 5C). This translocation was able to be mimicked by the treatment of mTOR inhibitor rapamycin in a dose-dependent manner (Fig. 4B, Supplementary Fig. 5D, E), while the expression level of RRN3 was not affected (Supplementary Fig. 5F). Rapamycin treatment also triggered the translocation of EGFP-fused RRN3 out of nucleolar in OC cells (Fig. 4C, D). Western blot analysis showed that rapamycin treatment at the indicated concentration and duration markedly reduced the phosphorylation of S6K1 and 4EBP1, confirming effective inhibition of mTOR signaling (Supplementary Fig. 5G). Co-treatment with rapamycin and the AMPK inhibitor Dorsomorphin 2HCl did not affect the subcellular localization of RRN3 or the phosphorylation at Ser199 (p-RRN3-S199) (Supplementary Fig. 5H, I). Furthermore, the nuclear plasma-localization of S199 phosphorylation-mimicry mutant S199D and the nucleolar localization of its phosphorylation-deficient mimicry mutant S199A suggested that phosphorylation on S199 was sufficient for the translocation of RRN3 out of nucleolus (Fig. 4E, F), while rapamycin treatment could no longer cause the translocation of RRN3 harboring S199A mutation (Fig. 4G). Consistently, autophagy was significantly induced only in OC cells expressing phosphorylation-mimicry S199D mutant of RRN3 (RRN3^S199D^), while caused much less autophagy in cells whose endogenous RRN3 was silenced or substituted with S199A mutant (Fig. 4H, I, Supplementary Fig. 5J). Autophagy flux assay suggested that RRN3^S199D^ affects autophagy induction by causing an increase in the punctate number of both mCherry^+^ EGFP^+^ and mCherry^+^ EGFP^−^(Fig. 4J).

### S199-phosporylated RRN3 increases the usage of distal APA of OPTN to increase mRNA stability and protein production

**Fig. 5:**
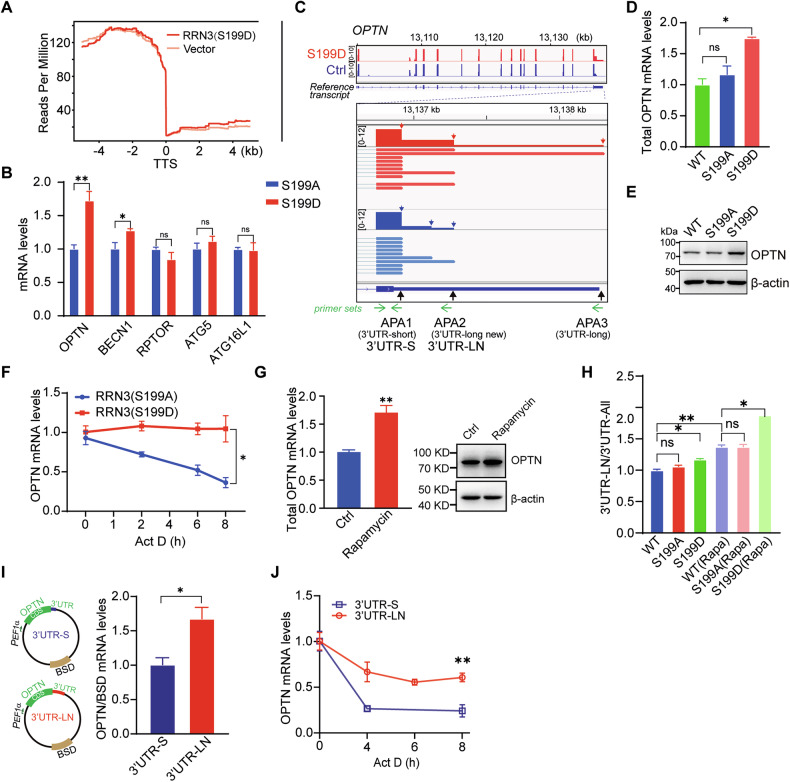
Phosphorylation of RRN3 at S199 increases the usage of distal APA of OPTN to increase mRNA stability and protein production. **A** Metagene analysis of the autophagy-related genes across the transcription termination sites (TTS) based on Long-read RNA sequencing. **B** RT-qPCR analysis of the mRNA levels of OPTN, BECN1, RPTOR, ATG5, and ATG16L1 in indicated SK-OV-3 cells. **C** Long-read RNA sequencing results showing the transcript variants of OPTN in SK-OV-3 cells, whose RRN3 was substituted with RRN3^S199D^ or empty vector as a control. **D**, **E** The mRNA and protein levels of OPTN in indicated SK-OV-3 cells were analyzed by RT-qPCR (**D**, means ± SEM, *p < 0.05, ns, not significant, by unpaired Student’s t-test) and western blot (**E**). **F** The mRNA stability of OPTN was measured by RT-qPCR at the indicated time points post treatment by 10 μg/mL actinomycin D (Act D) in SK-OV-3 cells expressing RRN3^S199A^ or RRN3^S199D^ (means ± SEM, *p < 0.05, by unpaired Student’s t-test). **G** The total mRNA and protein levels of OPTN in SK-OV-3 cells treated with or without 500 nM Rapamycin for 24 h analyzed by RT-qPCR (left bar graph, means ± SEM, **p < 0.01, by unpaired Student’s t-test) and western blot (right panel). **H** RT-qPCR analysis of the ratio between amplicons (3’ UTR-LN/ 3’ UTR-All) in indicated SK-OV-3 cells treated with or without Rapamycin (500 nM, 24 h) (means ± SEM, **p < 0.01, *p < 0.05, ns, not significant, by unpaired Student’s t-test). The construction of plasmids inserted with OPTN coding sequence followed by short or newly identified long 3’ UTR (3’UTR-S or 3’UTR-LN) were transfected into SK-OV-3 cells (**I**, left panel), which were subjected to RT-qPCR analyses of the steady state levels (**I**, right bar graph) and stabilities (**J**) of ectopically expressed OPTN transcripts and the mRNA stability. Data are shown as means ± SEM, **p < 0.01, *p < 0.05 by unpaired Student’s t-test.

To investigate how RRN3 regulated the APA of autophagy-related mRNAs under cellular starvation circumstances, we performed long-read RNA sequencing analyses on SK-OV-3 cells whose RRN3 was substituted with RRN3^S199D^ or empty vector as a control (Supplementary Fig. 6A, Supplementary File 6). We observed no significant changes on the rates of alternative splicing (Supplementary Fig. 6A) and a very slightly increased number of genes with single polyadenylation site and slightly decreased proportion of genes with multiple APA sites (Supplementary Fig. 6B). In case of RRN3-bound autophagy-related mRNAs, we observed that RRN3^S199D^ did cause the difference in the usage of APAs (Fig.5C, Supplementary Fig. 6C). According to metagene analysis, autophagy-related genes downstream of the transcription termination site had higher reads in RRN3^S199D^(Fig. 5A), suggesting that RRN3^S199D^ may stabilize autophagy-related gene transcripts by alternative polyadenylation.

We conducted an analysis by integrating the number and changes of APA sites in autophagy-related genes, along with the results from PAR-CLIP (Supplementary Fig. 3C). The following five genes were identified: OPTN, BECN1, RPTOR, ATG5, and ATG16 L1. Among them, compared to RRN3^S199A^, the mRNA expression levels of OPTN and BECN1 were elevated in RRN3^S199D^ (Fig. 5B, Supplementary Fig. 6D, L). OPTN exhibited a more substantial increase in expression levels, along with a more marked alteration in APA (Fig. 5B, C). We next selected a key autophagy regulator OPTN as an example, for which RRN3^S199D^ caused the shift of APA usage to the distal sites and therefore resulted in the mRNA variants with long 3’ UTR (3’UTR-L and 3’UTR-LN, N denotes newly identified in this study) and short 3’ UTR (3’UTR-S) (Fig. 5C), to investigate the role of RRN3 in the regulation of APA. Rapamycin treatment-induced increase of OPTN mRNA levels, among which the 3’UTR-LN mRNA showed bigger increase (Fig. 5G, H, Supplementary Fig. 6G) and hence increased OPTN proteins (Fig. 5G). This phenomenon was able to be mimicked by the introduction of RRN3^S199D^, but not RRN3^S199A^ (Fig. 5D, E, Supplementary Fig. 6D, E), which increased the usage of distal APA and therefore increased production of 3’UTR-LN mRNA (Fig. 5H, Supplementary Fig. 6G).Consistently, treatment of cells with the AMPK activator AICAR also led to an increase in 3’UTR-LN mRNA levels, whereas co-treatment with rapamycin and the AMPK inhibitor Dorsomorphin 2HCl abolished this effect, suggesting that AMPK activity is required for the rapamycin-induced upregulation of 3’UTR-LN (Supplementary Fig. 6H). The mRNA stability assay showed that RRN3^S199D^-induced OPTN mRNA had longer half-lives (Fig. 5F, Supplementary Fig. 6F). We further construct plasmids with coding sequencing of OPTN followed by 3’UTR-S or 3’UTR-LN sequences and transfected SK-OV-3 cells (Fig. 5I). Consistently, the mRNA with 3’UTR-LN showed higher expression level and longer half-life (Fig. 5J). Additionally, analysis of other autophagy-related genes revealed that both BECN1 mRNA and protein levels were elevated in RRN3^S199D^-expressing SK-OV-3 and OVCAR-5 cells (Fig.5B, Supplementary Fig. 6E, I, L). Moreover, mRNA stability assays showed that BECN1 mRNA induced by RRN3^S199D^ had an extended half-life, indicating enhanced transcript stability (Supplementary Fig. 6K, N). Consistently. APA analysis of BECN1 also revealed significant differences between the RRN3^S199D^ group and the controls (RRN3^WT^ and RRN3^S199A^) (Supplementary Fig. 6J, M). These results suggested that nutrient deficiency triggered phosphorylation of RRN3 on S199 diverted RRN3 to the nucleoplasm, where it regulated the APA of autophagy-related mRNAs to enhance their stability and protein production. Although RRN3^S199D^ expression induced alternative polyadenylation (APA) changes in several autophagy-related genes, a subset—including RPTOR, ATG16L1, and ATG5—showed no detectable changes in either mRNA or protein levels. (Fig.5B, Supplementary Fig. 6O, P). These results implied that other post-transcriptional regulation mechanisms, such as miRNA targeting or subcellular localization.

### PABPC1 is involved in RRN3-regulated APA

**Fig. 6:**
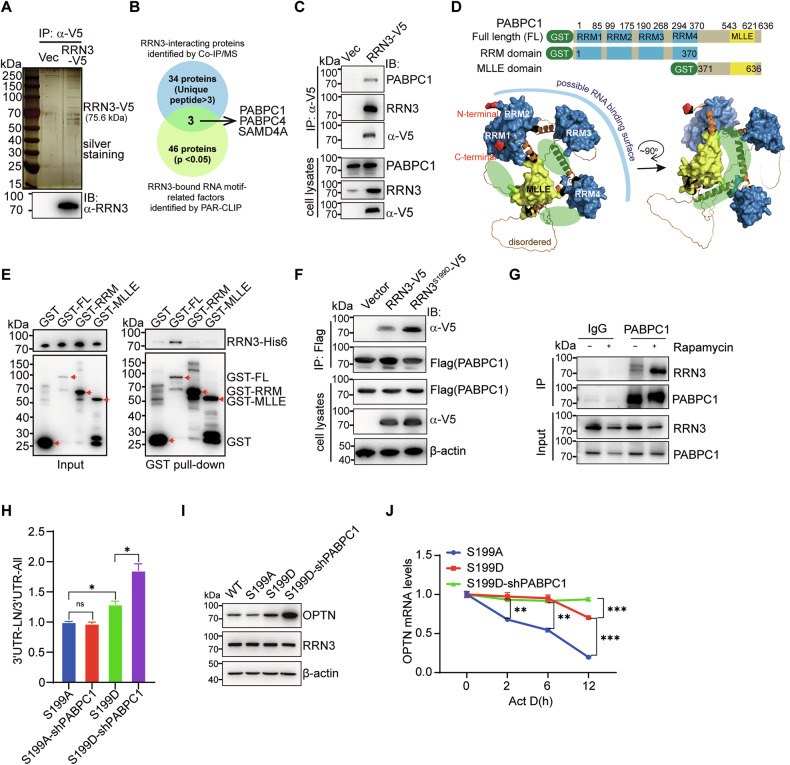
PABPC1 is involved in RRN3-regulated APA. The V5-antibody-immunoprecipitated proteins from SK-OV-3 cells expressing V5-tagged RRN3 were resolved by SDS-PAGE and shown by silver staining (**A**, up panel) and western blot (**A**, bottom panel), or subjected to mass spectrometry (MS) identification of RRN3-interacting proteome (**B**), which was compared with predicted factors that recognize RRN3-bound RNA motifs identified by PAR-CLIP. **C** The interaction between RRN3 and PABPC1 was examined in SK-OV-3 cells by overexpressing V5-tagged RRN3 and performing immunoprecipitation assays using a V5 antibody. **D** The schematic diagram showing the design of GST-fused constructs containing the full length (FL) or different domains of PABPC1 (up panel). The three-dimensional structure of PABPC1 is predicted by AlphaFold2 (#AF-P11940-F1-v4) (bottom panel). **E** Western blot analysis of His6-tagged RRN3 protein pulled down by indicated GST-PABPC1 fusion proteins. **F** Transient transfection of V5-tagged wild type (WT) or S199D-mutated RRN3 together with Flag-tagged PABPC1 in HEK 293T cells for immunoprecipitation assay using anti-Flag antibody. **G** SK-OV-3 cells treated with Rapamycin (500 nM, 24 h) or DMSO control were subjected to immunoprecipitation assay using anti-PABPC1 antibody. **H** RT-qPCR analysis of the ratio between amplicons (3’ UTR-LN/ 3’ UTR-All) in indicated SK-OV-3 cells.Data are shown as means ± SEM, *p < 0.05, ns, not significant, by unpaired Student’s t-test. **I** The protein levels of OPTN in indicated SK-OV-3 cells were analyzed by western blot. **J** The mRNA stability of OPTN was measured by RT-qPCR at the indicated time points post treatment by 10 μg/mL actinomycin D (Act D) in SK-OV-3 cells expressing RRN3^S199A^, RRN3^S199D^ or RRN3^S199D-shPABPC1^(means ± SEM,***p < 0.001, **p < 0.01, by unpaired Student’s t-test).

To get insights into how RRN3 regulates APA, we identified RRN3-interacting proteins in SK-OV-3 cells by coimmunoprecipitation and mass spectrometry (Fig. 6A, Supplementary File 7). Among the top 34 candidates, three proteins, i.e., PABPC1, PABPC4 and SAMD4A, were also found in the predicted factors that were able to recognize the RRN3-bound RNA motifs identified by PAR-CLIP (Fig. 6B, Supplementary File 8). Previous studies have reported that PABPC1 is involved in the regulation of alternative polyadenylation and mRNA stability [29, 30]. Further western blot analysis confirmed the presence of PABPC1 in the immunoprecipitated proteins (Fig. 6C). Next, we performed a GST pull-down assay to map the RRN3-interacting domains in PABPC1. As shown in Fig. 6D, E, RRN3 showed the strongest affinity only with the full-length PABPC1, but not with the dissected N-terminal RNA-binding RRM domain or the C-terminal MLLE-containing domain, suggesting the RRN3-binding sites may locate in the interface of RRM domain and MLLE domain. In addition, the RRN3^S199D^ enhances the interaction with PABPC1 and promotes its nucleoplasmic accumulation (Fig. 6F, Supplementary Fig. 7A), similar to the effect of Rapamycin (Fig. 6G, Supplementary Fig. 7C, D). The interaction between RRN3 and PABPC1 in the nuclear plasma of SK-OV-3 cells expressing the S199D mutant of RRN3 was further visualized by confocal microscopy (Supplementary Fig. 7B), which could be mimicked by rapamycin treatment (Supplementary Fig. 7E). And knocking down PABPC1 further increased the level of OPTN mRNA with 3’UTR-LN and consequently elevated total OPTN protein in RRN3^S199D^-substituted SK-OV-3 cells (Fig. 6H, I), but not in RRN3^S199A^-substituted cells (Fig. 6H, I), by enhancing the mRNA stability of OPTN (Fig. 6J). These results suggest the involvement of PABPC1 in RRN3-regulated APA of autophagy-related mRNAs. But it remains elusive about how RRN3 affects PABPC1-regulated APA.

### RRN3-regulated autophagy is essential for OC progression

**Fig. 7:**
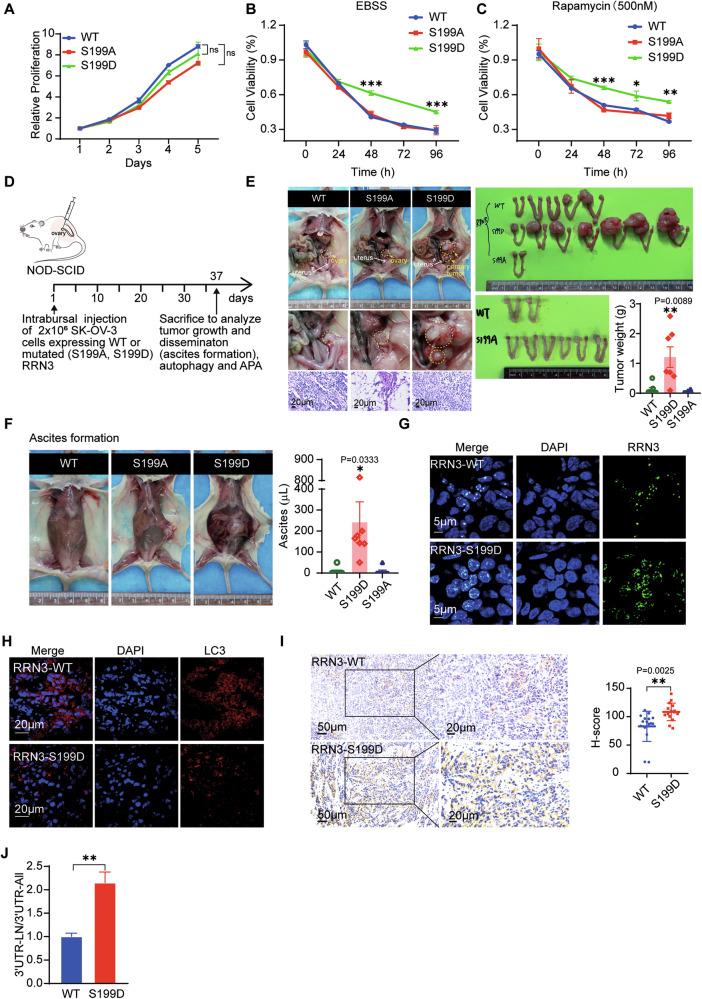
RRN3-regulated autophagy is essential for OC progression. **A** The proliferation of SK-OV-3 cells harboring indicated RRN3 was assessed by CCK-8 assay. The viability of indicated SK-OV-3 cells cultured in nutrient-deficient EBSS buffer (**B**) or treated with Rapamycin (500 nM) (**C**) was analyzed by CCK-8 assay. (means ± SEM, ***p < 0.001, **p < 0.01, *p < 0.05, ns, not significant, by unpaired Student’s t-test). **D** Schematic of the orthotopic model of ovarian cancer. SK-OV-3 cells expressing RRN3^WT^ or mutated RRN3 (RRN3^S199A^, RRN3^S199D^) were injected into the bursal of ovary of NOD-SCID mice (n = 7). **E** Representative images of primary OC tumors in situ which were verified by H&E staining (left panels). The tumor xenografts were dissected (middle panels) for the measurement of tumor weight (right bar graph). Data are shown as means ± SEM (n = 7), **p < 0.01, by unpaired Student’s t-test. **F** The representative images showing the ascites formation (left panels) and the quantification (right bar graph)(*p < 0.05, by unpaired Student’s t-test). Representative immunofluorescent images of the tumor sections stained with DAPI and anti-RRN3 antibody (**G**) or anti-LC3 antibody (**H**). **I** Immunohistochemical staining (left panels) and quantification (right bar graph) of OPTN in the tumor xenograft sections. Data are shown as means ± SEM, **p < 0.01, by unpaired Student’s t-test. **J** RT-qPCR analysis of the ratio between amplicons (3’ UTR-LN/ 3’ UTR-All) of OPTN in the tumor xenografts (means ± SEM, **p < 0.01, by unpaired Student’s t-test).

As a central mechanism to maintain cellular homeostasis, autophagy plays important roles during the whole progression of tumors. Although the proliferation of SK-OV-3 cells and OVCAR-5 cells cultured in complete medium was not affected by S199A or S199D mutation on RRN3 (Fig. 7A, Supplementary Fig. 8A), the cells with RRN3^S199D^ showed higher viability than that with RRN3^WT^ or RRN3^S199A^ when cultured in nutrient-deficient EBSS buffer or mimicry rapamycin treatment (Fig. 7B, C, Supplementary Fig. 8B, C).

**Fig. 8:**
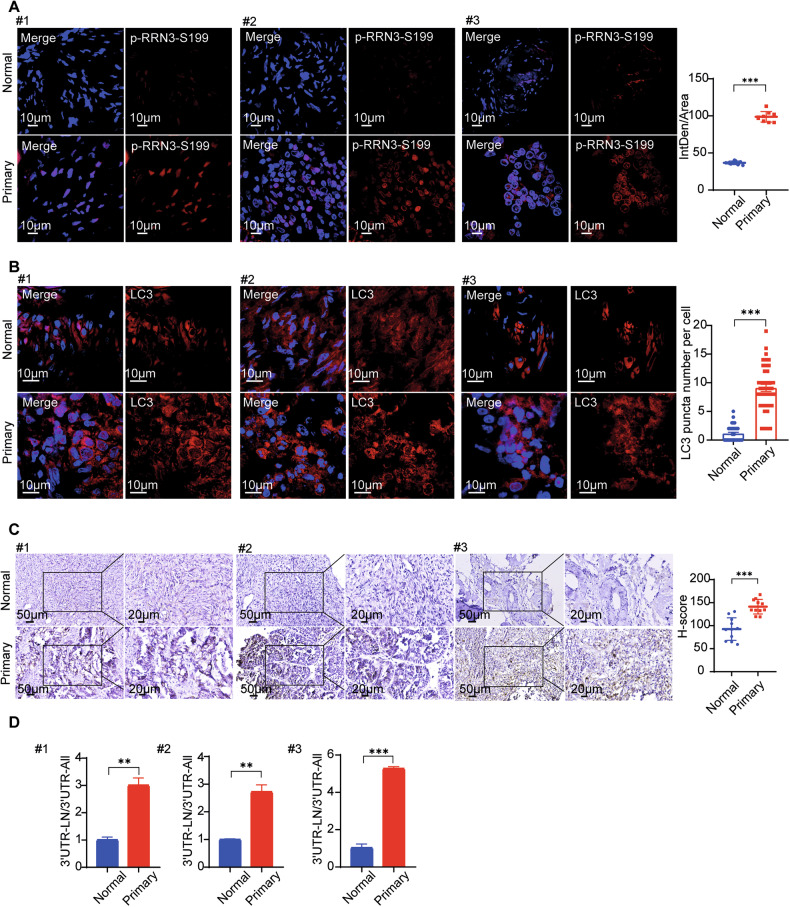
Phosphorylation of RRN3 at serine 199 enhances autophagy contributing to ovarian cancer progression. **A** Representative images of immunofluorescence staining for Phosphorylation of RRN3 at serine 199 (p-RRN3-S199) in three pairs of normal and primary ovarian cancer tissues. Nuclei were stained with DAPI(blue). The right panel shows the quantification of fluorescence intensity. Data are shown as means ± SEM, ***p < 0.001 by unpaired Student’s t-test. **B** Representative immunofluorescence images showing LC3 expression in three pairs of normal and primary ovarian cancer tissues. Nuclei were stained with DAPI(blue). The numbers of LC3 puncta (right bar graph) were counted(means ± SEM, ***p < 0.001 by unpaired Student’s t-test). **C** Immunohistochemical staining (left panels) and quantification (right bar graph) of OPTN in three pairs of normal and primary ovarian cancer tissues. Data are shown as means ± SEM, ***p < 0.001 by unpaired Student’s t-test. **D** RT-qPCR analysis of the ratio between amplicons (3’ UTR-LN/ 3’ UTR-All) of OPTN in three pairs of normal and primary ovarian cancer tissues (means ± SEM, **p < 0.01,***p < 0.001 by unpaired Student’s t-test).

To further investigate the impact of RRN3-regulated autophagy on OC progression in vivo, we performed intrabursal injection of SK-OV-3 cells and OVCAR-5 cells, in which the endogenous RRN3 was substituted with S199A, S199D or wild type (WT) RRN3 as a control, into the mouse ovary. We observed that OC xenograft with RRN3^S199D^ showed much faster growth than that with RRN3^WT^, while RRN3^S199A^-expressing cells formed neglectable tumor xenograft at indicated time period (Fig. 7D, E, Supplementary Fig. 8D–F). RRN3^S199D^-expressing SK-OV-3 cells also caused more malignant ascites formation (Fig. 7F). The subcellular localization of RRN3 in the tumor xenografts were verified by IF staining (Fig. 7G), which confirmed that the nuclear plasma-localized RRN3^S199D^ induced a higher level of autophagy, as evidenced by both LC3 IF and OPTN IHC analyses (Fig. 7H, I, Supplementary Fig. 8G, H). Additionally, an increase in OPTN (3’ UTR-LN) mRNA was observed (Fig. 7J, Supplementary Fig. 8I). Paired normal and primary ovarian cancer tissue specimens were collected from patients. Compared to normal tissues, primary ovarian cancer tissues showed a significant increase in p-RRN3-S199 expression and LC3 puncta formation, as revealed by immunofluorescence staining (Fig. 8A, B). Consistently, immunohistochemical analysis demonstrated a marked upregulation of OPTN in primary ovarian cancer tissues (Fig. 8C). Moreover, qRT-PCR analysis indicated a significant elevation of OPTN mRNA containing the 3’UTR-LN in primary ovarian cancer samples **(**Fig. 8D**)**. Collectively, these results suggest that RRN3-regulated alternative polyadenylation (APA) of autophagy-related genes, mediated by nutrient deficiency-induced phosphorylation at S199, plays an essential role in ovarian cancer progression.

## Discussion

Under nutritional stress, mammalian cells regulate their proteome to maintain cellular homeostasis by either inhibiting translation or engaging in degradation processes mediated by proteasomes and autophagy systems [31, 32]. Protein translation is an energy-intensive process in all living cells that synthesizes proteins from amino acids using messenger RNA (mRNA). In tumor cells, aberrant oncogenic signaling pathways, including the PI3K-AKT-mTOR, RAS-MAPK, and MYC pathways, alongside microenvironmental factors such as hypoxia and nutrient availability, drive cellular remodel the translational landscape through the regulation of mRNA translation to drive tumour formation and malignancy [33]. The reduction of protein synthesis mainly refers to the inhibition of translation initiation (eukaryotic initiation factor 4 F (eIF4F) complex and eIF2) and elongation. GCN2 acts as a critical sensor of amino acid availability and mediates a protective response by repressing global translation through eIF2α phosphorylation [34]. The ribosome is the organelle responsible for translation. Cells degrade ribosomal proteins through non-autophagic proteasome pathways and significantly inhibit the translation of ribosomal proteins to control ribosome levels [31]. However, the mechanism of ribosome level control is not fully understood. Our research found that during cellular stress, cells regulate ribosome levels through rRNA transcription, which accounts for about 60% of the total RNA in cells [3, 35] and consumes the majority of energy. Under stress conditions (hypoxia and nutrient deprivation), the reduction in protein synthesis is due to the inhibition of mTORC1, which in turn hinders the phosphorylation of eIF4E-binding proteins and the formation of the eIF4F complex. We found that mTORC1 inhibition not only suppresses translation but also regulates the localization of the RNA polymerase I transcription factor RRN3 to directly modulate rRNA levels. Directly controlling the ribosome level from the rRNA transcription level is also an energy-saving approach.

RRN3 functions as a transcription factor for RNA polymerase I, however, its multiple phosphorylation sites related to stress-responsive kinases indicate a potential novel role as a regulator of cellular stress. Autophagy is a cellular degradation process that helps maintain cellular homeostasis by removing damaged organelles and proteins. It plays a vital role in various cellular functions, including responses to stress, nutrient availability, and the regulation of cellular metabolism. Similarly, when cells are under stress, the co-transcriptional process that cells use to adapt to environmental changes will also be disrupted, causing intron retention to prevent 3’ end cleavage and promote transcriptional readthrough [36]. No studies have documented the regulatory role of RRN3 in autophagy, especially transcriptional regulation. Our research shows that RRN3 undergoes a localization change (nucleolus translocation to the nucleoplasm) under nutritional stress conditions to regulate the transcription of autophagy-related genes. PAR-CLIP experiments demonstrated that RRN3 interacts with multiple RNAs associated with autophagy and stress signaling pathways (Fig. S3C). In our work, we chose the autophagy receptor OPTN, which undergoes changes in alternative polyadenylation, as the research subject. Since APA can alter 3’UTR length and hence the potential miRNA interaction, we used miRDB to predict miRNAs targeting the distinct OPTN isoforms (3’UTR-S and 3’UTR-LN). The predictions were based on the sequence differences of the 3’UTRs between the two isoforms. Several miRNAs, including hsa-miR-504-3p, hsa-miR-6742-3p, hsa-miR-6783-3p, hsa-miR-1343-3p, hsa-miR-6879-3p, and hsa-miR-122-5p, were uniquely associated with specific isoforms [37]. However, whether RRN3-caused increase in OPTN mRNA stability and protein level is through the miRNA-involved mechanism or other RNA-binding protein factors remains elusive.

## Materials and methods

The key resources table are listed in Supplementary material.

### Cell culture

SK-OV-3 and Caov-3 were obtained from ATCC (the American Type Culture Collection). ID8 cells were obtained from Merck (Darmstadt, Germany). HEK 293 T cells for the preparation of lentivirus were obtained from Thermo-Fisher Scientific Inc. OVCAR-5 cells were originally got from the Fox Chase Cancer Center (Philadelphia, USA) and subsequently maintained in our laboratory. All cell lines were authenticated by STR profiling (Shanghai Zhong Qiao Xin Zhou Biotechnology Co., Ltd) and cultured in the recommended conditions and medium, i.e., McCoy’s 5a (Biological Industries (BI), Kibbutz Beit Haemek 25115, Israel) modified medium supplemented with 10% (v/v) fetal bovine serum (FBS) (BI) for SK-OV-3 cells, DMEM (BI) medium supplemented with 10% FBS for Caov-3 and HEK 293 T cells. RPMI 1640 (BI) supplemented with 10% FBS and 0.25 units/mL insulin for OVCAR-5 cells.

### Establishment of stable cell lines

The lentivirus-based RNAi vector system pLV-H1-EF1α-puro (BiOSETTIA, San Diego, CA, USA) was employed to generate stable RRN3-silenced cells. The sequences of shRNA-encoding templates targeting human RRN3 are as follows. shRRN3#1: 5’ AAA AGG ATG TCT GCT ATG TAG ATG GTT GGA TCC AAC CAT CTA CAT AGC AGA CAT CC 3’), shRRN3#2: 5’ AAA AGT AGA TGG TAA GGT TGA TAA CTT GGA TCC AAG TTA TCA ACC TTA CCA TCT AC 3’), shRRN3#3: 5’ AAA AGG ATC ACA CCA AGC TCC TTT GTT GGA TCC AAC AAA GGA GCT TGG TGT GAT CC 3’ (the underlined denote the target sequences of shRNAs).

The cDNAs of RRN3^WT^, RRN3^S199A^, RRN3^S199D^ and RRN3 fused with GFP were amplified by PCR and cloned into the lentivirus-based vector pLV-EF1a-MCS-IRES-Bsd (BiOSETTIA). The entire coding sequence of each clone was validated by sequencing. The transient transfection using Lipofectamine 2000 (Thermo-Fisher Scientific), lentivirus packaging and infection were performed as we previously reported [35].

### Generation of p-RRN3-S199 antibody

A phospho-specific antibody against RRN3 phosphorylated at serine 199 was custom-generated by ABclonal (Wuhan, China). A synthetic phosphopeptide corresponding to residues 194-204 of human RRN3, with the sequence ARYVP(pS)TPWFL-C, was used as the immunogen for antibody production, affinity purification and detection. A non-phosphorylated control peptide (ARYVPSTPWFL-C) was also synthesized for use in affinity purification and assay validation.

### Genome-wide CRISPR/Cas9 knockout screening

The GeCKO (v2.0) CRISPR library developed by Zhangfeng’s lab was obtained from Addgene and amplified using the recommended protocol. The library encompasses 122,411 sgRNAs targeting 19,050 protein-encoding genes, 1864 miRNA and 1000 non-targeting control sgRNAs. A dual-fluorescence transcriptional readthrough (TRT) reporter system (Fig. 1A) was established using the pLV-EF1a-MCS-IRES-Bsd vector to generate stable SK-OV-3 cells harboring the TRT reporter sequence (SK-OV-3^TRT^). In this system, the majority of the encoding sequences of COMMD3 and BMI1 were replaced by the mCherry- and EGFP-encoding sequences, respectively. The sequence in-between, including the terminal part of exon 7, intron 7, exon 8 of COMMD3, and the exon 1, intron 1, initial part of exon 2 of BMI1, was maintained in-frame.

Lentiviruses carrying the library were prepared according to the manufacturer’s protocol and used to infect the SK-OV-3^TRT^ cells. The viral titer was estimated and the transduction was performed at a low multiplicity of infection (MOI) ~0.3. Following puromycin (2 μg/mL) selection, mCherry^+^ EGFP^-^ cells were sorted via flow cytometry. Genomic DNA was extracted from the sorted cells (mCherry^+^ EGFP^-^) and the parental cells (unsorted cells). The sgRNA-encoding sequences were amplified by PCR, and high-throughput DNA sequencing was performed on an Illumina HiSeq platform as depicted in the Fig. 1.

### ChIP-seq

To profile the coverage of RNA polymerase II (Pol II), chromatin immunoprecipitations (ChIP) were conducted in RRN3-silenced (shRRN3) or control (shLacZ, BiOSETTIA) SK-OV-3 cells. An antibody against Pol II (Active Motif, Catalog No. 91151, San Diego, CA, USA) was used, and the ChIP-IT Express Kit (Active Motif) was employed following the manufacturer’s instructions. The immunoprecipitated and input DNA fragments were further analyzed via high throughput DNA sequencing on BGISEQ-500 platform (BGI, Shenzhen, China).

### Photoactivatable-ribonucleoside-enhanced CLIP (PAR-CLIP)

A V5-tag-encoding sequence was inserted into RRN3 cDNA right after the start codon and then cloned into the pLV-EF1a-MCS-IRES-Bsd vector. This vector was used to generate stable SK-OV-3 cells that expressed N-terminal V5-tagged RRN3. PAR-CLIP was performed as described in [38] with slight modifications. Briefly, V5-RRN3-expressing cells were cultured in complete medium supplemented with 100 μM 4-thiouridine (4-SU) for 14 h prior to cross-linking. After washing the cells with ice-cold phosphate-buffered saline (PBS), the cells were cross-linked by exposure to 365-nm UV light to receive a total energy of 0.2 J/cm^2^. The cells were then suspended in 3 volumes of NP40 lysis buffer [50 mM HEPES-KOH, pH 7.5, 150 mM KCl, 2 mM EDTA, 1 mM NaF, 0.5% (v/v) Nonidet P-40 (NP-40), 0.5 mM DTT, protease inhibitor cocktail (Roche, Swiss)] and incubate on ice for 10 min. The cell lysates were then treated with 10 μg/mL RNase A and 4 U/mL DNase I (Thermo-Fisher Scientific). Ten percent of cleared cell lysates were used as the input and the rest were subjected to immunoprecipitation using an anti-V5 antibody (Thermo-Fisher Scientific). This was followed by elution with a V5 peptide (MedChemExpress, Shanghai, China). The eluted samples were digested with proteinase K, and then the RNA fragments were extracted using a 25:24:2 (v/v/v) phenol/chloroform/isoamyl alcohol mixture(pH<5.2). The purified RNA samples were then subjected to the library construction and deep sequencing on the Illumina Novaseq 6000 platform (SEQHEALTH, Wuhan, China). The primer sequences are listed in the Supplementary Tables.

### Long-read RNA sequencing

The total RNA was extracted from RRN3-silenced, RRN3^S199D^, or control SK-OV-3 cells using TRIeasy^TM^ Total RNA Extraction Reagent (TransGen Biotech, Beijing, China). Long-read RNA sequencing was performed on a PacBio Sequel platform. For each sample, the CCS module of the IsoSeq3 program was used to generate circular consensus sequence (CCS) reads from the sub-reads obtained from the sequencing run. The full-length non-chimeric transcript was obtained by using IsoSeq3 software to remove poly A and chimeric sequences from the full-length sequence. Subsequently, clustering and error correction were performed to obtain high-quality isoforms (HQ) and low-quality isoforms (LQ) with an accuracy of over 0.99. GMAP software was used to align the clustered high-quality (HQ) isoforms with the reference genome (hg38). All data were visualized in and exported from IGV for generating genome browser figures.

### Immunoprecipitation and mass spectrometry (IP/MS)

For IP experiments, SK-OV-3 cells stably transfected with empty vector or V5-RRN3-expressing vector were seeded in 15-cm dishes. The chromatin-associated proteins were extracted as previously described [39], from which the RRN3-bound proteins were immunoprecipitated using anti-V5 antibody (Thermo-Fisher Scientific) and identified by UHPLC-MS/MS using an EASY-nLCTM 1200 UHPLC system (Thermo-Fisher Scientific, Germany) coupled with an Q ExactiveTM HF-X mass spectrometer (Thermo-Fisher Scientific, Germany) in Novogene Co., Ltd. (Beijing, China).

### Autophagy analysis

The autophagy flux tracing vector pCDH-CMV-mC-G-LC3B-P was obtained from Addgene (#124974) [40]. Quantitative analysis of autophagy was performed either by flow cytometry using a BD LSR Fortessa or by confocal microscopy using an Olympus FV1000. For the quantification of autophagy by western blot, the cells were pretreated with or without the lysosomal inhibitor Chloroquine (CQ, 10 μM) before being harvested for LC3 analysis.

### GST pull-down assay

RRN3 cDNA was subcloned into the vector pET-20b (+) (Sigma-Aldrich, St. Louis, MO 63178, United States) with a C-terminal 6xHis tag (His6). The cDNA encoding GST-PABPC1 was constructed in the pGEX-6P-1 vector (GE Healthcare Bio-Sciences, Uppsala, Sweden). Both plasmids were transduced into *Escherichia coli* (BL21). The His6-RRN3 protein was purified by using the Ni-NTA Agarose (QIAGEN, CA, USA) according to the manufacturer’s protocol and the GST or GST-PABPC1 proteins were purified using glutathione Sepharose 4B resin (GE Healthcare).

### mRNA stability assay

SK-OV-3 cells were treated with actinomycin D at a final concentration of 10 μg/mL at indicated time points before the total RNAs were extracted with TRIeasy^TM^ Total RNA Extraction Reagent and analyzed with RT-qPCR. GAPDH was used for normalization. The primers are shown in the Supplementary Tables.

### Protein extraction and western blot

Total proteins were extracted from cells using 2 × SDS loading buffer and boiled at 95 °C for 5 min. Samples were sonicated to fragment genomic DNA. After centrifugation at 12,000 × g for 10 min, the supernatant was collected. Subsequently, proteins were separated by SDS-PAGE and transferred onto PVDF membranes. The membranes were blocked with either 5% non-fat milk or 5% BSA at room temperature (RT) for 1 h and then incubated overnight with antibodies at 4 °C.

### RT-qPCR

Total RNA was extracted from cells using TRIeasy^TM^ Total RNA Extraction Reagent according to the manufacturer’s instruction. Subsequently, 1 μg of this RNA was reverse-transcribed using M-MLV reverse transcriptase (Promega, Madison, WI) to synthesize cDNAs. Quantitative real-time PCR was performed using SYBR qPCR Master Mix (TransGen Biotech, Beijing, China). The primer sequences are listed in the Supplementary Tables. Relative gene expression was calculated using the 2^-ΔΔCT^ method, with β-actin serving as the normalization.

### Immunofluorescent staining (IF)

Cells were fixed with 4% (w/v) paraformaldehyde (PFA) for 10 min at room temperature (RT), and then washed with PBS for 5 min. Next, the cells were permeabilized with 0.5% Triton X-100 for 10 min at RT. After an additional 5 min wash with PBS, the cells were blocked with 5% goat serum for 1 h and incubated with primary antibodies at 4 °C overnight. The next day, the cells were washed three times with PBS containing 0.05% (v/v) Tween 20 (PBST) and incubated with secondary antibodies for 1 h at RT. The cells were washed three more times with PBST before the nuclei were stained with DAPI and the cytoskeleton were stained with Alexa Fluor 660-conjugated Phalloidin (Thermo-Fisher Scientific). Cell images were taken using an Olympus FV1000 microscope (Tokyo, Japan) with a 100× or 60× oil-immersion objective lens.

### Cell viability assay

A total of 2 × 10^3^ cells/well were seeded in 96-well plates 24 h before being treated with nutrient-deficient medium Earle’s balanced salt solution (EBSS) (Thermo-Fisher Scientific) or 500 nM Rapamycin (MedChemExpress). Cell viability was then evaluated using the Cell Counting Kit-8 (CCK-8) (YEASEN, Shanghai, China).

### The orthotopic murine model of OC

Eight-week-old female NOD-SCID mice (Charles River Laboratories, Beijing, China) were anaesthetized with isoflurane. Then, the left ovaries were exposed from the dorsal side and subjected to intrabursal injection of 2 × 10^6^ stable SK-OV-3 cells. In these cells, endogenous RRN3 was depleted by shRNA targeting the 3’ UTR region, and wild-type or mutant (S199A and S199D) RRN3 were ectopically expressed and introduced. The mice were sacrificed approximately 1.2 months post-inoculation when the diameter of OC xenografts reached approximately 1 cm. Primary tumor weight and ascites volume were evaluated.

### Paired normal tissue and OC specimens

Paired normal ovarian tissues and primary ovarian carcinoma specimens were divided into two portions: one was placed in TRIzol reagent for RNA extraction, and the other was fixed in 4% paraformaldehyde for immunohistochemistry and immunofluorescence analyses.

### H&E staining and IHC

The tissues dissected from mice were fixed in 4% paraformaldehyde, dehydrated, paraffin-embedded, consecutive sectioned at a thickness of 5μm, and then deparaffinized. Immunohistochemistry (IHC) and Hematoxylin-Eosin (HE) staining were performed as previously described [41].

### Statistical analysis

All statistical analyses were conducted using Prism 5.0 software (GraphPad Software, San Diego, CA, USA). Most statistical analyses used paired or unpaired Student’s t-tests, except that the statistical analysis of cell proliferation used two-way ANOVA. Three separate biological replicates were used for all experiments.

## Supplementary information

Supplementary Figures

Supplementary material

Original data

Supplementary file 7

Supplementary file 8

Supplementary File 1-6

## Data Availability

PAR-CLIP-seq data were deposited GEO (Gene Expression Omnibus) with accession code GSE286919. CRISPR Knockout screening data were deposited GEO (Gene Expression Omnibus) with accession code GSE286920. RNA polymerase II ChIP-seq data were deposited GEO (Gene Expression Omnibus) with accession code GSE286558.
