# Supplementary material for: Nutrient stress diverts RRN3 from rRNA transcription to alternative polyadenylation of autophagy mRNAs in ovarian cancer
Source: Cell Death Dis. 2025 Nov 21;16(1):849. doi: 10.1038/s41419-025-08142-6 (PMC12638823; doi:10.1038/s41419-025-08142-6)
Supplement: Supplementary file 1 — Supplementary Figures [file 41419_2025_8142_MOESM1_ESM.docx]

Supplementary Materials for

**Nutrient stress diverts RRN3 from rRNA transcription to alternative polyadenylation of autophagy mRNAs in ovarian cancer**

Jianying Lv *et al.*

*Correspondence to: Yi Shi ([yishi@nankai.edu.cn](mailto:yishi@nankai.edu.cn)), Longlong Wang([wangl@nankai.edu.cn](mailto:wangl@nankai.edu.cn)), Beilei Zeng ([qinlin_zeng@126.com](mailto:beileizeng@126.com))


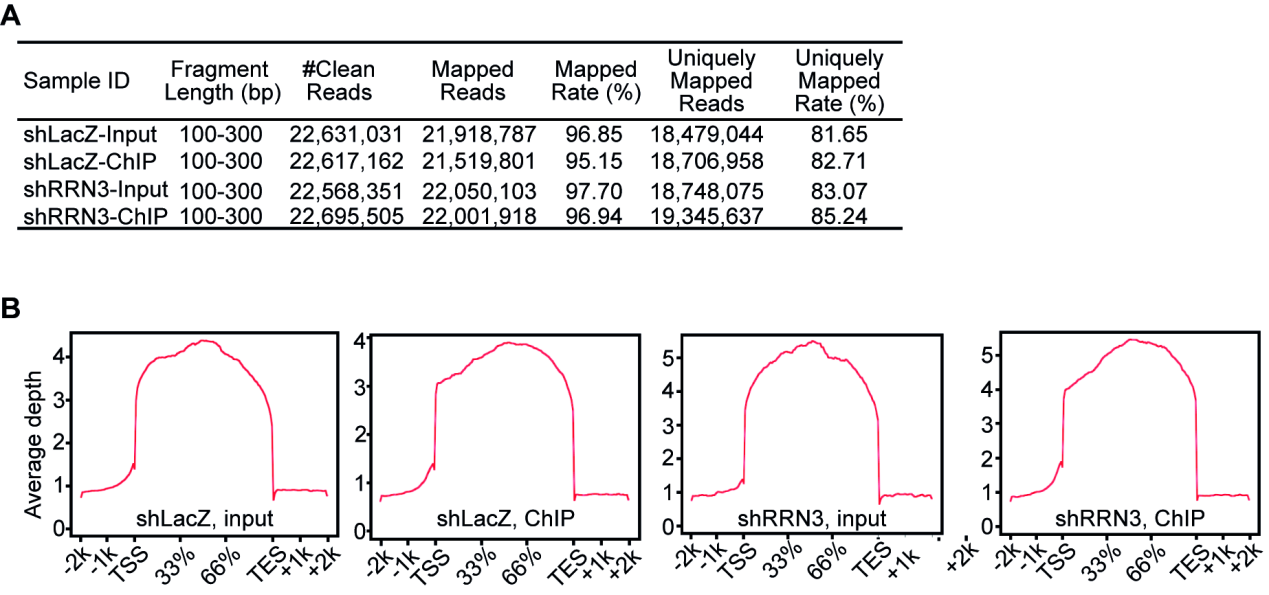


**Figure S1.** The analysis of RNA polymerase II ChIP-seq. (**A**) Summary of RNA polymerase II ChIP sequencing data for each sample (shLacZ and shRRN3). The genome mapping ratio of each sample is shown. (**B**) Gene depth distribution of each sample (shLacZ and shRRN3). The horizontal axis represents different gene regions while the vertical axis represents effective sequencing depth of the gene region.


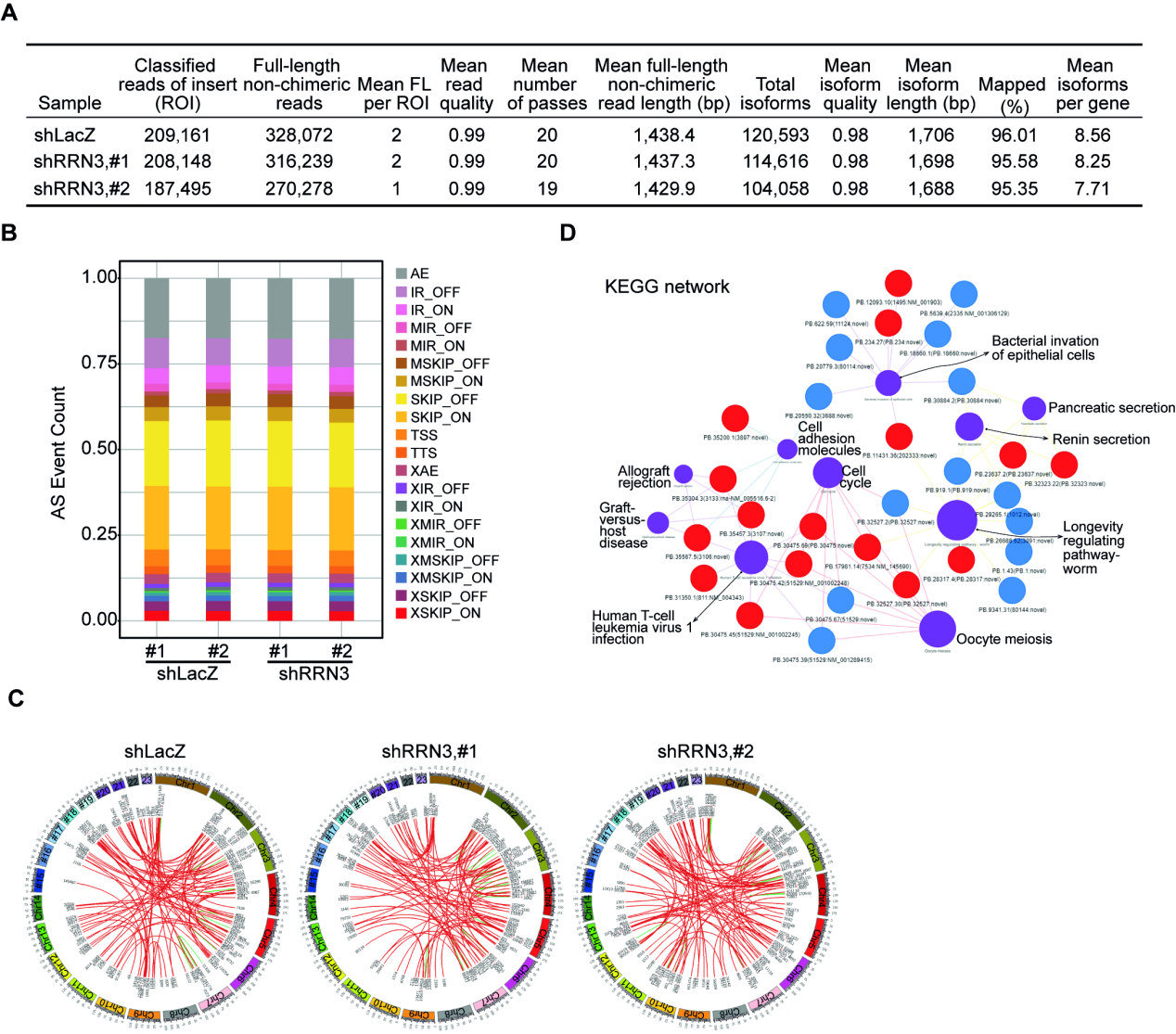


**Figure S2.** The analysis of long-read sequencing data in SK-OV-3 cells transfected with shLacZ or shRRN3. (**A**) Summary of PacBio sequencing data for each sample (shLacZ, shRRN3#1 and shRRN3#2). (**B**) Statistical chart of alternative splicing. X-axis: alternative splicing type, Y-axis: number of alternative splicing, color: sample. (**C**) Circos diagram of fusion genes for each sample (shLacZ, shRRN3#1 and shRRN3#2). (**D**) KEGG-DEG (differentially expressed transcript) relationship network diagram.


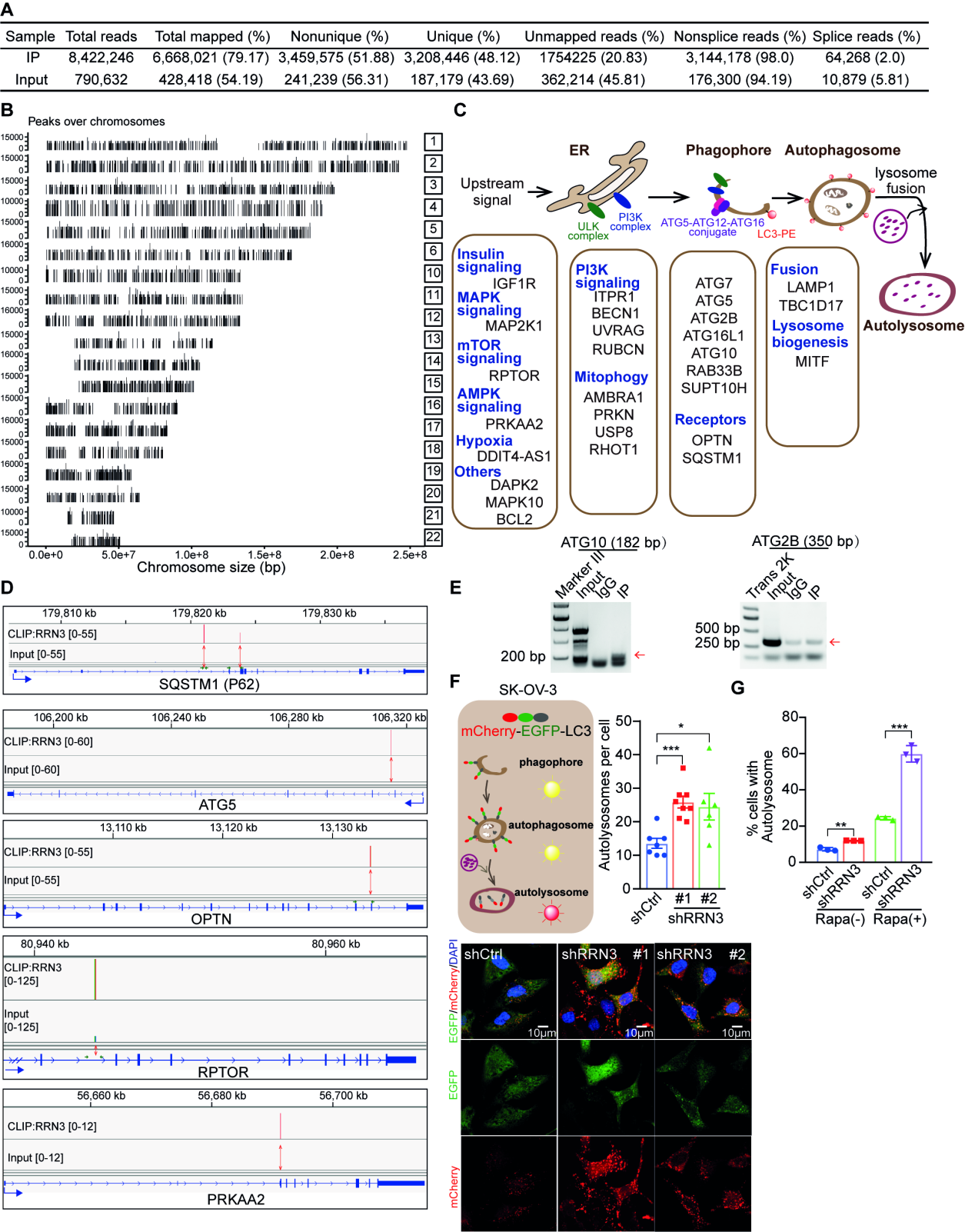


**Figure S3.** The analysis of PAR-CLIP data. (**A**) Alignment of the sequence with the reference genome. (**B**) The distribution of reads on chromosomes. The horizontal axis represents the length of the chromosome, the right side represents the chromosome number, and the left vertical axis represents the depth of read coverage on each chromosome. (**C**) autophagy-related mRNA bound by RRN3 identified by PAR-CLIP. (**D**) Representation of the binding sites where RRN3 binds to mRNAs associated with autophagy. (**E**) The PCR verification of RRN3-binding sites on autophagy-related mRNAs identified by PAR-CLIP. (**F**) SK-OV-3 cells stably expressing mCherry-EGFP-LC3 were stably transfected with shCtrl/shRRN3#1/shRRN3#2, respectively. The number of autolysosomes was counted. Data are shown as means ± SEM, ***p<0.001, *p<0.05 by unpaired two-sided Student’s t-test. (**G**) The analysis of autolysosomes in SK-OV-3 cells with or without RRN3 knockdown and Rapamycin stimulation by Flow cytometry. Data are shown as means ± SEM, ***p<0.001, **p<0.01 by unpaired two-sided Student’s t-test.


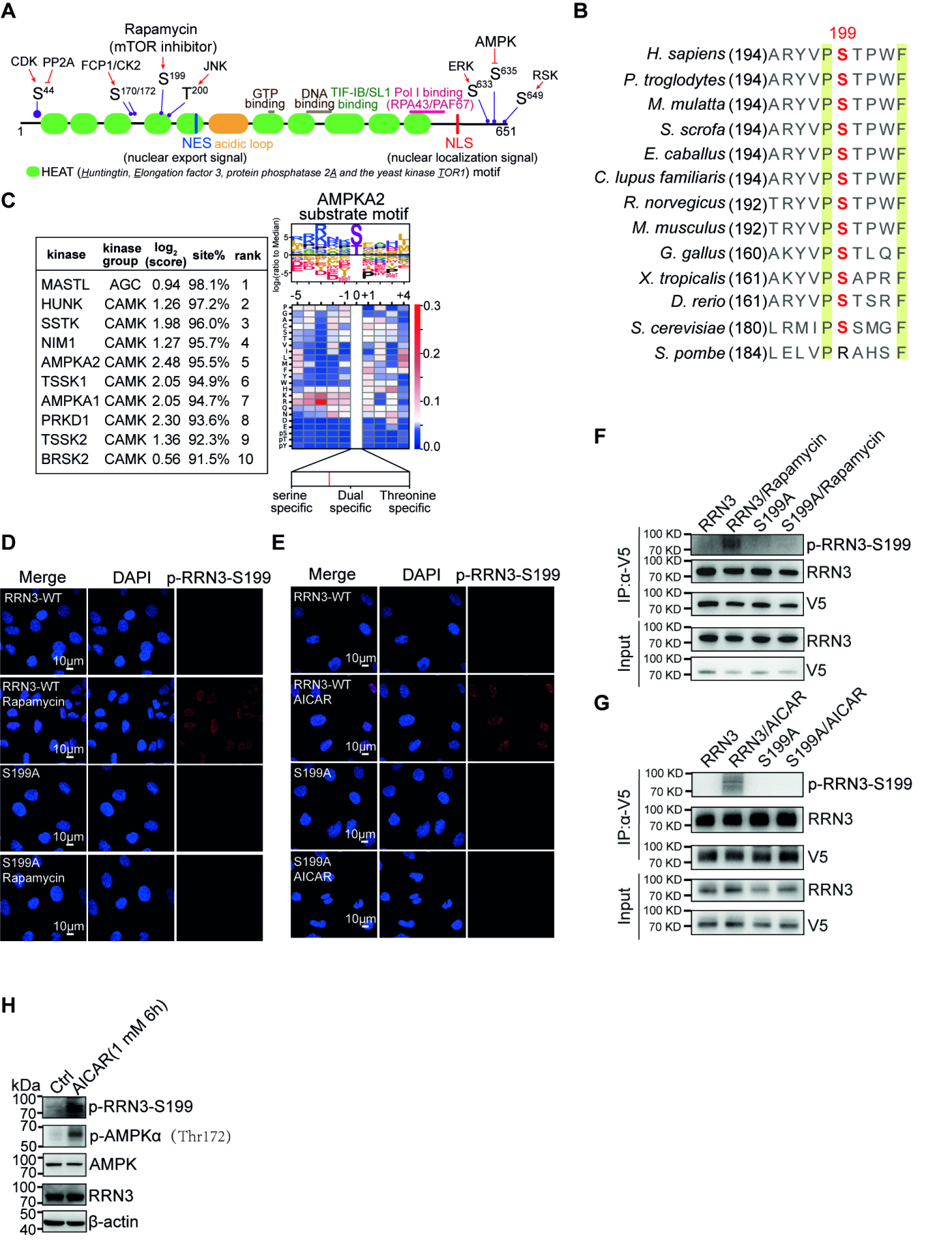


**Figure S4.** RRN3 S199 is a rapamycin-sensitive phosphorylation site targeted by AMPK. (**A**) Reported phosphorylation sites on RRN3. (**B**) The alignment of serine 199-flanking sequences of RRN3 in indicated species. (**C**) The substrate-motif-based prediction of kinases capable of phosphorylating S199 residue of RRN3. (**D**)SK-OV-3 cells transfected with wild-type RRN3 or the phosphorylation-deficient mutant RRN3^S199A^ were treated with DMSO or Rapamycin(500 nM, 24h). Immunofluorescence staining was performd using a phospho-specific antibody against p-RRN3-S199. (**E**) SK-OV-3 cells transfected with wild-type RRN3 or the phosphorylation-deficient mutant RRN3^S199A^ were treated with DMSO or AICAR(1 mM, 6h). Immunofluorescence staining was performd using a phospho-specific antibody against p-RRN3-S199. (**F**,**G**) Cell expessing V5-tagged wild-type RRN3 (RRN3^WT^) or the S199A mutant (RRN3^S199A^) were treated with Rapamycin (F) or AICAR (G). Cell lysates were subjected to immunoprecipitation using anti-V5 antibody, followed by immunobloting with phospho-specific RRN3 S199 (p-RRN3-S199) antibody. (**H**)Western blot analysis showing AMPK activation and RRN3 Ser199 phosphorylation in SK-OV-3 cells treated with 1 mM AICAR for 6 hours.


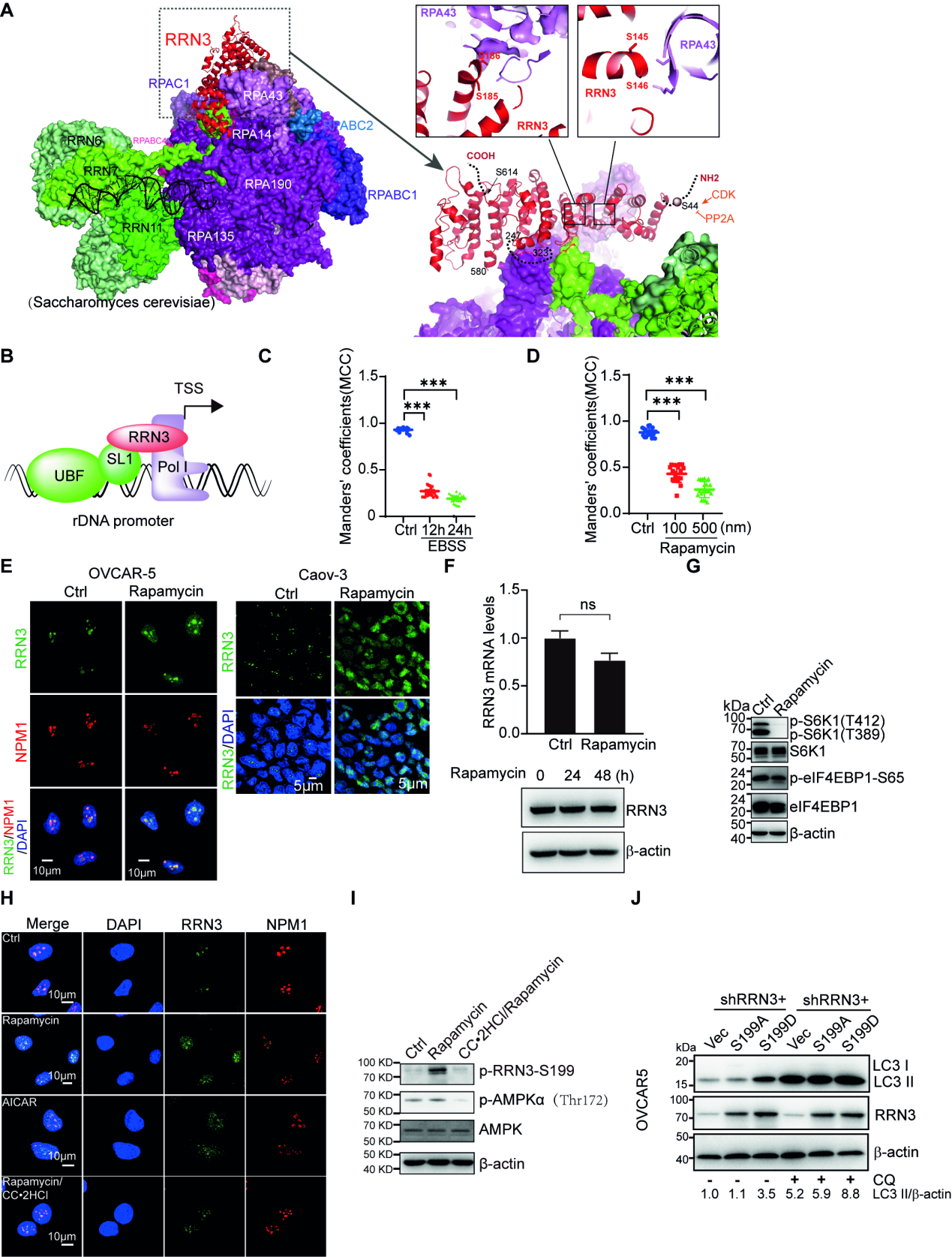


**Figure S5.** Nutrient deficiency and the mimicking rapamycin treatment divert RRN3 out of nucleolus by phosphorylation at S199.(**A**) The structure analysis of Saccharomyces cerevisiae RNA polymerase I complex (PDB ID: 6RUI) highlighting the position of S199 of human RRN3 (S185 in S. cerevisiae RRN3) near the interface of RRN3 and Pol I.(**B**) Essential steps for the initiation of RNA polymerase I transcription. (**C, D**) Manders’ colocalization coefficient (MCC) was used to evaluate the overlap of RRN3 with NPM1 in different conditions. SK-OV-3 cells cultured in complete medium (Ctrl) or nutrient-depleted EBSS buffer for indicated periods (C) or treated with Rapamycin or DMSO (Ctrl) for 24 h (D). (**E**) Analysis of RRN3 localization after treatment with rapamycin by immunofluorescence in OVCAR-5 cells and Caov-3 cells. (**F**) Changes in RRN3 mRNA and protein levels following rapamycin treatment were analyzed using qRT-PCR and WB. Data are shown as means ± SEM, ns, not significant, by unpaired two-sided Student’s t-test. (**G**)SK-OV-3 cells were treated with 500 nM Rapamycin for 24 h, and p-S6K1, S6K1,p-eIF4EBP1-S65 and eIF4EBP1 were detected by Western blot. (**H**) SK-OV-3 cells were treated with DMSO, 500 nM Rapamycin for 6 h, 1 mM AICAR for 6 h, or co-treated with 10 µM Dorsomorphin 2HCl (CC•2HCl) and 500 nM Rapamycin for 6 h. Cells were then subjected to immunofluorescence co-staining for NPM1 and RRN3. (**I**) SK-OV-3 cells were treated with DMSO, 500 nM Rapamycin for 6 h, or co-treated with 10 µM Dorsomorphin 2HCl (CC•2HCl) and 500 nM Rapamycin for 6 h. Western blot was performed to detect the phosphorylation level of RRN3 at Ser199. (**J**) Western blot analysis of LC3 with or without 10 μM CQ for 4 h in OVCAR-5 cells stably expressing Vector, RRN3^S199A^and RRN3^S199D^ respectively.


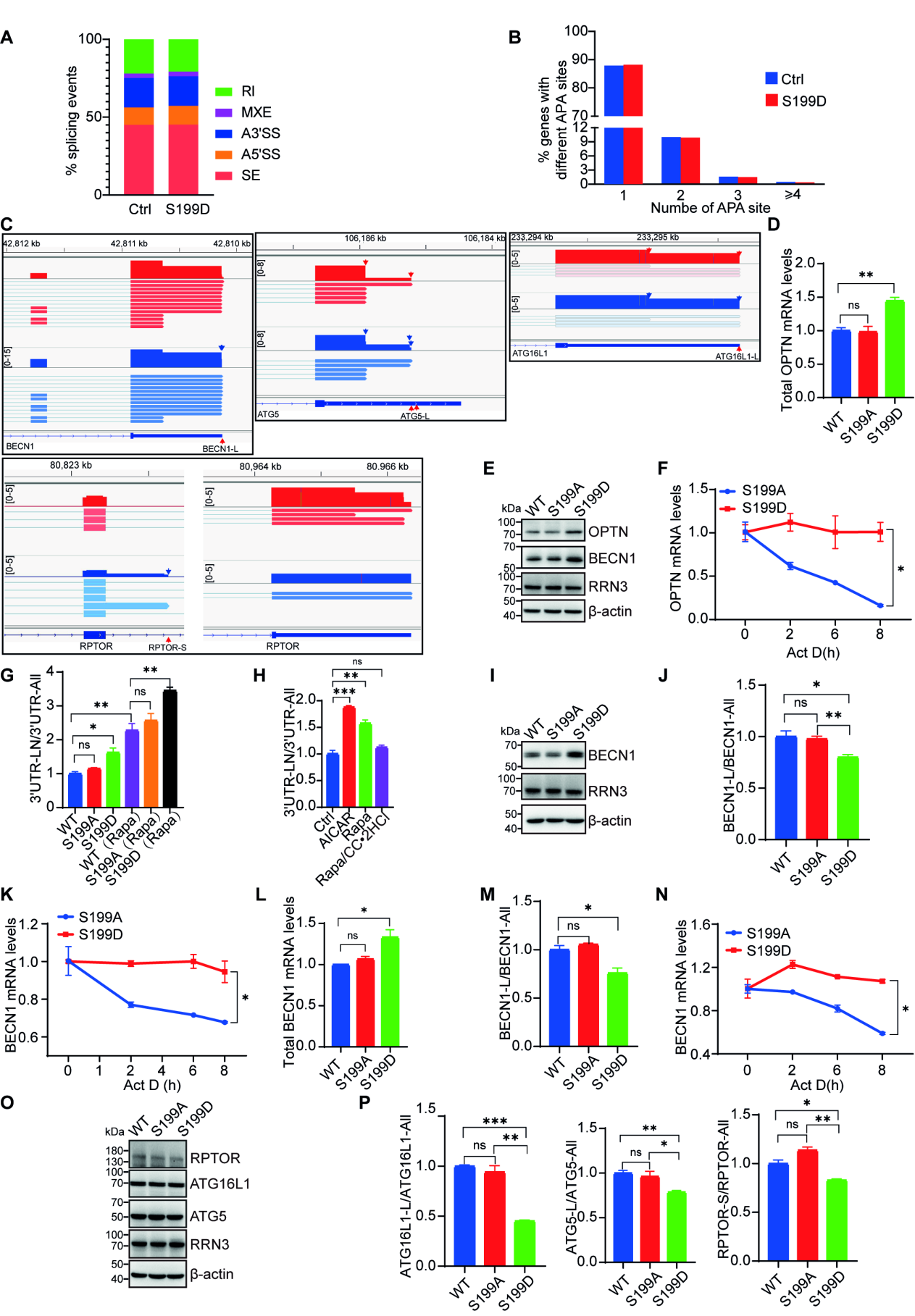


**Figure S6.** The analysis of long-read sequencing data in SK-OV-3 cells transfected with Vector or RRN3^S199D^. (**A**) Statistical chart of alternative splicing. SE(Skipped exon); A5’ SS(Alternative 5’ splice site); A3’ SS(Alternative 3’ splice site); MXE(Mutually exclusive exons); RI(Retained intron). (**B**) The barplot of APA sites number in different groups(Vector or RRN3^S199D^). (**C**) Representation of different APA sites of autophagy-related genes by IGV. (**D**) RT-qPCR analysis of the mRNA levels of OPTN in indicated OVCAR-5 cells (means±SEM,**p<0.01, ns, not significant, by unpaired Student’s t-test). (**E**) The protein levels of OPTN and BECN1 in indicated OVCAR-5 cells were analyzed by Western blot. (**F**) The mRNA stability of OPTN was measured by RT-qPCR at the indicated time points post treatment by 10 μg/mL actinomycin D (Act D) in OVCAR-5 cells expressing RRN3^S199A^ or RRN3^S199D^ (means ± SEM, *p<0.05, by unpaired Student’s t-test). (**G**) RT-qPCR analysis of the ratio between amplicons (3’ UTR-LN/ 3’ UTR-All) in indicated OVCAR-5 cells treated with or without Rapamycin (500 nM, 24 h) (means ± SEM, **p<0.01, *p<0.05, ns, not significant, by unpaired Student’s t-test). (**H**) RT-qPCR analysis of the 3’ UTR-LN/ 3’ UTR-All amplicon ratio was used to assess APA isoform changes in SK-OV-3 cells treated with DMSO, 1 mM AICAR for 6 h, 500 nM Rapamycin for 6 h, or co-treated with 10 µM Dorsomorphin 2HCl (CC•2HCl) and 500 nM Rapamycin for 6 h. (**I**) The protein levels of BECN1 in indicated SK-OV-3 cells were analyzed by Western blot. (**J**) RT-qPCR analysis of the ratio between amplicons (BECN1-L/ BECN1-All) represents APA isoform changes in SK-OV-3 cells expressing WT or S199-mutated RRN3 (means±SEM,**p<0.01, *p<0.05, ns, not significant, by unpaired Student’s t-test). (**K**) The mRNA stability of BECN1 was measured by RT-qPCR at the indicated time points post treatment by 10 μg/mL actinomycin D (Act D) in SK-OV-3 cells expressing RRN3^S199A^ or RRN3^S199D^ (means ± SEM, *p<0.05, by unpaired Student’s t-test). (**L**) RT-qPCR analysis of the mRNA levels of BECN1 in indicated OVCAR-5 cells(means±SEM, *p<0.05, ns, not significant, by unpaired Student’s t-test). (**M**) RT-qPCR analysis of the ratio between amplicons (BECN1-L/ BECN1-All) represents APA isoform changes in OVCAR-5 cells expressing WT or S199-mutated RRN3 (means±SEM, *p<0.05, ns, not significant, by unpaired Student’s t-test). (**N**) The mRNA stability of BECN1 was measured by RT-qPCR at the indicated time points post treatment by 10 μg/mL actinomycin D (Act D) in OVCAR-5 cells expressing RRN3^S199A^ or RRN3^S199D^ (means ± SEM, *p<0.05, by unpaired Student’s t-test). (**O**) The protein levels of ATG16L1, ATG5 and RPTOR in indicated SK-OV-3 cells were analyzed by Western blot. (**P**) RT-qPCR analysis of the ratio between different isoforms and total transcript amplicons (ATG16L1-L/ ATG16L1-All, ATG5-L/ATG5-All, RPTOR-S/RPTOR-All) represents APA isoform changes in SK-OV-3 cells expressing WT or S199-mutated RRN3 (means±SEM,***p<0.001, **p<0.01, *p<0.05, ns, not significant, by unpaired Student’s t-test).
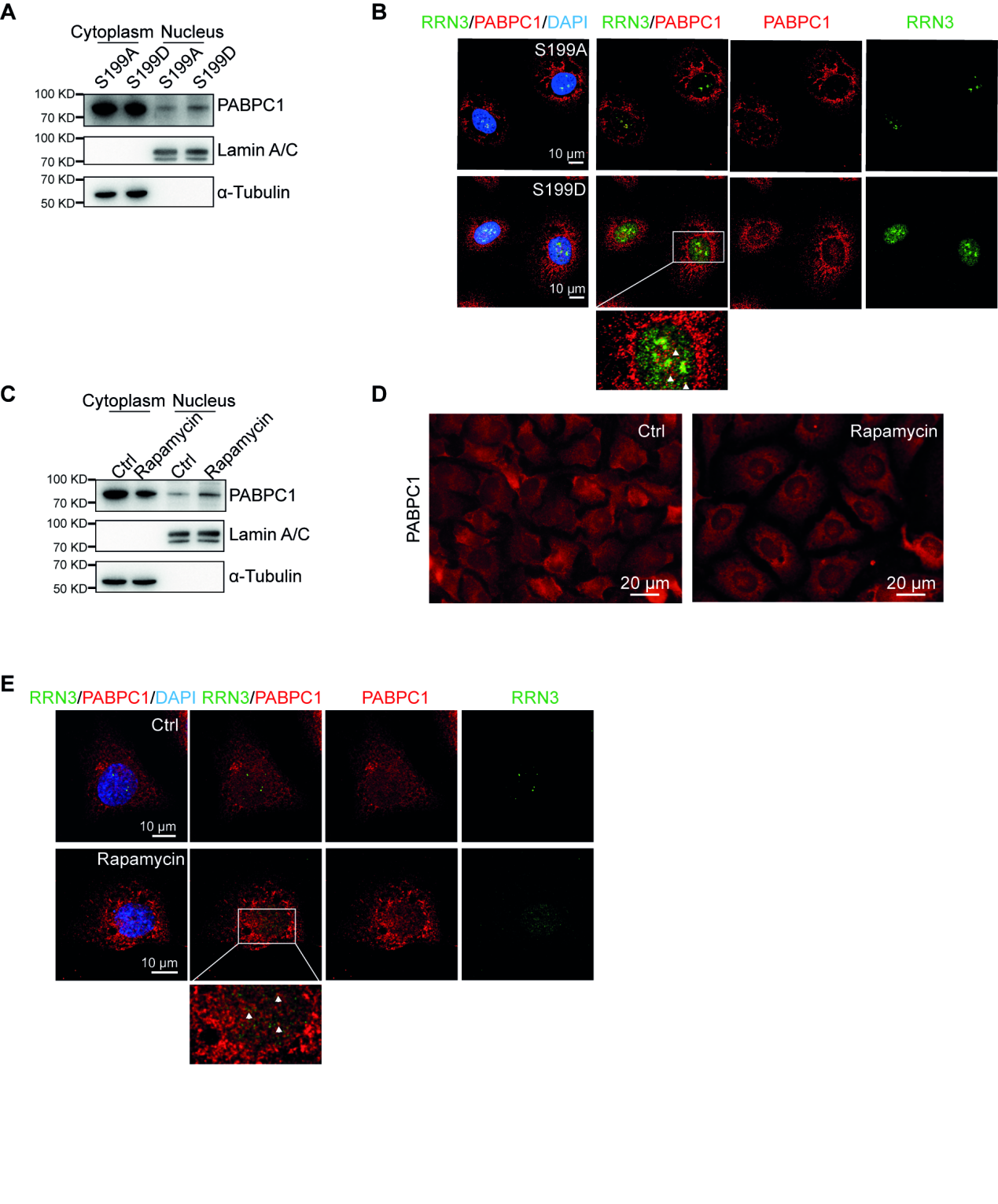


**Figure S7.** RRN3 interacts with PABPC1. (**A**) SK-OV-3 stable cell lines expressing either RRN3^S199A^ or RRN3^S199D^ mutants were used. Nuclear and cytoplasmic fractions were isolated and analyzed by Western blotting to assess the subcellular distribution of PABPC1. Lamin A/C and α-Tubulin were used as nuclear and cytoplasmic markers, respectively. (**B**) Representative images showing the co-localization of S199-mutated RRN3 and PABPC1. (**C**) SK-OV-3 cells were treated with 500 nM Rapamycin for 24 hours. Nuclear and cytoplasmic fractions were isolated and analyzed by Western blotting to determine the subcellular distribution of PABPC1. LaminA/C and α-Tubulin were used as nuclear and cytoplasmic markers, respectively. (**D**) Representative immunofluorescent images of the subcellular localization of endogenous PABPC1 in SK-OV-3 cells treated with 500 nM Rapamycin or DMSO (Ctrl) for 24 h. (**E**) Representative immunofluorescent images showing the co-localization of RRN3 and PABPC1 in the nucleus of SK-OV-3 cells treated with Rapamycin(500 nM, 24 h).
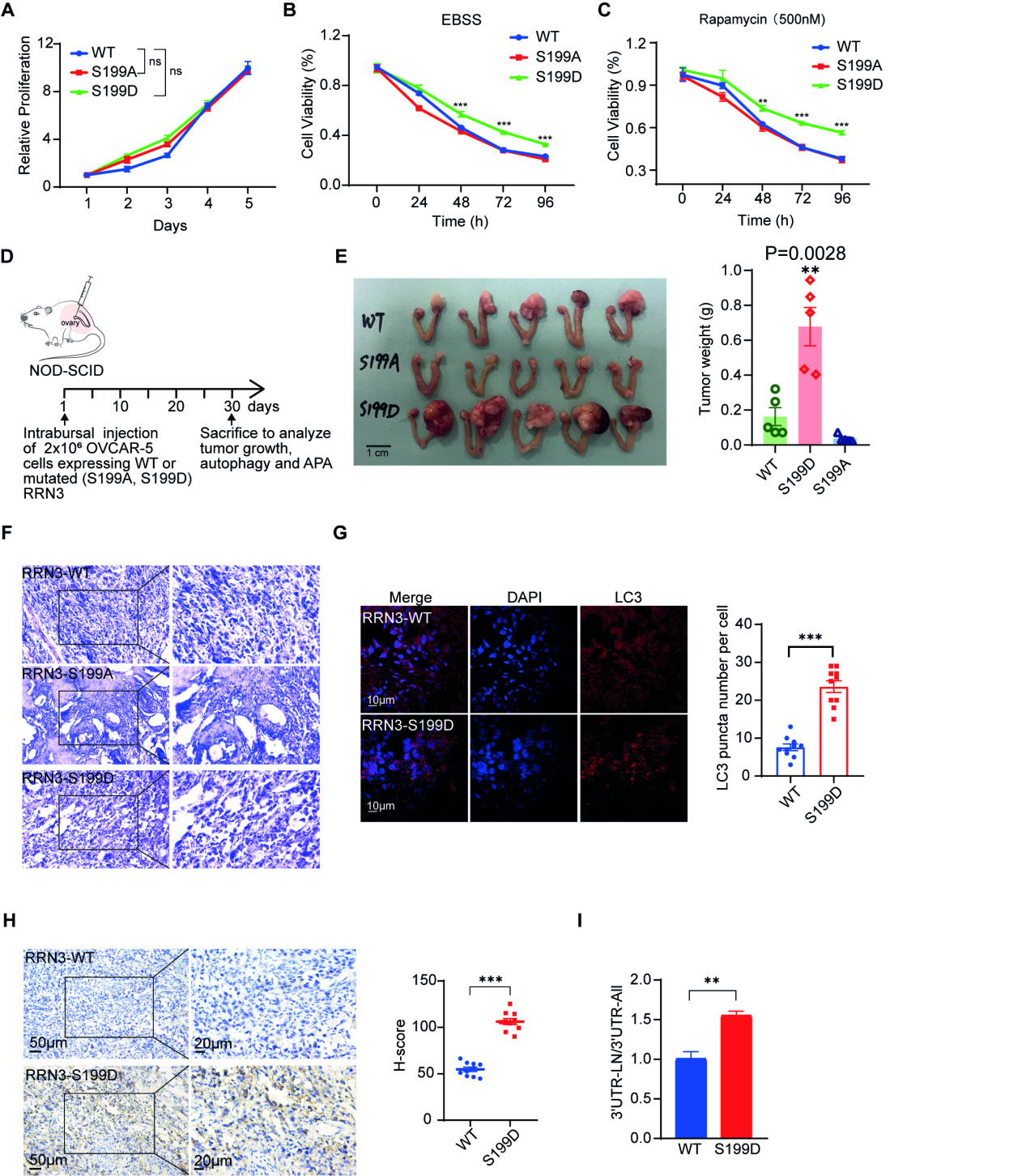


**Figure S8.** RRN3-regulated autophagy is essential for OC progression. (**A**) The proliferation of OVCAR-5 cells harboring indicated RRN3 was assessed by CCK-8 assay. (**B**, **C**) The viability of indicated OVCAR-5 cells cultured in nutrient-deficient EBSS buffer (B) or treated with Rapamycin (500 nM) (C) was analyzed by CCK-8 assay. (means±SEM, ***p<0.001, **p<0.01, ns, not significant, by unpaired Student’s t-test). (**D**) Schematic of the orthotopic model of ovarian cancer. OVCAR-5 cells expressing RRN3^WT^ or mutated RRN3 (RRN3^S199A^, RRN3^S199D^) were injected into the bursal of ovary of NOD-SCID mice (n=5). (**E,F**) The tumor xenografts were dissected (E) for the measurement of tumor weight (right bar graph),which were verified by H&E staining (F). Data are shown as means±SEM (n=5), **p<0.01, by unpaired Student’s t-test. (**G**) Representative immunofluorescent images and quantification ( right bar graph) of the tumor sections stained with DAPI and anti-LC3 antibody. (**H**) Immunohistochemical staining (left panels) and quantification (right bar graph) of OPTN in the tumor xenograft sections. Data are shown as means±SEM, ***p<0.001, by unpaired Student’s t-test. (**I**) RT-qPCR analysis of the ratio between amplicons (3’ UTR-LN/ 3’ UTR-All) of OPTN in the tumor xenografts (means±SEM, **p<0.01, by unpaired Student’s t-test).
