# Supplementary material for: Nutrient stress diverts RRN3 from rRNA transcription to alternative polyadenylation of autophagy mRNAs in ovarian cancer
Source: Cell Death Dis. 2025 Nov 21;16(1):849. doi: 10.1038/s41419-025-08142-6 (PMC12638823; doi:10.1038/s41419-025-08142-6)
Supplement: Supplementary file 2 — Supplementary material [file 41419_2025_8142_MOESM2_ESM.docx]

**Table S1.** Key Resources table

| Antibody | COMMD3 | Invitrogen  A304-092A | WB: 1:1000 |
| --- | --- | --- | --- |
| Antibody | BMI1 | Proteintech  10832-1-AP | WB: 1:1000 |
| Antibody | RRN3 | Proteintech  25918-1-AP | WB: 1:1000  IF: 1:100 |
| Antibody | OPTN | Proteintech  10837-1-AP | WB: 1:2000  IHC: 1:200 |
| Antibody | LC3 | Cell Signaling Technology 3868S | WB: 1:1000 |
| Antibody | V5 | Invitrogen  Cat No. R960-25 | WB: 1:10000 |
| Antibody | FLAG | Proteintech  Cat No. 20543-1-AP | WB: 1:1000 |
| Antibody | NPM1 | Proteintech  60096-1-Ig | IF: 1:100 |
| Antibody | RRN3 | Sigma  HPA049837 | IF: 1:50 |
| Antibody | LC3 | Proteintech  14600-1-AP | WB: 1:1000  IF: 1:100 |
| Antibody | β-actin | Affinity  AF7018 | WB: 1:5000 |
| Antibody | RNA polymerase II | Active motif  91151 | ChIP |
| Antibody | mCherry | Proteintech  26765-1-AP | WB: 1:1000 |
| Antibody | GFP | Proteintech  50430-2-AP | WB: 1:2000 |
| Antibody | RPTOR | ABclonal A8992 | WB: 1:500 |
| Antibody | ATG5 | ABclonal A19677 | WB: 1:1000 |
| Antibody | ATG16L1 | Proteintech  29445-1-AP | WB: 1:3000 |
| Antibody | BECN1 | ABclonal A21191 | WB: 1:2000 |
| Antibody | AMPK | ABclonal A27795 | WB: 1:0000 |
| Antibody | AMPK | Proteintech  10929-2-AP | WB: 1:1000 |
| Antibody | p-AMPKα（Thr172） | CST #2535 | WB: 1:1000 |
| Antibody | p70-S6K1 | ABclonal A4898 | WB: 1:1000 |
| Antibody | Phospho-p70 S6 Kinase 1 | ABclonal AP0564 | WB: 1:500 |
| Antibody | eIF4EBP1 | ABclonal A24691 | WB: 1:1000 |
| Antibody | Phospho-eIF4EBP1-S65 | ABclonal AP1363 | WB: 1:1000 |
| Antibody | LaminA/C | Proteintech  10298-1-AP | WB: 1:1000 |
| Antibody | α-Tubulin | Proteintech  11224-1-AP | WB: 1:1000 |
| agent | AICAR | MCE HY-13417 |  |
| agent | Dorsomorphin 2HCl | Selleck S7306 |  |
| Recombinant DNA reagent | pLV-H1-EF1α-puro（plasmid） |  |  |
| Recombinant DNA reagent | pLV-EF1α-MCS-IRES-Bsd（plasmid） |  |  |
| Recombinant DNA reagent | Pet-20b(+)vector（plasmid） |  |  |
| Recombinant DNA reagent | pGEX-6p-1（plasmid） |  |  |

**Table S2.** Primers for PCR/qPCR

| Primer name | Forward Sequence (5’-3’) | Reverse Sequence (5’-3’) |
| --- | --- | --- |
| TRT (+) | CAACATCAAGTTGGACATCAC | TCGCCCTTGCTCACTTCTGCTTG |
| TRT (-) | CAACATCAAGTTGGACATCAC | TATCTTTCCCTCTTAACCCTTTCAC |
| β-actin | ACCCTGAAGTACCCCATCGAG | GGATAGCACAGCCTGGATAGCAA |
| GAPDH | GGAGCGAGATCCCTCCAAAAT | GGCTGTTGTCATACTTCTCATGG |
| RRN3 | CCAGACATAAAGAGATTGCCTTGG | GGAGGCACAAAATGGGAAGC |
| COMMD3 | ACGGATGTTTCTTGGCGCTTG | CAGCTGAAGGCAAGGCGTTTC |
| COMMD3-BMI1 | AACGCCTTCACGCTTCTCCT | GCACACACATCAGGTGGGGAT |
| OPTN | TTGGGCATCGTGTCTGAACT | GCCTCCTTGAGTGCAACTTC |
| RPTOR | TCTGTCGGCATCTTCCCCTA | GTACTTGTGGCCGTTGTCCT |
| BECN1 | ATGGTGGCTTTCCTGGACTG | TTGATGGAATAGGAGCCGCC |
| ATG5 | AGACCTTCTGCACTGTCCATC | GCAATCCCATCCAGAGTTGCT |
| ATG16L1 | GCATGACGTACCAAACAGGC | ACTCCCCACGTTTCTTGTGT |
| OPTN(mRNA stability) | GGGCAACTTTTGGAGTGACTTT | TGCCTTCTCTGCTTGTAGCC |
| OPTN-3’UTR-All | AGGACAGGGACTGGCGGCAAC | CTCTTGGAGGAAAAAATCTGACATTTAC |
| OPTN-3’UTR-LN | AGGACAGGGACTGGCGGCAAC | ACAGAGTCTCCCTCTGTCACC |
| ATG5-L | TGCCAATGACGACCAGTCAA | TACCGGACCACTGAAGGTCT |
| ATG5-All | AGACCTTCAGTGGTCCGGTA | ATAAGGCAACTGGGCTGGTC |
| ATG16L1-L | GTCCCAACACCTTGGGTTCA | TGGCCAGAATCACCACCTTC |
| ATG16L1-All | CAACGGCAGTGCCAAAATCA | GTTCACCGGGCAAATGAACC |
| BECN1-L | GGAAAGGAGTCCATGGGGTT | TCTTGCTTGGGTGTCCTCAC |
| BECN1-All | TGAGGACACCCAAGCAAGAC | GGCGGCTCCTATTCCATCAA |
| RPTOR-S | CCGGCTGACCTATTCACCTC | CATGACATCACAGTGCCACG |
| RPTOR-All | AGAGCCTTGACCCAACTGTG | ACGATCAAGCCAGCATTGGA |
| BECN1 | ATGGTGGCTTTCCTGGACTG | TTGATGGAATAGGAGCCGCC |

**Table S3.** Primers for PAR-CLIP

| Primer name | Forward Sequence (5’-3’) | Reverse Sequence (5’-3’) |
| --- | --- | --- |
| PRKAA2 | ATACCAGGTGATCAGCACTCC | GTTGAGCACTACCATAAAAGCAAC |
| ATG10 | AGGTGGAATACCCTGTCTCCT | TGAACAGGAGGCTCATCTTGG |
| PRKN | TTGTACCCGTTGAGAGCTGC | AGCAGGCACAGGGTAATCAG |
| ATG7 | TCCCTGCCCCAGAGATTCG | GCAGGGCAGGGTCAGTACC |
| SQSTM1 | CTCACCGTGAAGGCCTACCT | GCGGTAGTGCGCCTGGAA |
| ATG2B | TCCTGCACCTGTTGTCGATG | ACCTCCCTTTGGGCTTTGTC |
