# Supplementary material for: Nutrient stress diverts RRN3 from rRNA transcription to alternative polyadenylation of autophagy mRNAs in ovarian cancer
Source: Cell Death Dis. 2025 Nov 21;16(1):849. doi: 10.1038/s41419-025-08142-6 (PMC12638823; doi:10.1038/s41419-025-08142-6)
Supplement: Supplementary file 3 — Original data [file 41419_2025_8142_MOESM3_ESM.pptx]

## Slide 1
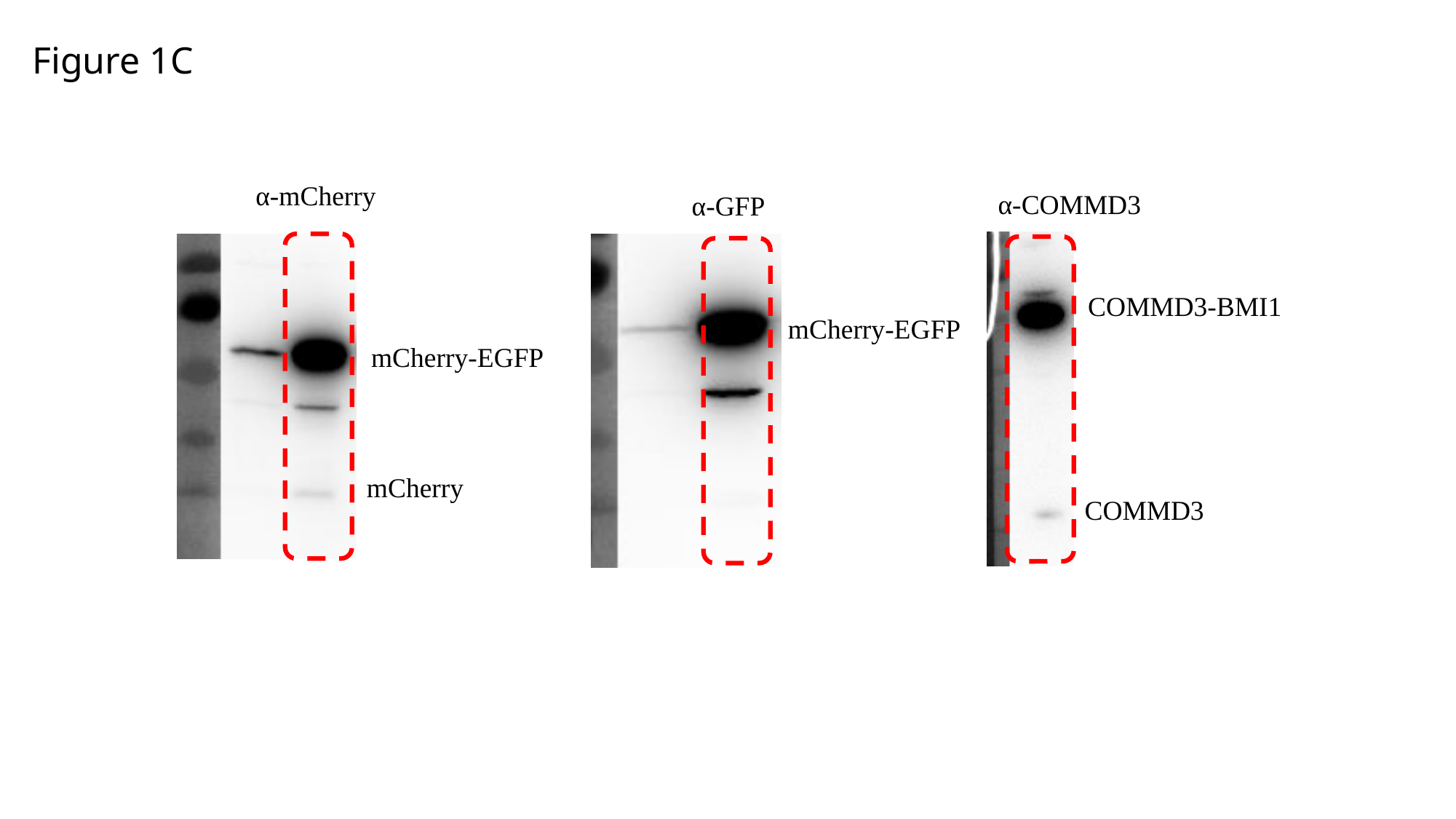

Figure 1C
α-mCherry
α-COMMD3
α-GFP
COMMD3-BMI1
mCherry-EGFP
mCherry-EGFP
mCherry
COMMD3

## Slide 2
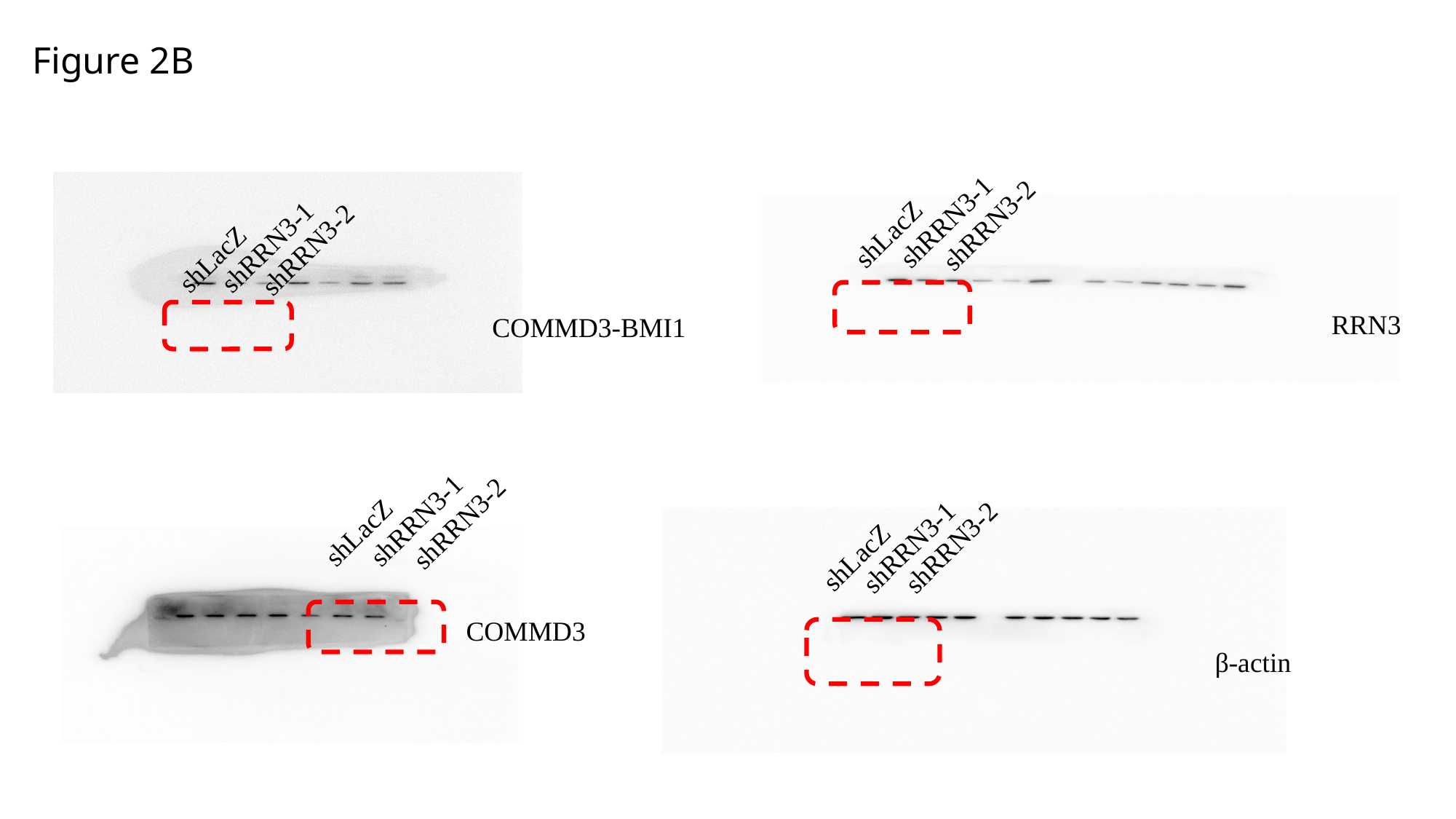

Figure 2B
shLacZ
shRRN3-1
shRRN3-2
COMMD3-BMI1
shLacZ
shRRN3-1
shRRN3-2
RRN3
shLacZ
shRRN3-1
shRRN3-2
COMMD3
shLacZ
shRRN3-1
shRRN3-2
β-actin

## Slide 3
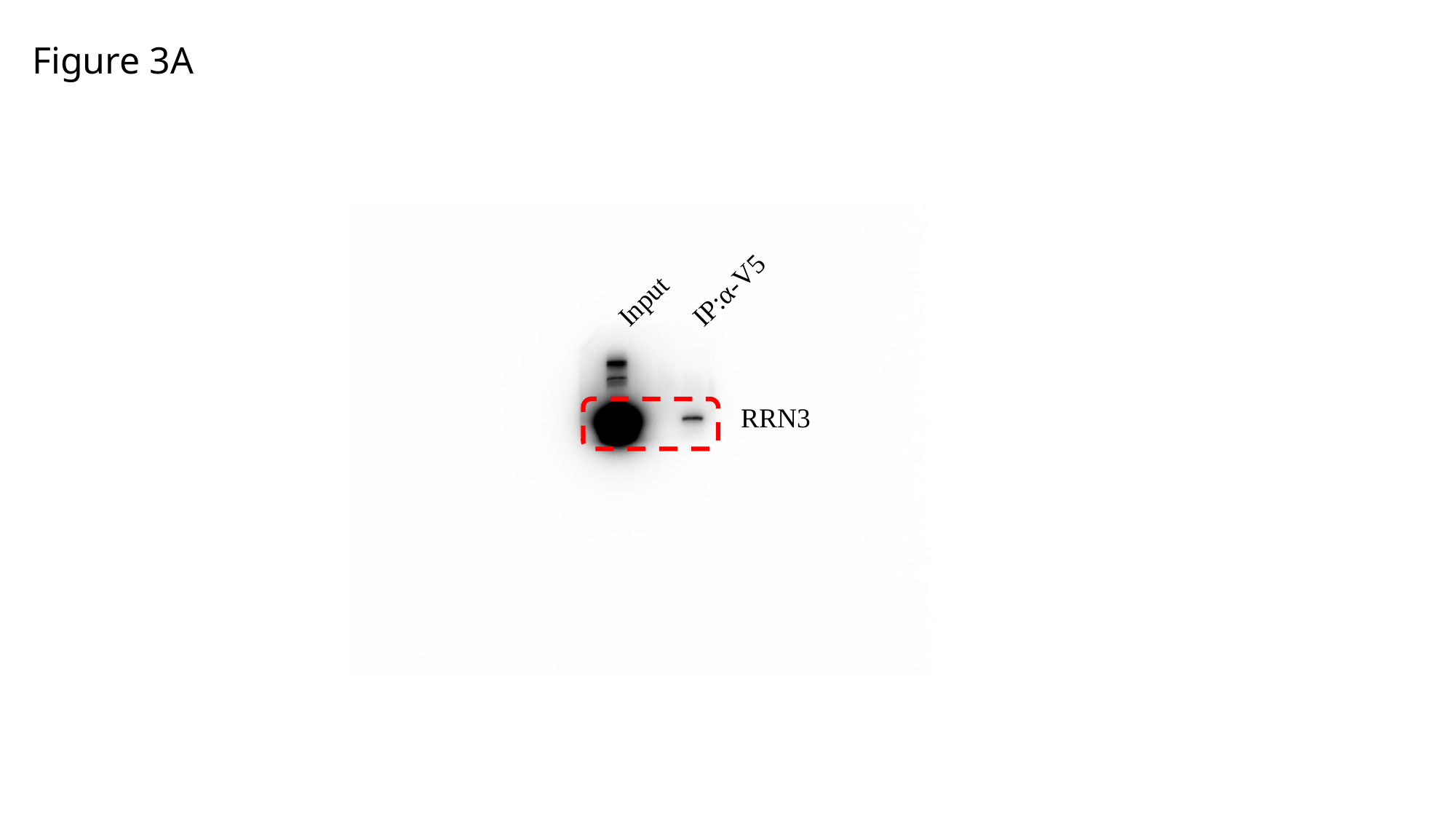

Figure 3A
Input
IP:α-V5
RRN3

## Slide 4
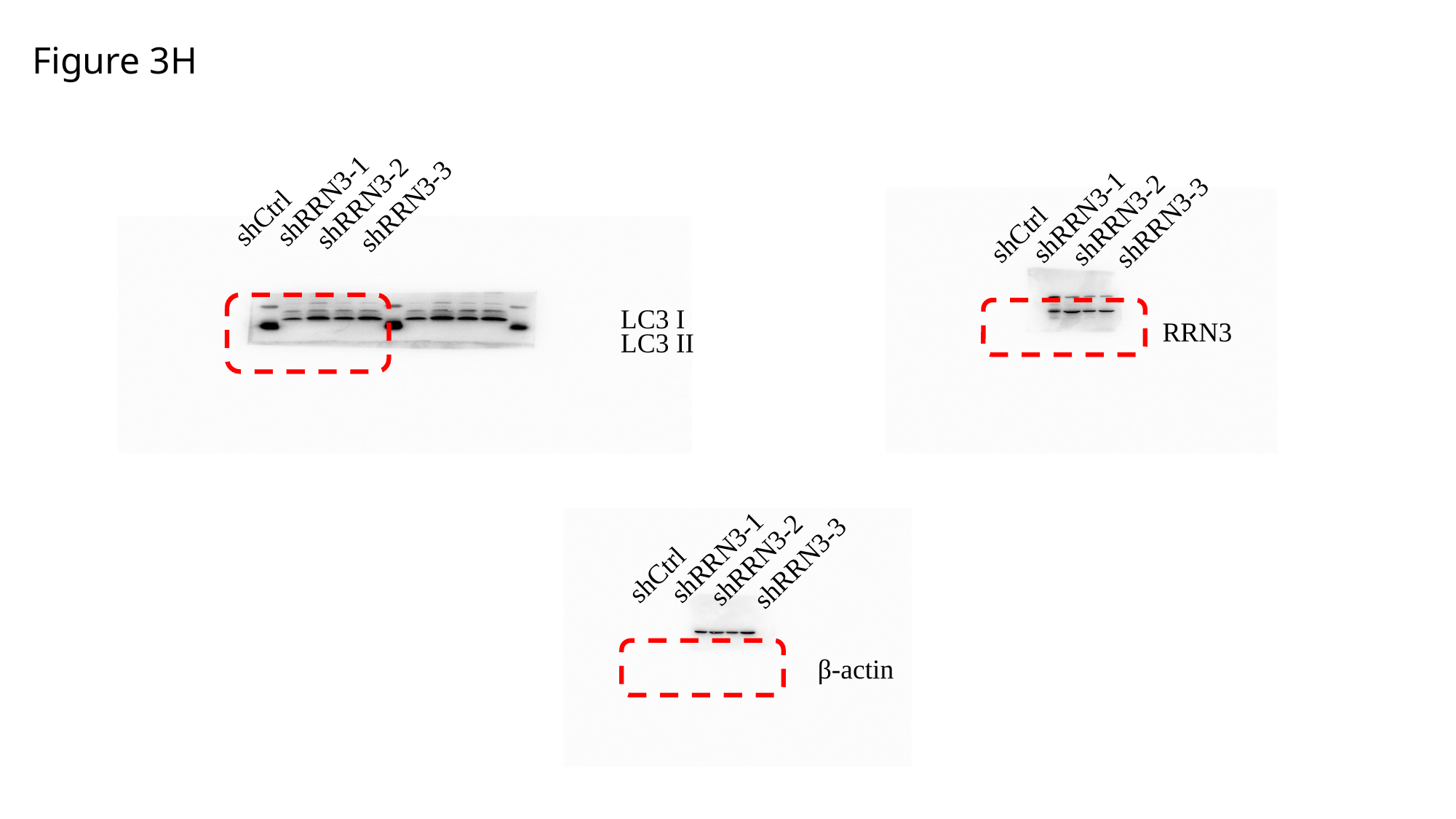

Figure 3H
shCtrl
shRRN3-1
shRRN3-2
shRRN3-3
LC3 I
LC3 II
shCtrl
shRRN3-1
shRRN3-2
shRRN3-3
RRN3
shCtrl
shRRN3-1
shRRN3-2
shRRN3-3
β-actin

## Slide 5
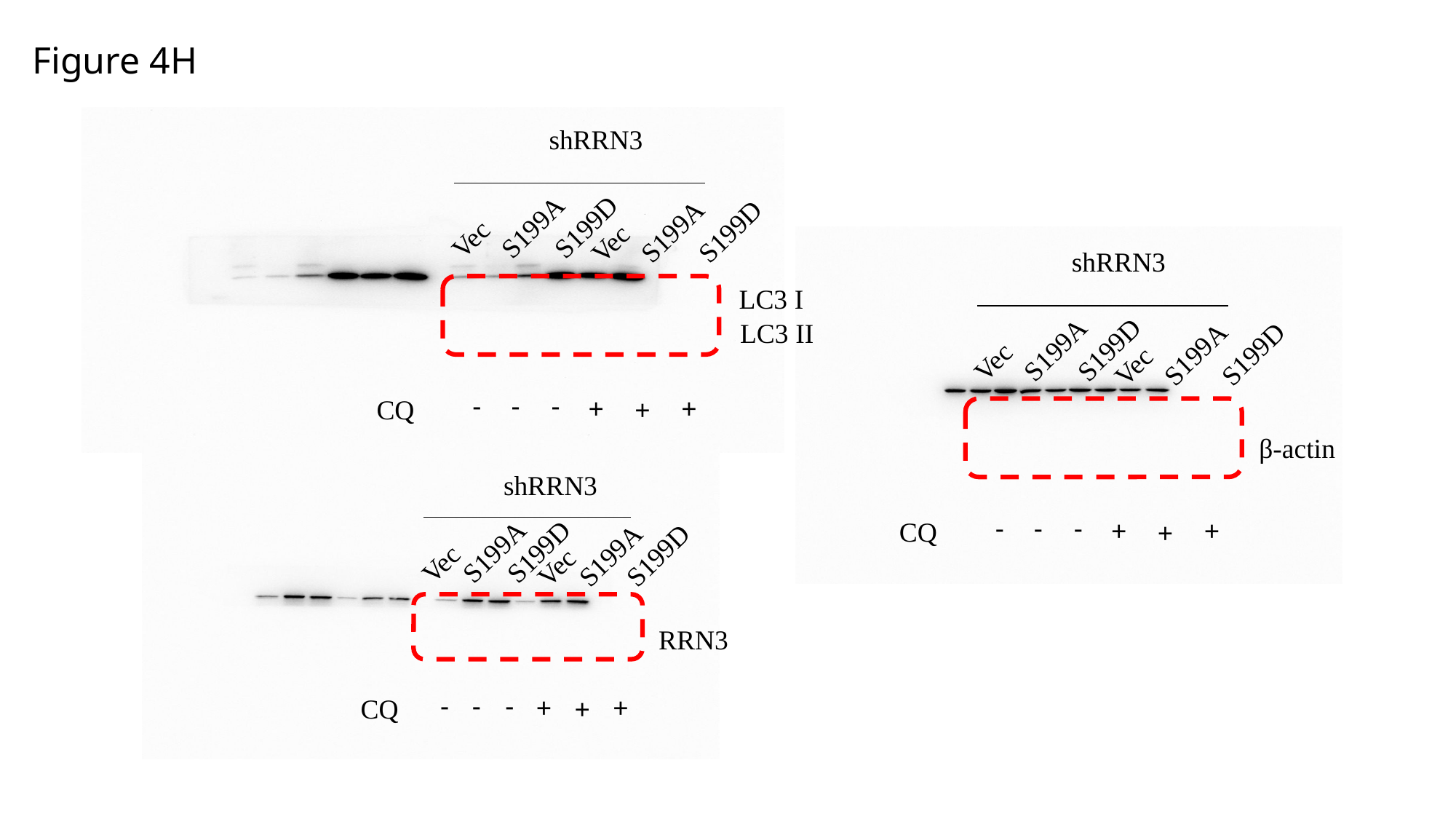

Figure 4H
shRRN3
Vec
S199A
S199D
Vec
S199A
S199D
-
-
-
+
+
CQ
+
shRRN3
Vec
S199A
S199D
Vec
S199A
S199D
β-actin
-
-
-
+
+
CQ
+
LC3 I
LC3 II
shRRN3
Vec
S199A
S199D
Vec
S199A
S199D
RRN3
-
-
-
+
+
CQ
+

## Slide 6
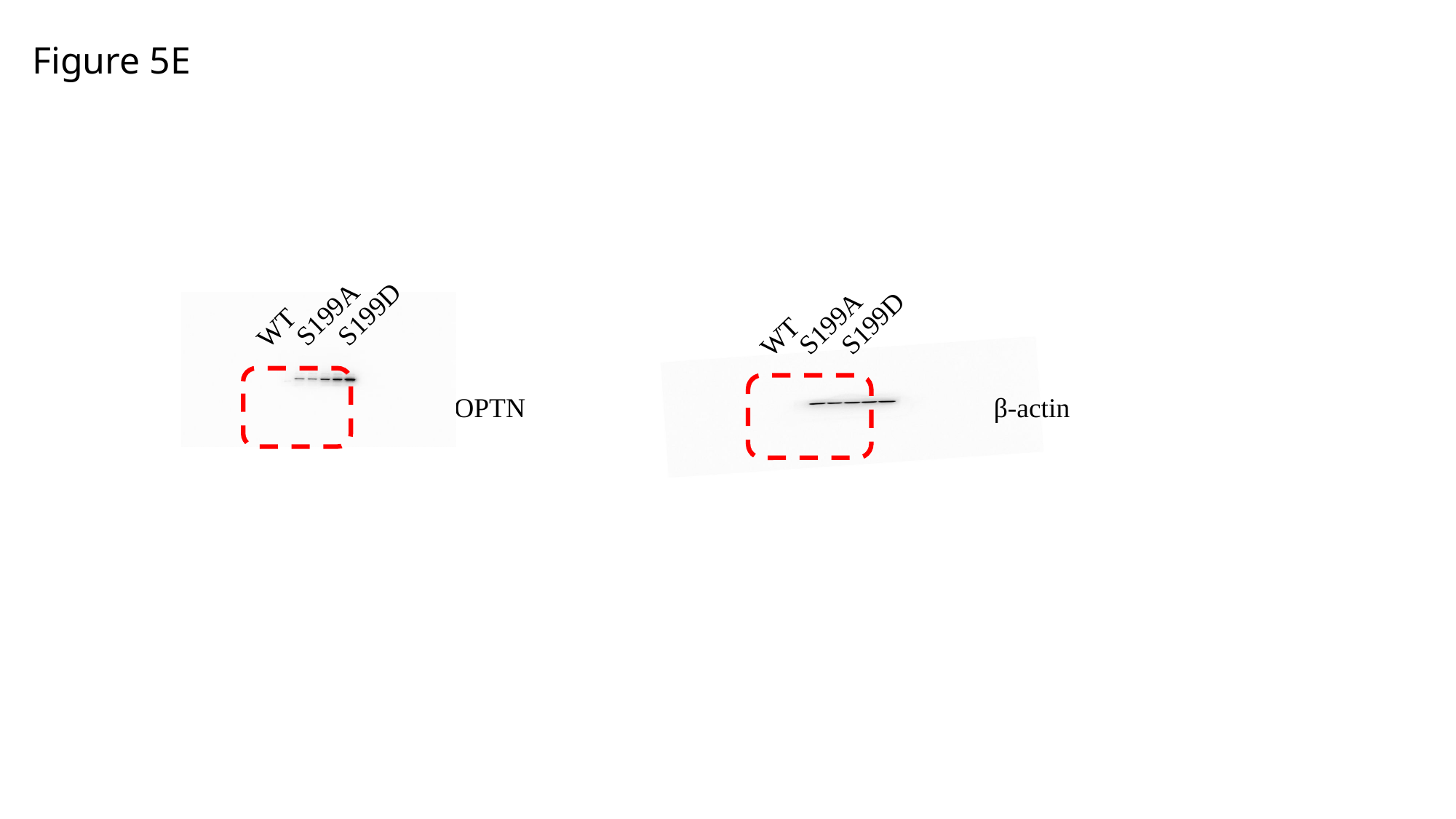

Figure 5E
S199A
S199D
WT
OPTN
S199A
S199D
WT
β-actin

## Slide 7
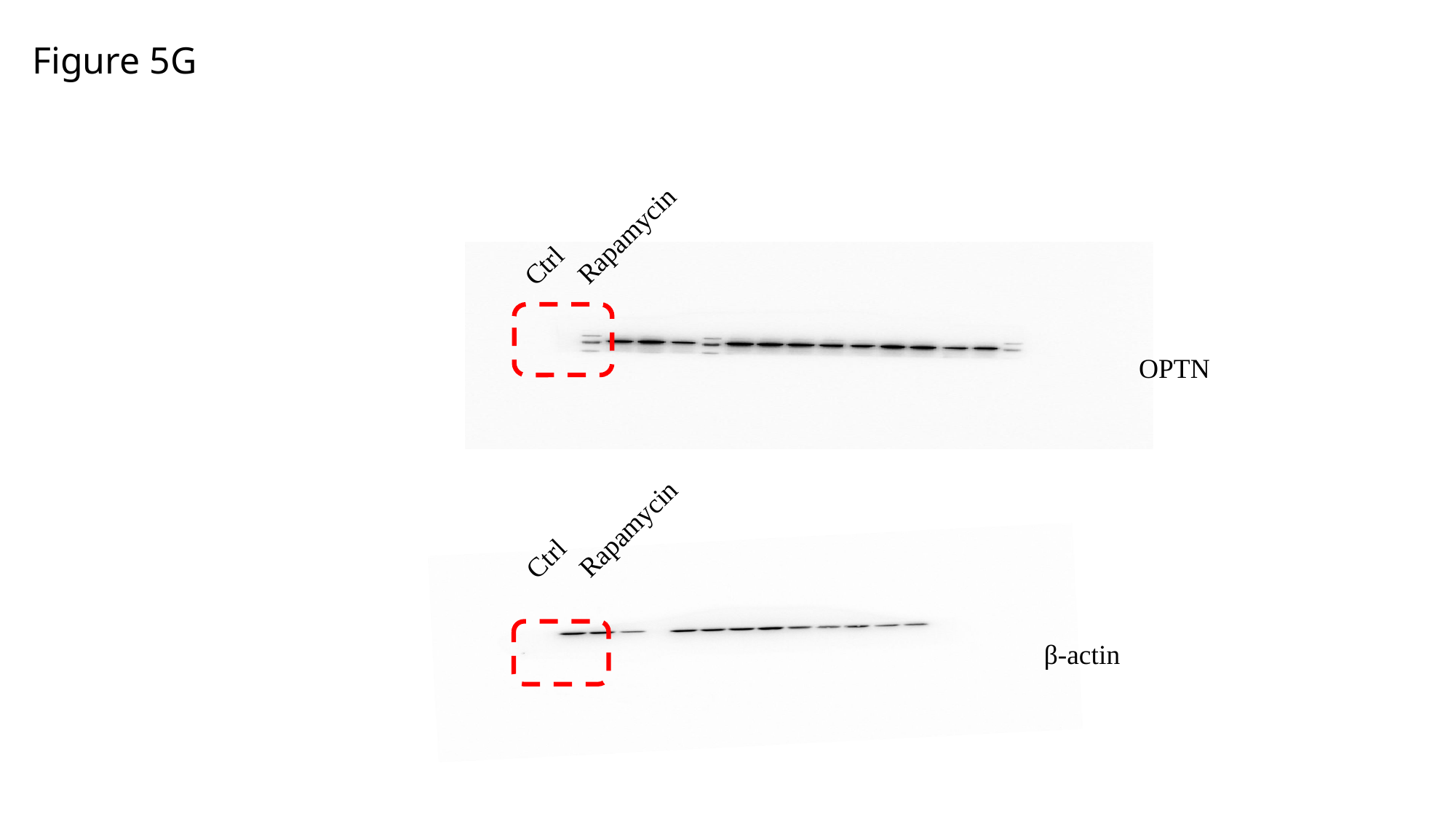

Figure 5G
Rapamycin
Ctrl
OPTN
Rapamycin
Ctrl
β-actin

## Slide 8
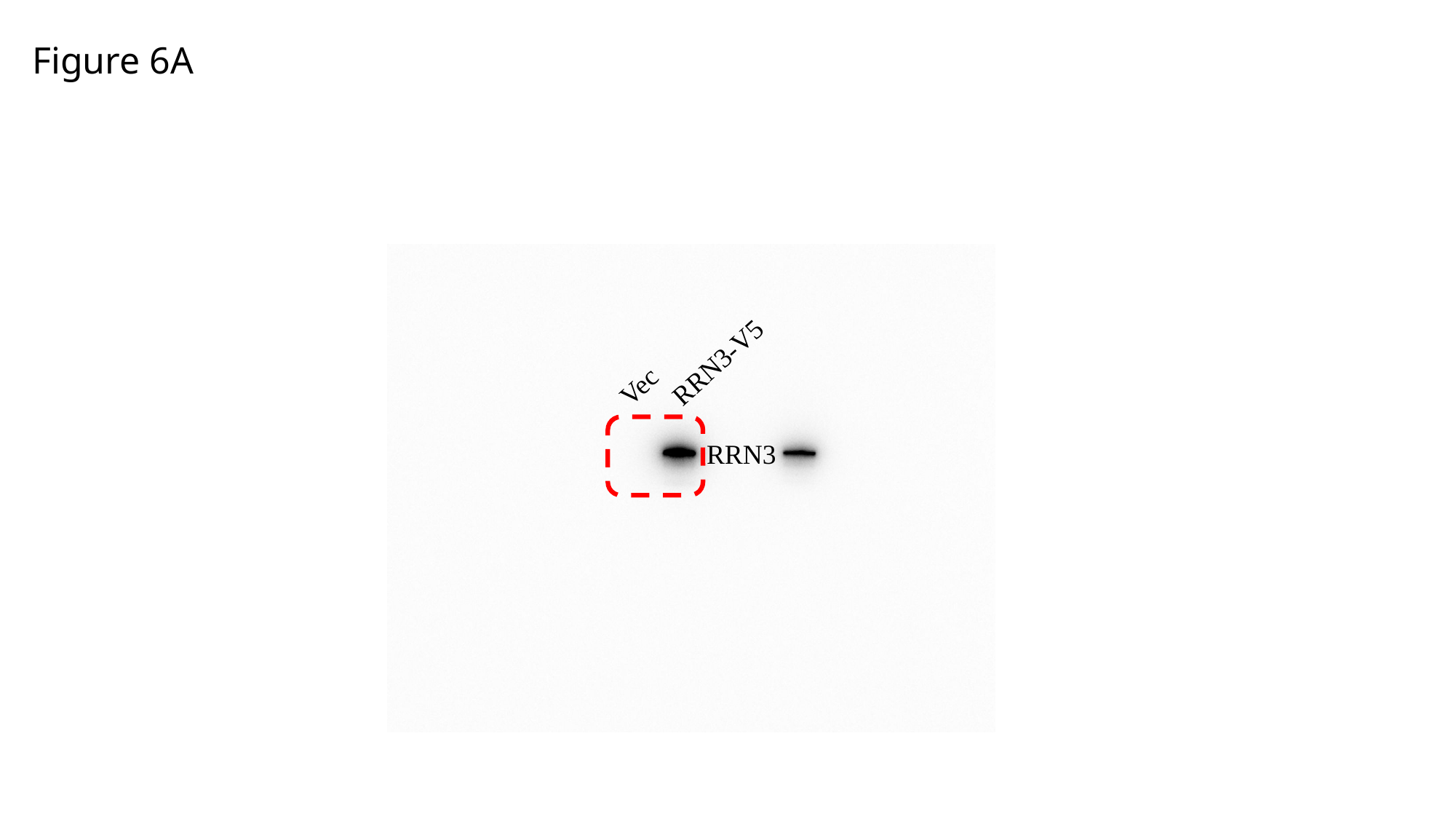

Figure 6A
RRN3-V5
Vec
RRN3

## Slide 9
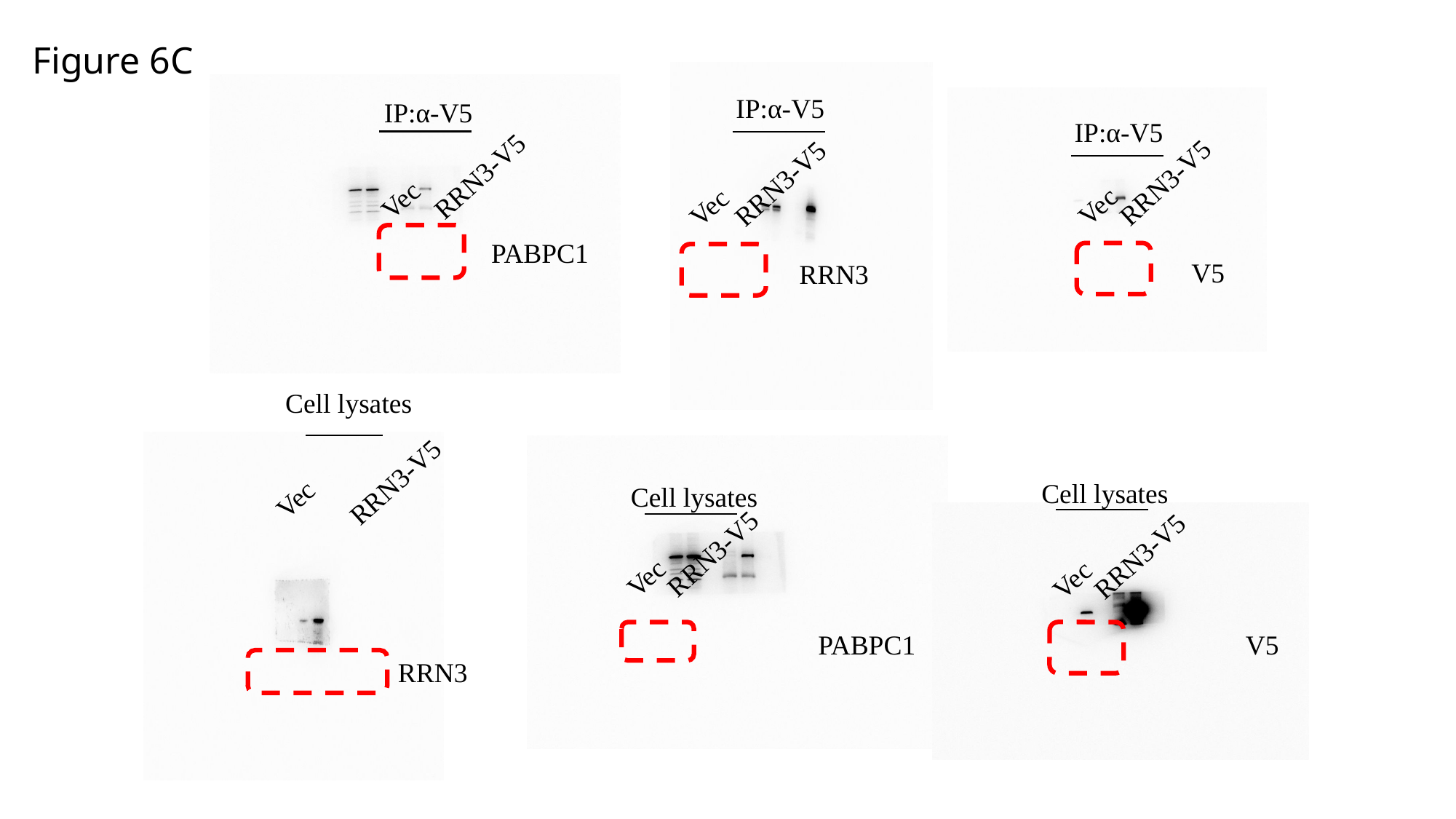

Figure 6C
IP:α-V5
RRN3-V5
Vec
RRN3
IP:α-V5
RRN3-V5
Vec
PABPC1
IP:α-V5
RRN3-V5
Vec
V5
Cell lysates
RRN3-V5
Vec
Cell lysates
RRN3-V5
Vec
PABPC1
Cell lysates
RRN3-V5
Vec
V5
RRN3

## Slide 10
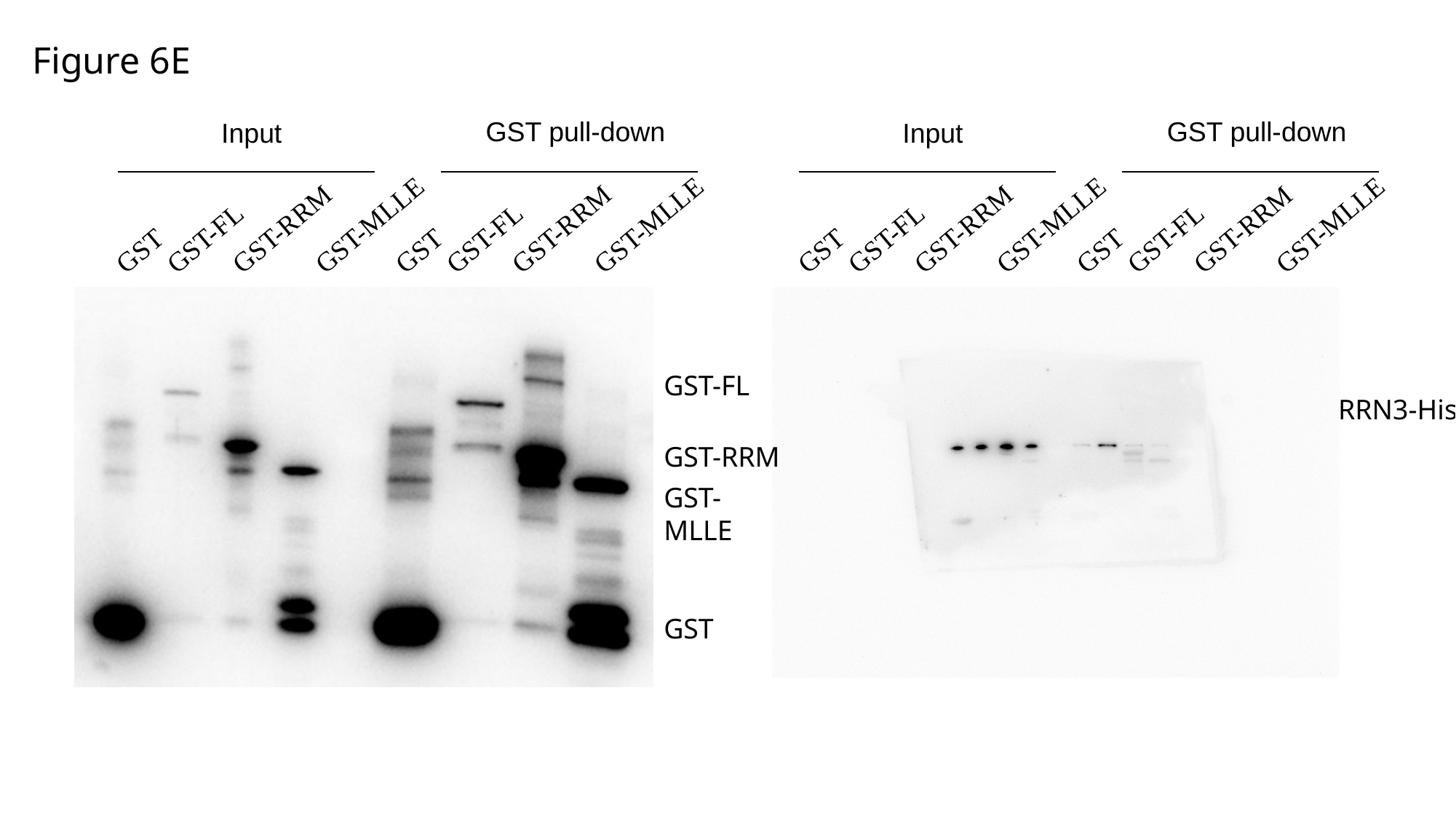

Figure 6E
GST pull-down
GST pull-down
Input
Input
GST-MLLE
GST-MLLE
GST-MLLE
GST-MLLE
GST-FL
GST-RRM
GST-FL
GST-RRM
GST-FL
GST-RRM
GST-FL
GST-RRM
GST
GST
GST
GST
GST-FL
RRN3-His
GST-RRM
GST-MLLE
GST

## Slide 11
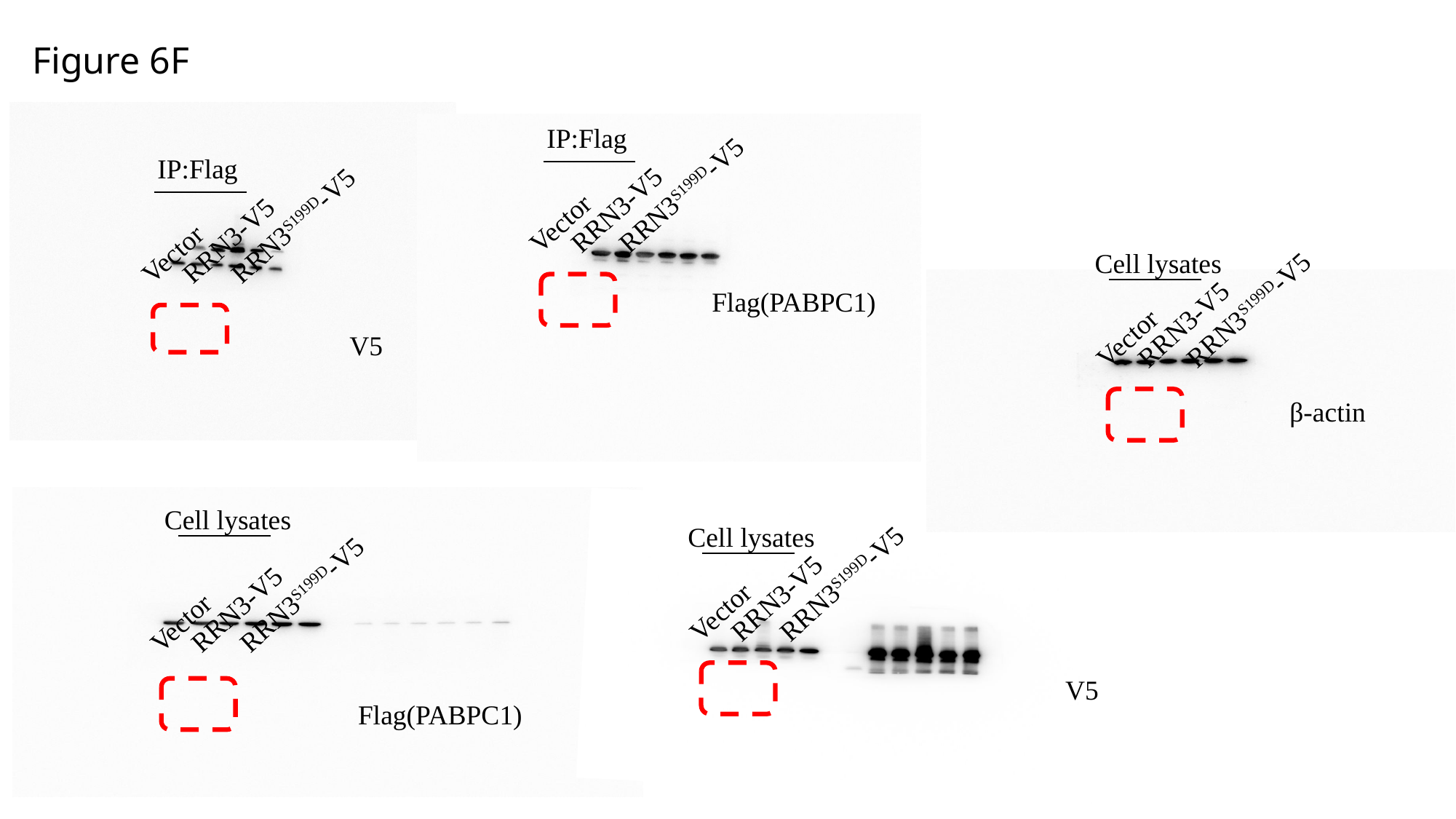

Figure 6F
IP:Flag
RRN3S199D-V5
RRN3-V5
Vector
V5
IP:Flag
RRN3S199D-V5
RRN3-V5
Vector
Flag(PABPC1)
Cell lysates
RRN3S199D-V5
RRN3-V5
Vector
β-actin
Cell lysates
RRN3S199D-V5
RRN3-V5
Vector
Flag(PABPC1)
Cell lysates
RRN3S199D-V5
RRN3-V5
Vector
V5

## Slide 12
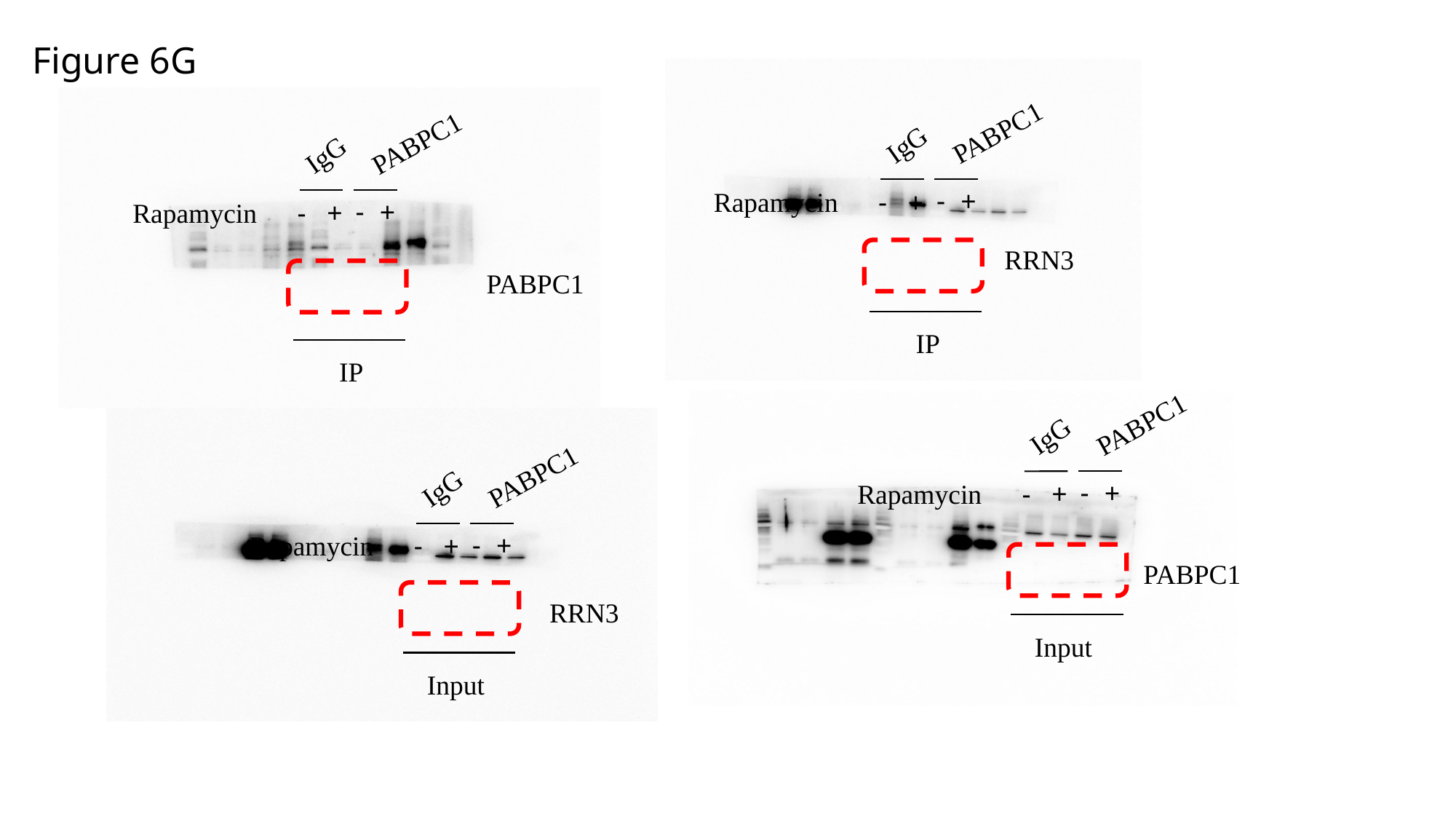

Figure 6G
PABPC1
IgG
-
+
Rapamycin
-
+
RRN3
IP
PABPC1
IgG
-
+
Rapamycin
-
+
PABPC1
IP
PABPC1
IgG
-
+
Rapamycin
-
+
PABPC1
Input
PABPC1
IgG
-
+
Rapamycin
-
+
RRN3
Input

## Slide 13
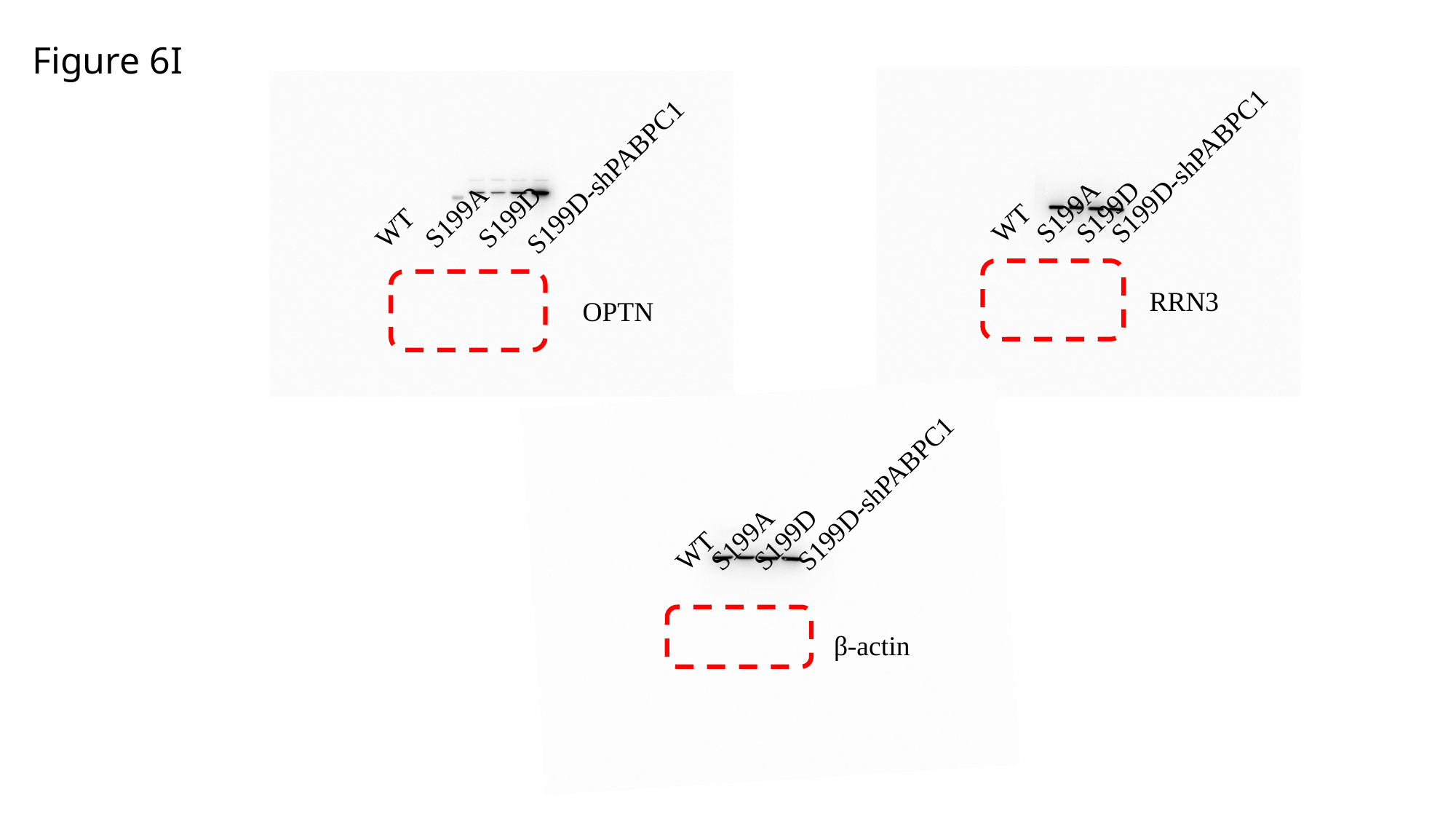

Figure 6I
WT
S199A
S199D
S199D-shPABPC1
RRN3
WT
S199A
S199D
S199D-shPABPC1
OPTN
WT
S199A
S199D
S199D-shPABPC1
β-actin

## Slide 14
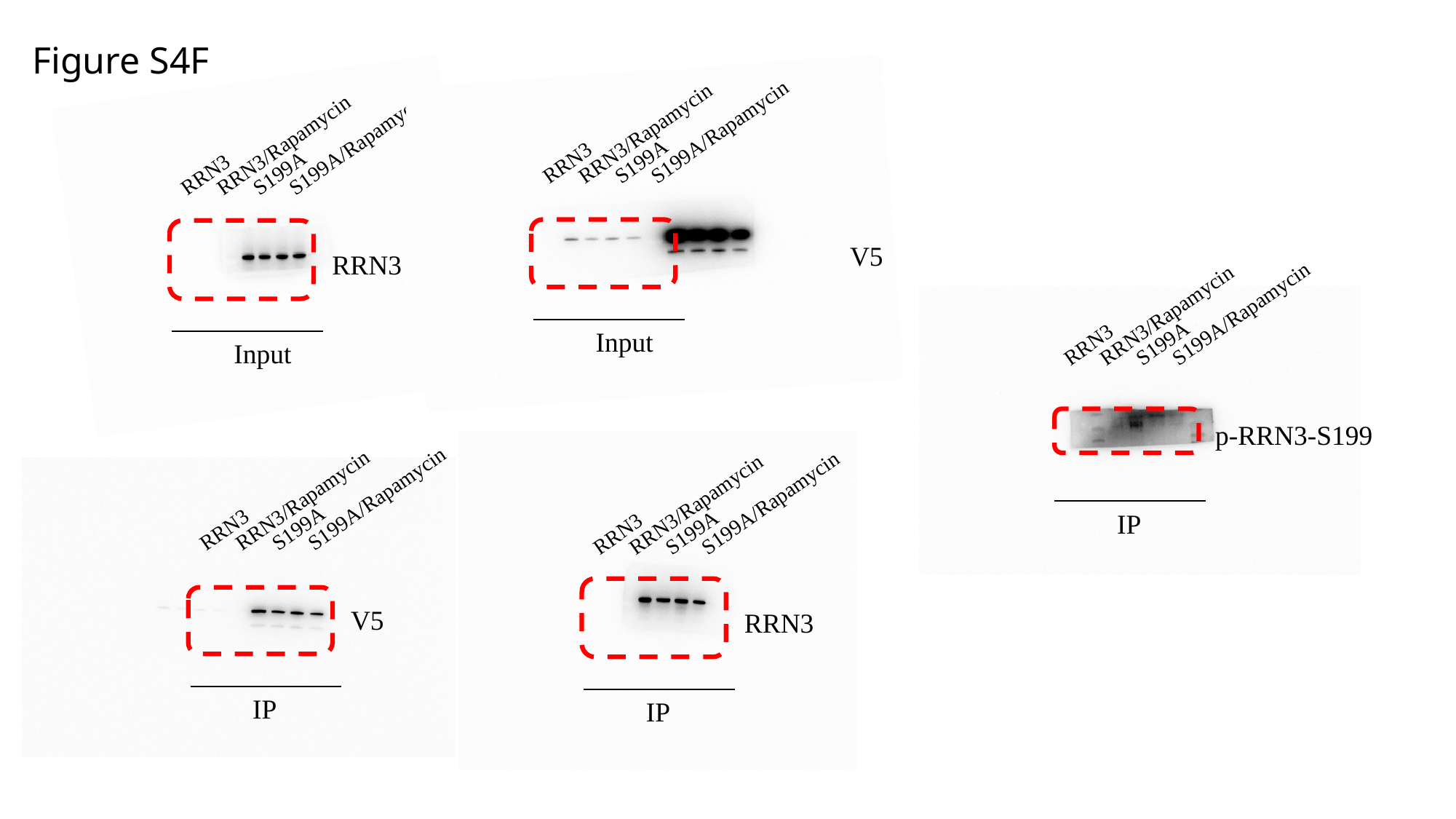

Figure S4F
S199A/Rapamycin
RRN3/Rapamycin
RRN3
S199A
V5
Input
S199A/Rapamycin
RRN3/Rapamycin
RRN3
S199A
RRN3
Input
S199A/Rapamycin
RRN3/Rapamycin
RRN3
S199A
p-RRN3-S199
IP
S199A/Rapamycin
RRN3/Rapamycin
RRN3
S199A
RRN3
IP
S199A/Rapamycin
RRN3/Rapamycin
RRN3
S199A
V5
IP

## Slide 15
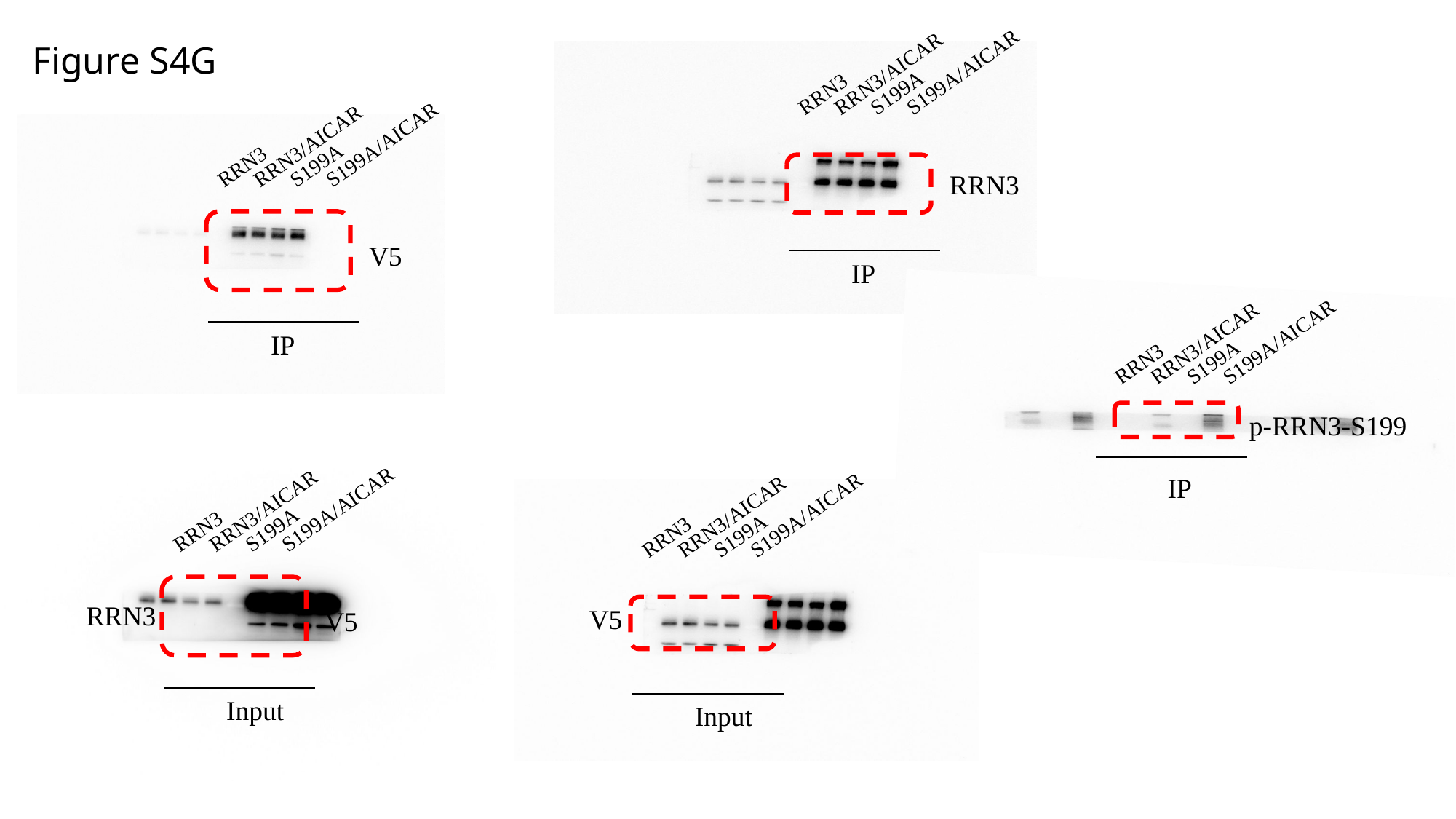

Figure S4G
S199A/AICAR
RRN3/AICAR
RRN3
S199A
RRN3
IP
S199A/AICAR
RRN3/AICAR
RRN3
S199A
V5
IP
S199A/AICAR
RRN3/AICAR
RRN3
S199A
p-RRN3-S199
IP
S199A/AICAR
RRN3/AICAR
RRN3
S199A
RRN3
V5
Input
S199A/AICAR
RRN3/AICAR
RRN3
S199A
V5
Input

## Slide 16
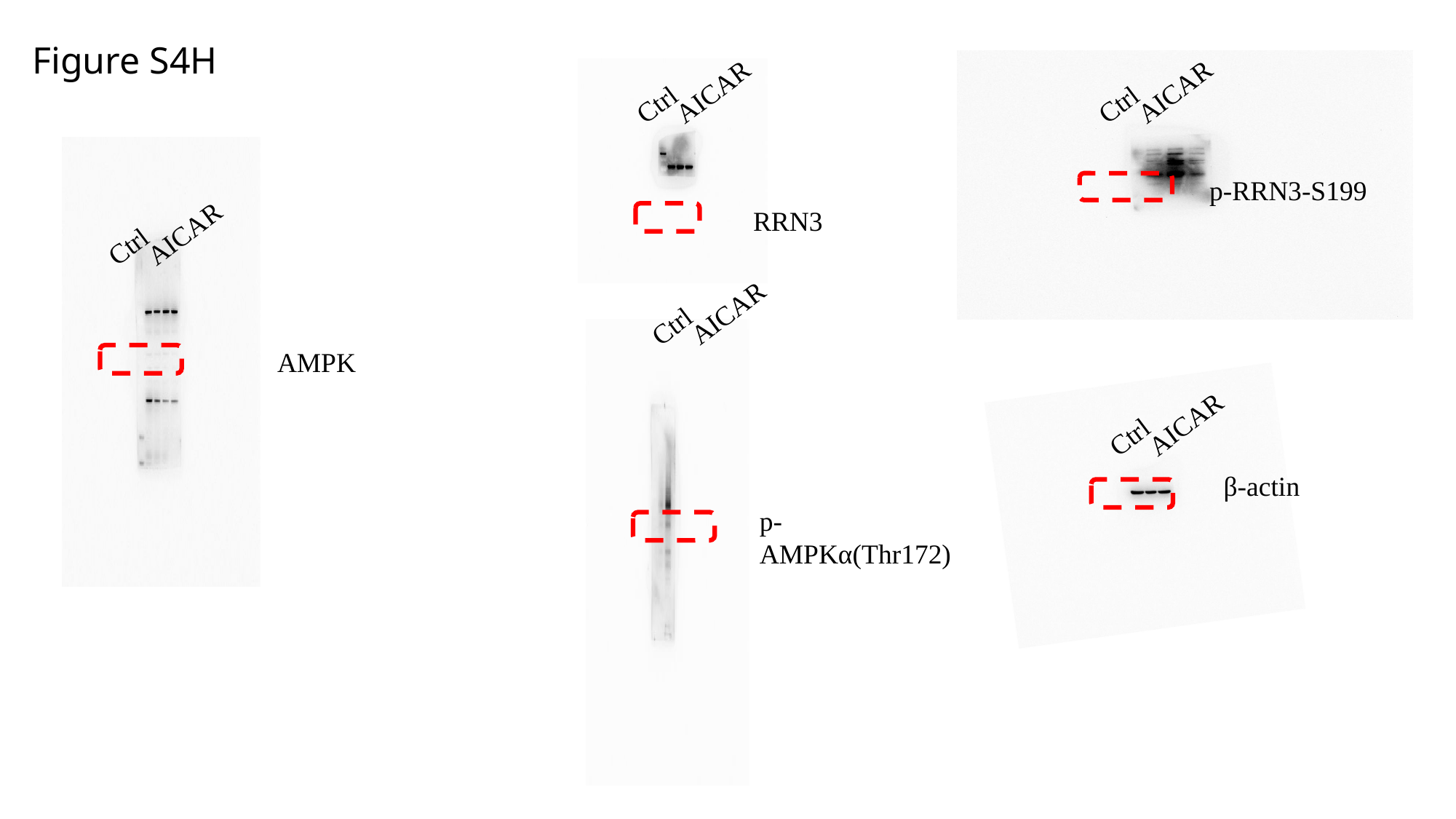

Figure S4H
AICAR
Ctrl
p-RRN3-S199
AICAR
Ctrl
RRN3
AICAR
Ctrl
AMPK
AICAR
Ctrl
AICAR
Ctrl
β-actin
p-AMPKα(Thr172)

## Slide 17
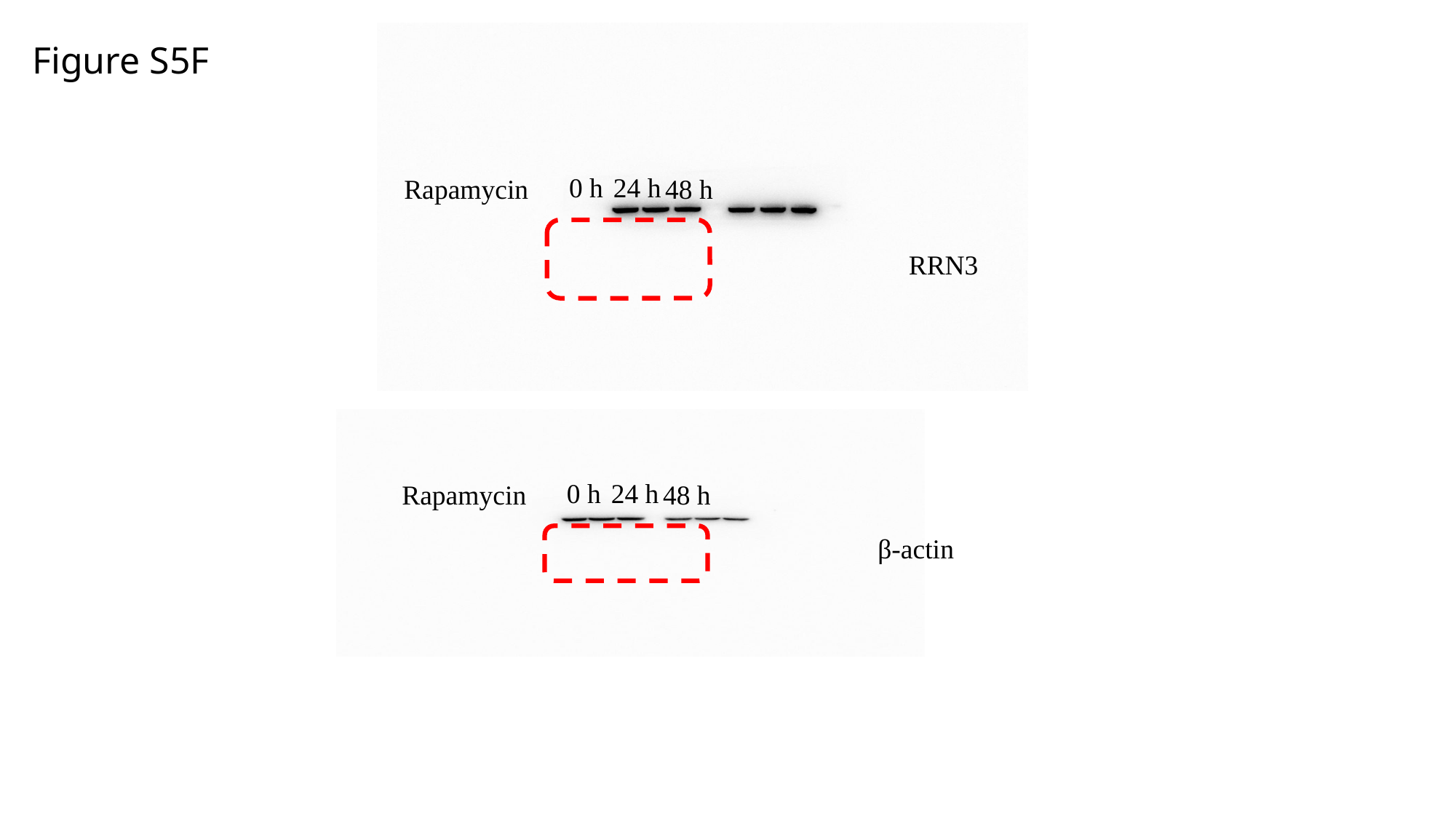

0 h
24 h
Rapamycin
48 h
RRN3
Figure S5F
0 h
24 h
Rapamycin
48 h
β-actin

## Slide 18
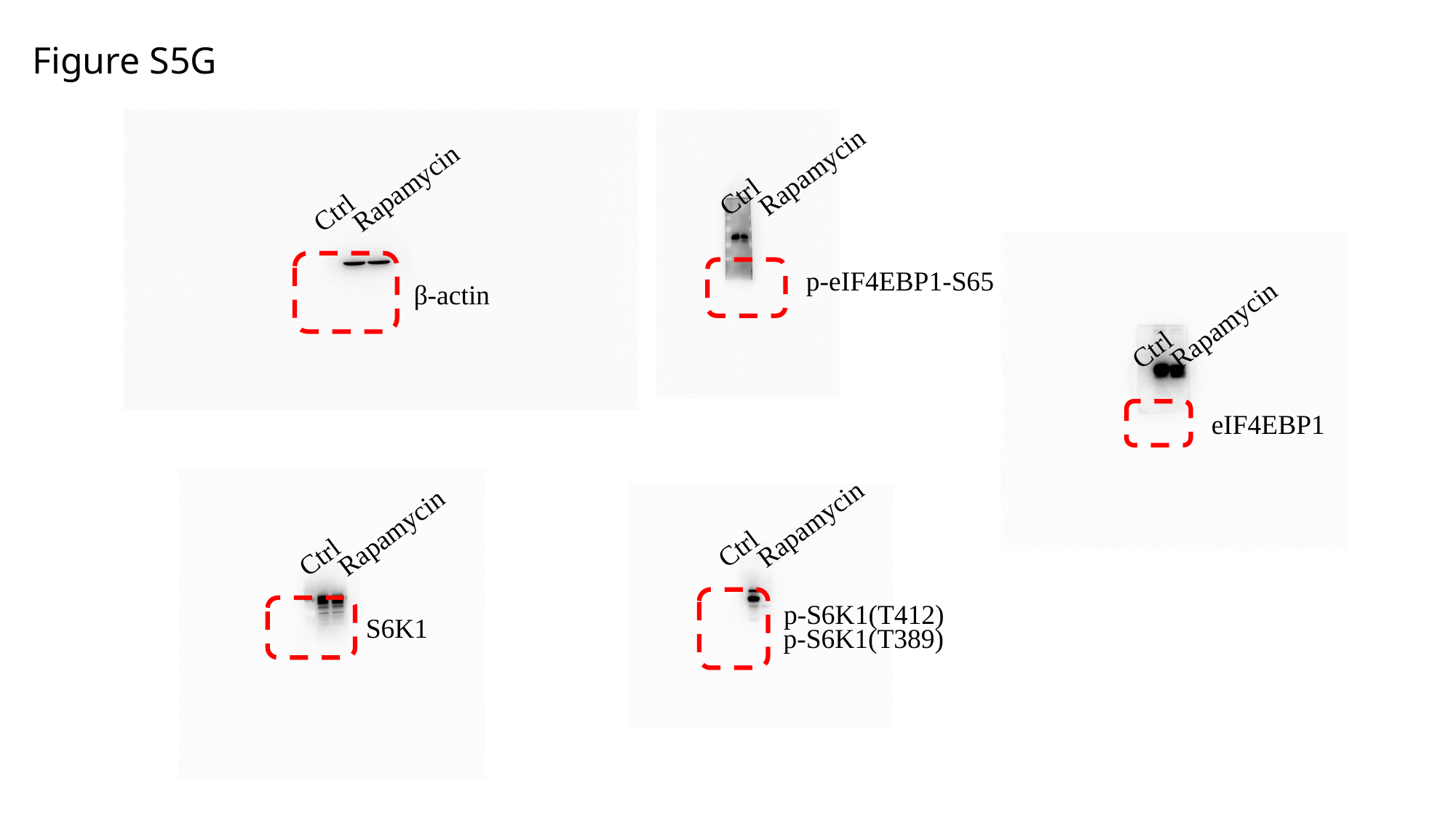

Figure S5G
Rapamycin
Ctrl
β-actin
Rapamycin
Ctrl
p-eIF4EBP1-S65
Rapamycin
Ctrl
eIF4EBP1
Rapamycin
Ctrl
S6K1
Rapamycin
Ctrl
p-S6K1(T412)
p-S6K1(T389)

## Slide 19
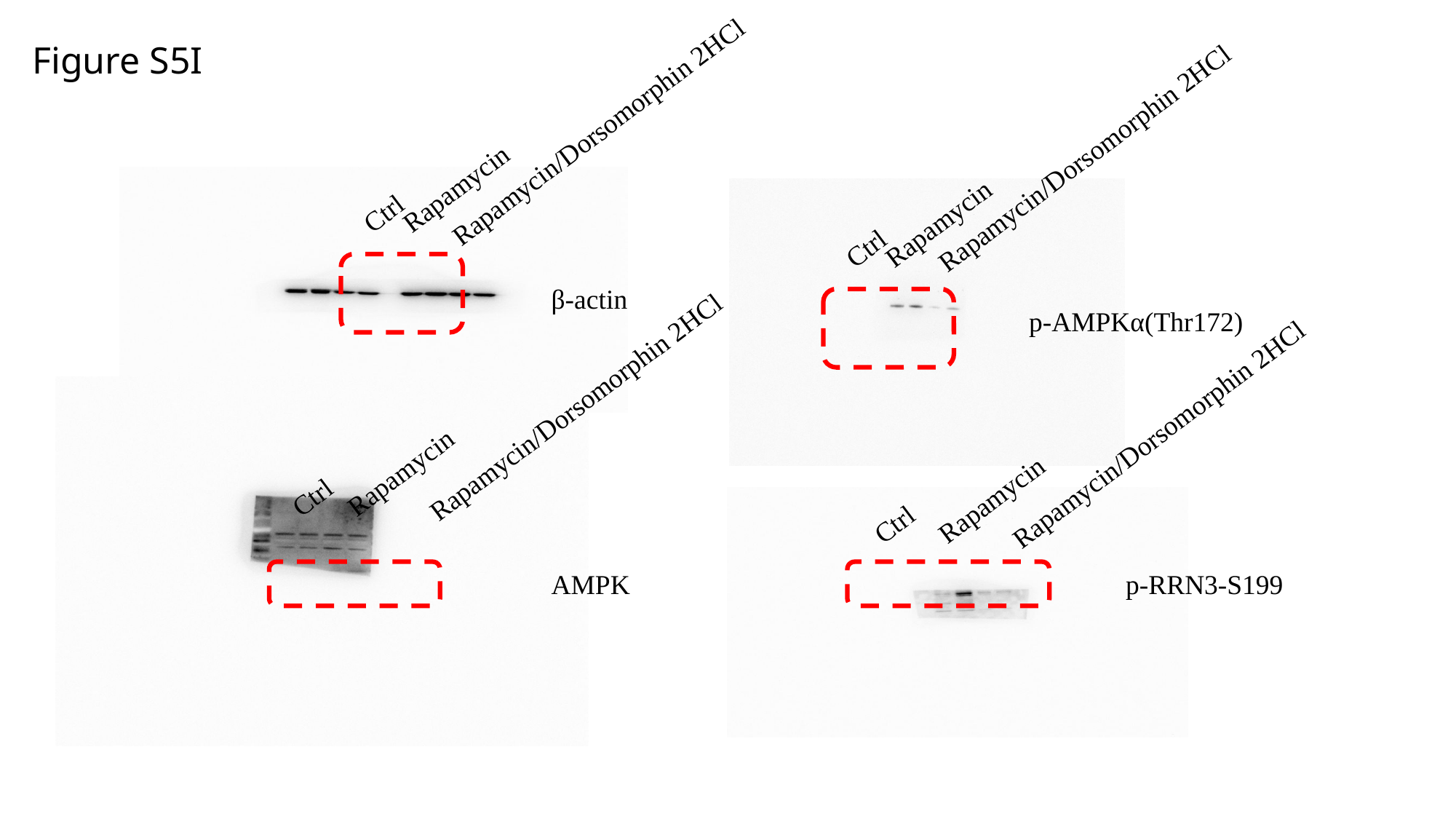

Figure S5I
Rapamycin/Dorsomorphin 2HCl
Rapamycin
Ctrl
β-actin
Rapamycin/Dorsomorphin 2HCl
Rapamycin
Ctrl
p-AMPKα(Thr172)
Rapamycin/Dorsomorphin 2HCl
Rapamycin
Ctrl
AMPK
Rapamycin/Dorsomorphin 2HCl
Rapamycin
Ctrl
p-RRN3-S199

## Slide 20
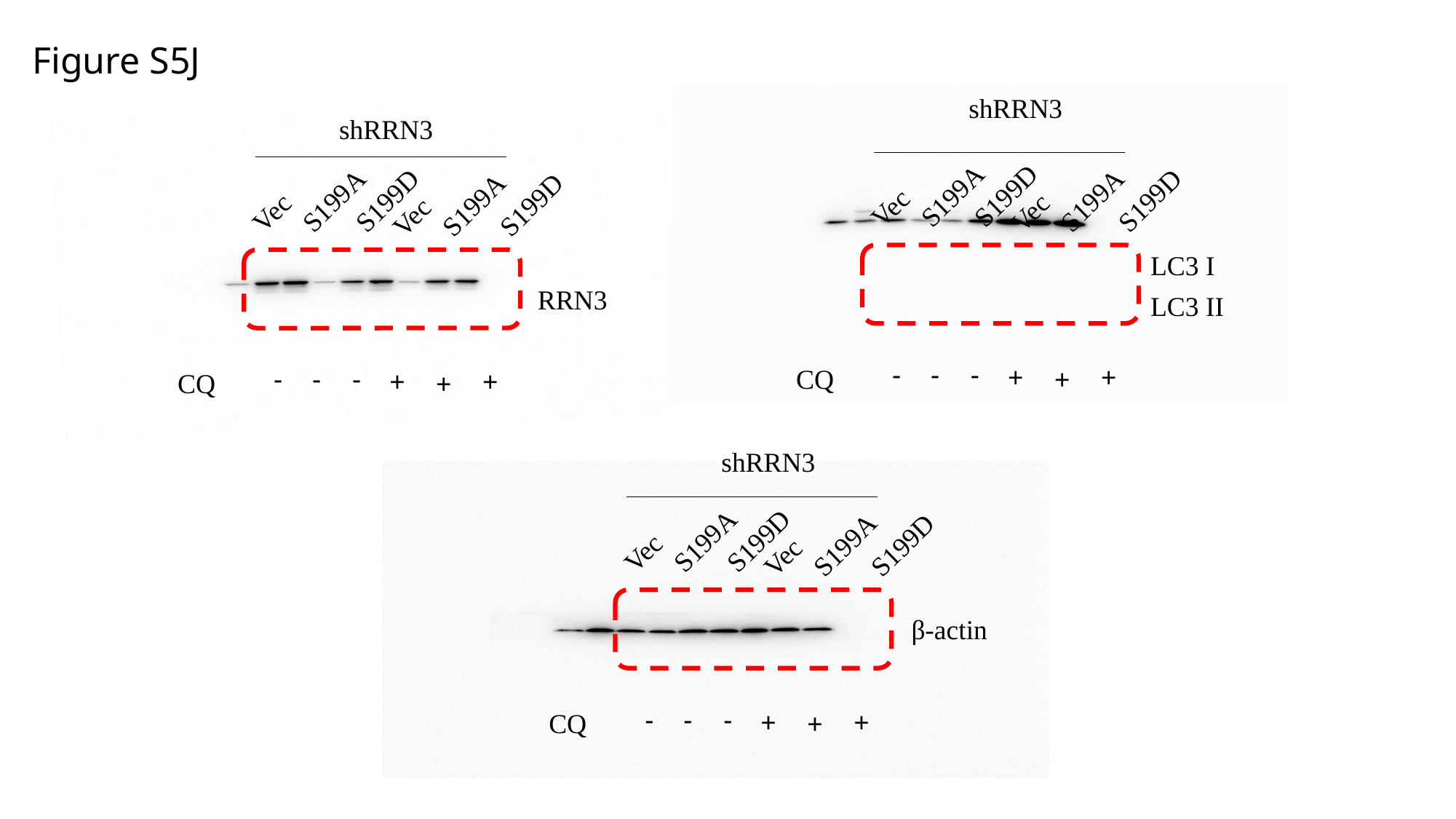

Figure S5J
shRRN3
Vec
S199A
S199D
Vec
S199A
S199D
LC3 I
LC3 II
-
-
-
+
+
CQ
+
shRRN3
Vec
S199A
S199D
Vec
S199A
S199D
RRN3
-
-
-
+
+
CQ
+
shRRN3
Vec
S199A
S199D
Vec
S199A
S199D
β-actin
-
-
-
+
+
CQ
+

## Slide 21
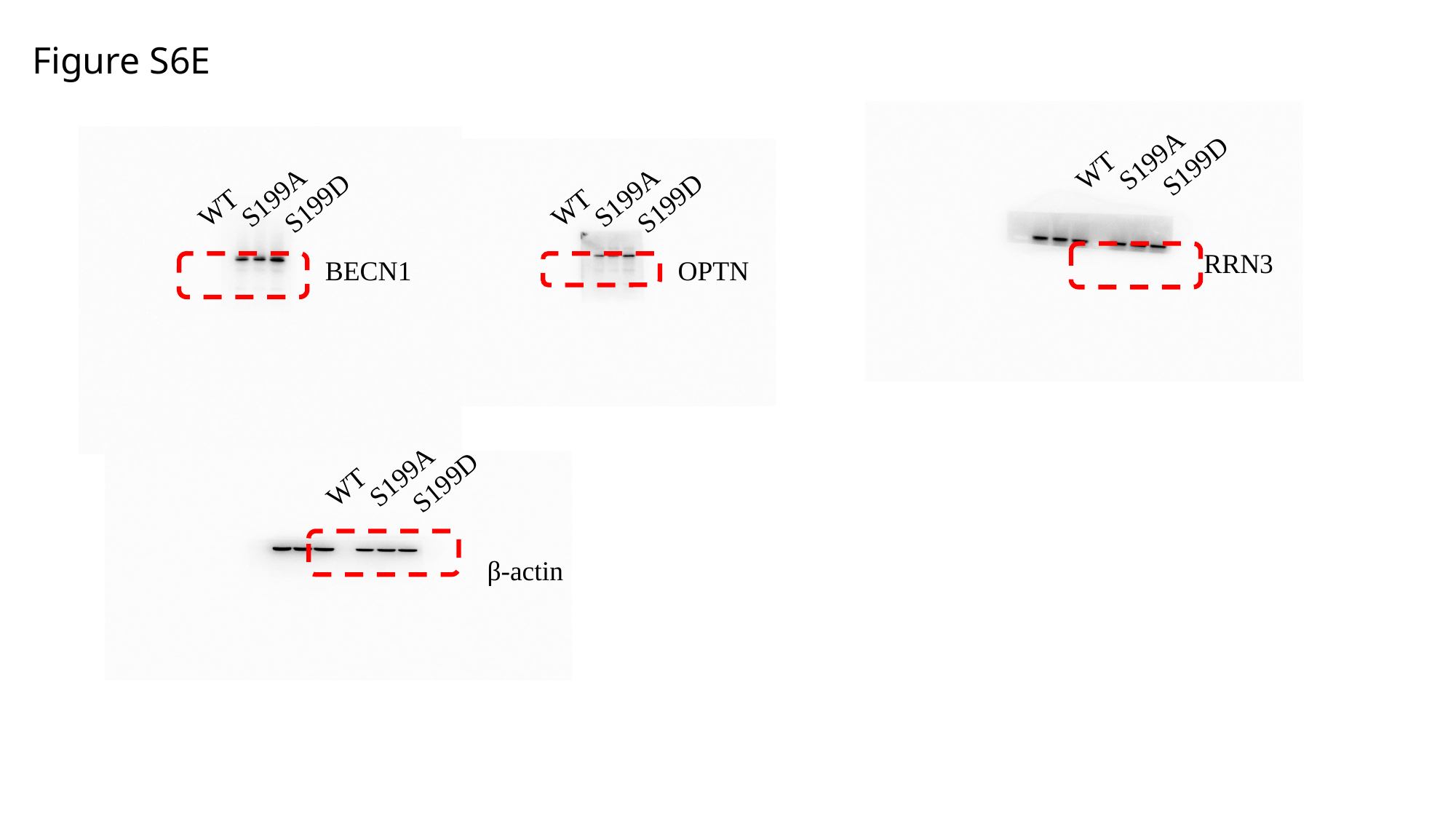

Figure S6E
S199A
S199D
WT
RRN3
S199A
S199D
WT
BECN1
S199A
S199D
WT
OPTN
S199A
S199D
WT
β-actin

## Slide 22
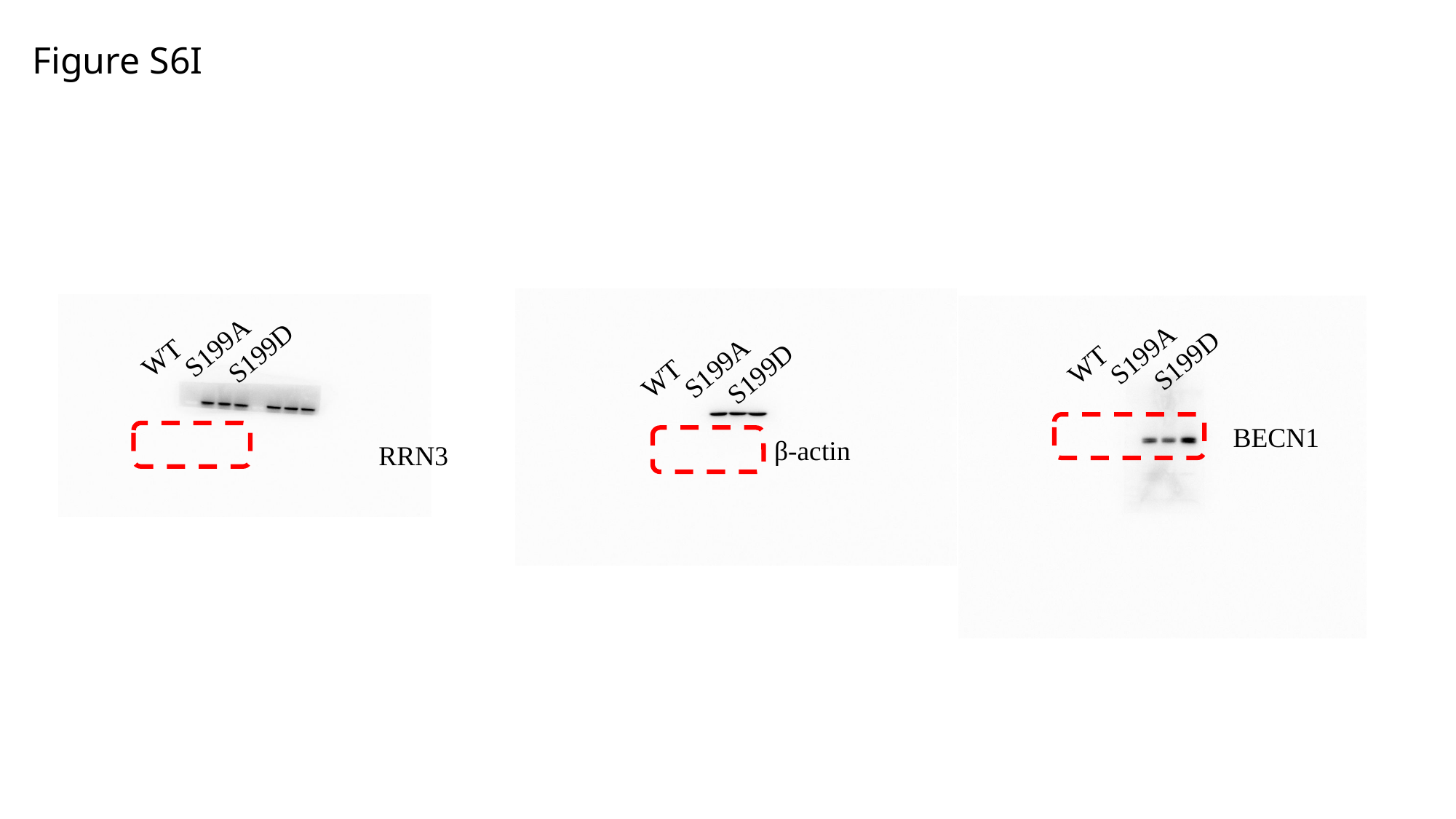

Figure S6I
S199A
S199D
WT
β-actin
S199A
S199D
WT
RRN3
S199A
S199D
WT
BECN1

## Slide 23
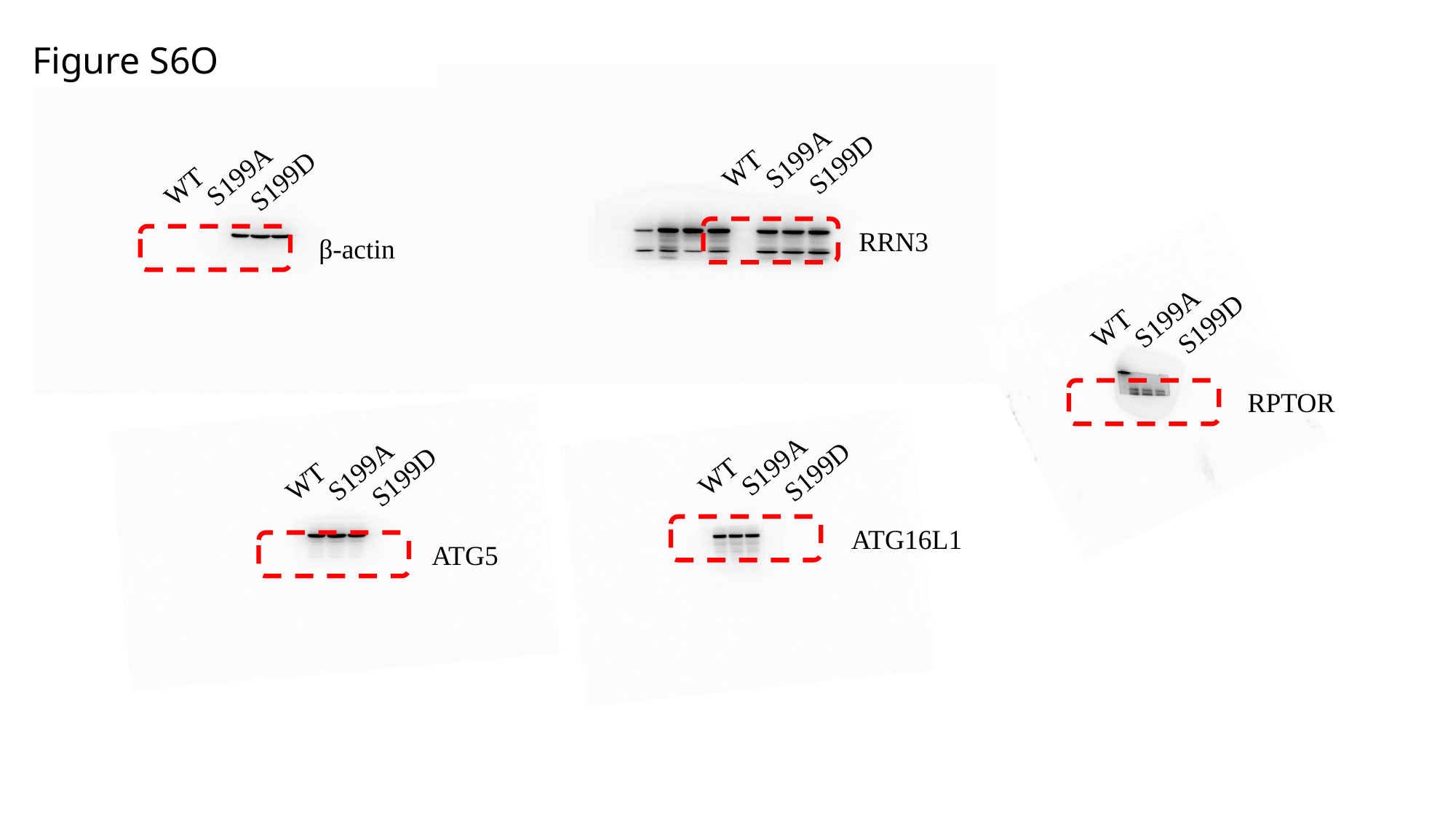

Figure S6O
S199A
S199D
WT
RRN3
S199A
S199D
WT
β-actin
S199A
S199D
WT
RPTOR
S199A
S199D
WT
ATG5
S199A
S199D
WT
ATG16L1

## Slide 24
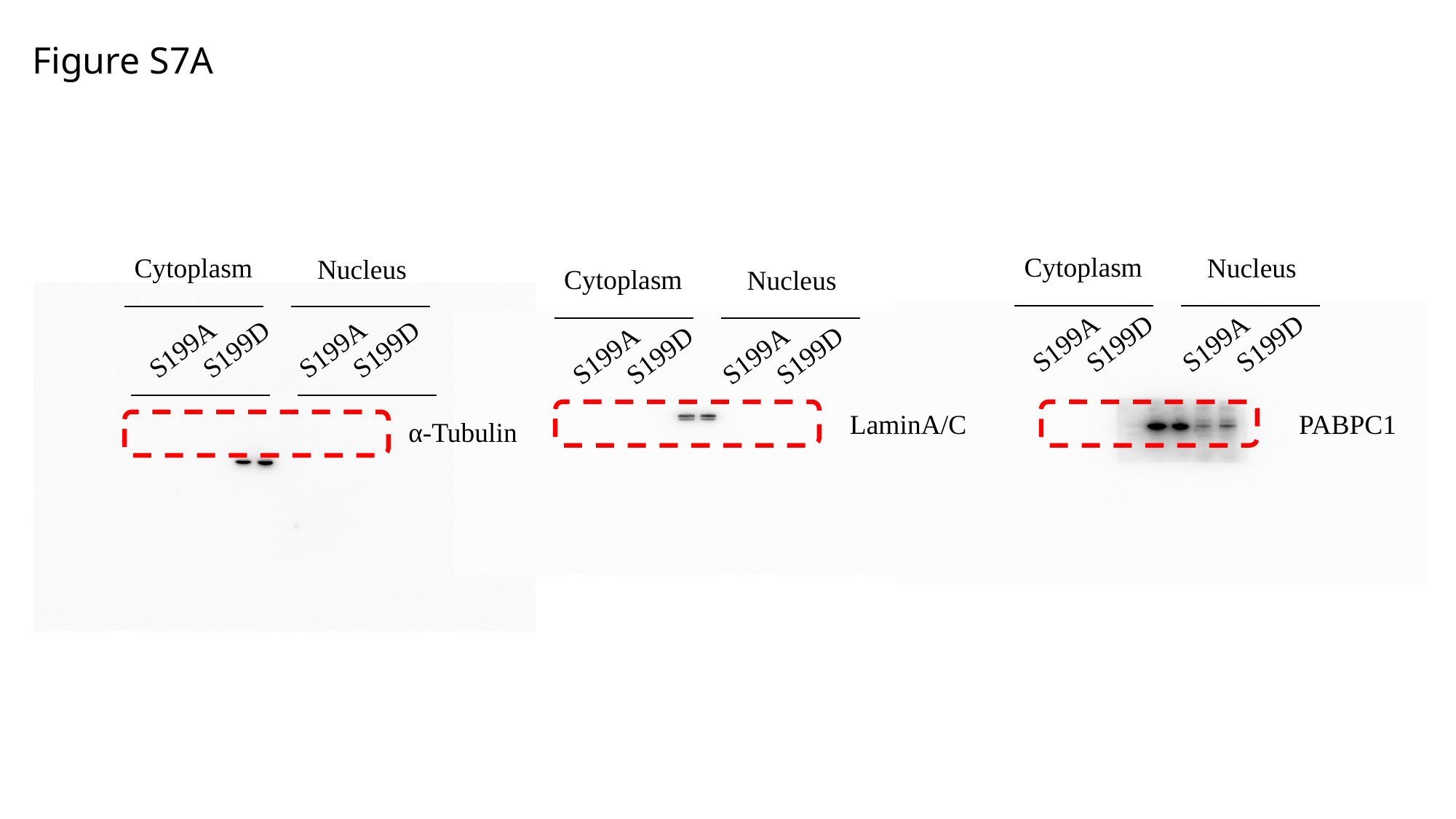

Figure S7A
Cytoplasm
Cytoplasm
Nucleus
Nucleus
Cytoplasm
Nucleus
S199A
S199D
S199A
S199D
S199A
S199D
S199A
S199D
S199A
S199D
S199A
S199D
LaminA/C
PABPC1
α-Tubulin

## Slide 25
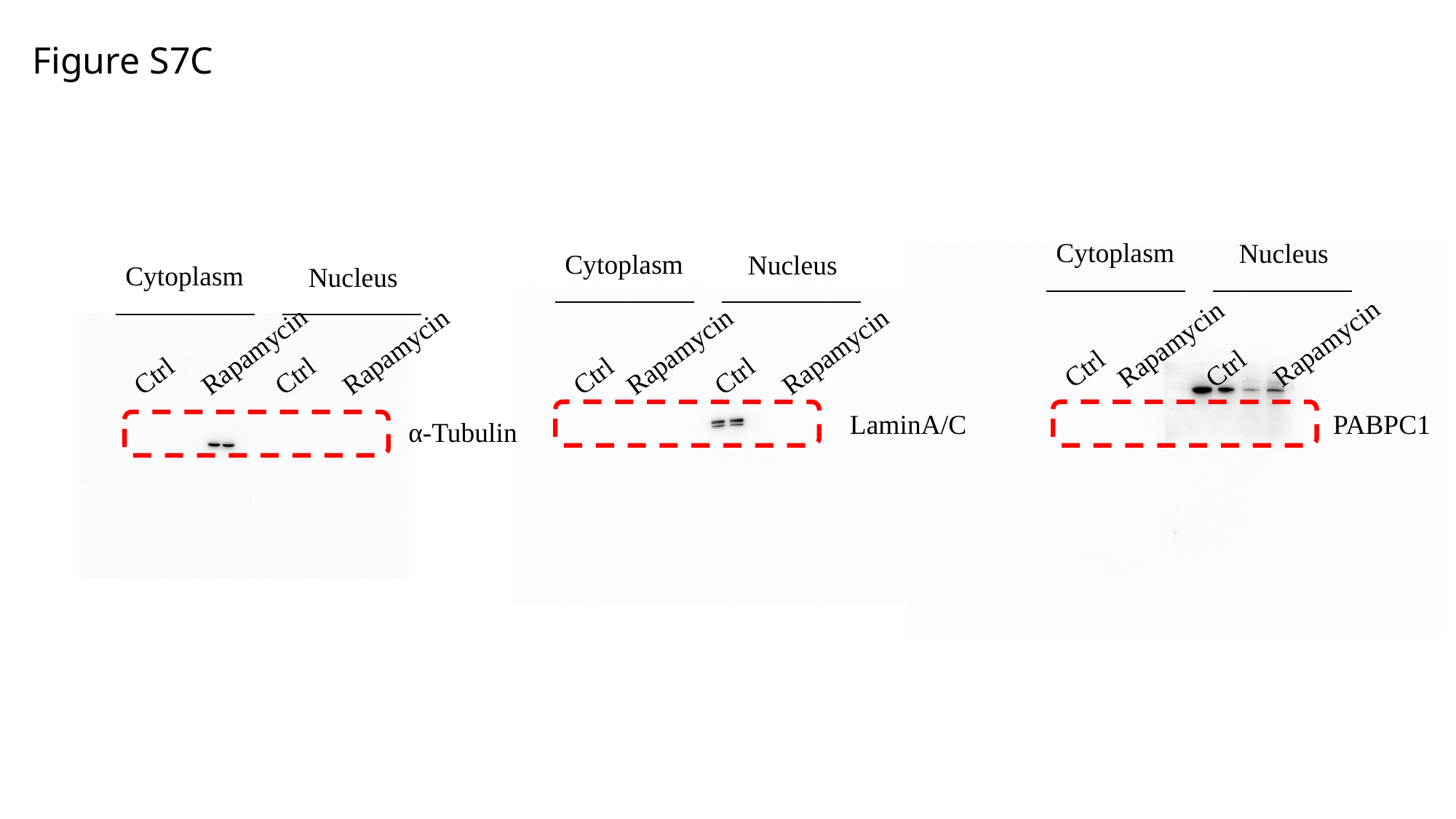

Figure S7C
Cytoplasm
Nucleus
Cytoplasm
Nucleus
Cytoplasm
Nucleus
Rapamycin
Rapamycin
Rapamycin
Rapamycin
Rapamycin
Rapamycin
Ctrl
Ctrl
Ctrl
Ctrl
Ctrl
Ctrl
LaminA/C
PABPC1
α-Tubulin
